# Harnessing multivariate, penalized regression methods for genomic prediction and QTL detection of drought-related traits in grapevine

**DOI:** 10.1093/g3journal/jkab248

**Published:** 2021-07-22

**Authors:** Charlotte Brault, Agnès Doligez, Le Cunff, Aude Coupel-Ledru, Thierry Simonneau, Julien Chiquet, Patrice This, Timothée Flutre

**Affiliations:** 1 Institut Français de la Vigne et du Vin, Montpellier F-34398, France; 2 UMR AGAP Institut, Univ Montpellier, CIRAD, INRAE, Institut Agro, Montpellier F-34398, France; 3 UMT Geno-Vigne®, IFV-INRAE-Institut Agro, Montpellier F-34398, France; 4 LEPSE, Univ Montpellier, INRAE, Institut Agro, Montpellier 34000, France; 5 AgroParisTech, UMR MIA, Paris 75005, France; 6 Université Paris-Saclay, INRAE, CNRS, AgroParisTech, GQE—Le Moulon, Gif-sur-Yvette 91190, France

**Keywords:** genomic prediction, QTL detection, multi-trait, breeding, candidate gene, water stress, grapevine

## Abstract

Viticulture has to cope with climate change and to decrease pesticide inputs, while maintaining yield and wine quality. Breeding is a key lever to meet this challenge, and genomic prediction a promising tool to accelerate breeding programs. Multivariate methods are potentially more accurate than univariate ones. Moreover, some prediction methods also provide marker selection, thus allowing quantitative trait loci (QTLs) detection and the identification of positional candidate genes. To study both genomic prediction and QTL detection for drought-related traits in grapevine, we applied several methods, interval mapping (IM) as well as univariate and multivariate penalized regression, in a bi-parental progeny. With a dense genetic map, we simulated two traits under four QTL configurations. The penalized regression method Elastic Net (EN) for genomic prediction, and controlling the marginal False Discovery Rate on EN selected markers to prioritize the QTLs. Indeed, penalized methods were more powerful than IM for QTL detection across various genetic architectures. Multivariate prediction did not perform better than its univariate counterpart, despite strong genetic correlation between traits. Using 14 traits measured in semi-controlled conditions under different watering conditions, penalized regression methods proved very efficient for intra-population prediction whatever the genetic architecture of the trait, with predictive abilities reaching 0.68. Compared to a previous study on the same traits, these methods applied on a denser map found new QTLs controlling traits linked to drought tolerance and provided relevant candidate genes. Overall, these findings provide a strong evidence base for implementing genomic prediction in grapevine breeding.

## Introduction

Viticulture is facing two major challenges, *i.e.*, coping with climate change and decreasing inputs such as pesticides, while maintaining high yield and quality. This requires understanding the physiological processes that determine adaptation to climate change, such as water use efficiency and their genetic basis (Condon *et al.* 2004). Breeding schemes could then use crosses between genotypes with high water use efficiency, and others resistant to downy and powdery mildews (Vezzulli *et al.* 2019b), to select offspring displaying the most favorable combinations. In crop species, the widespread use of molecular markers through marker-assisted selection (MAS) or genomic prediction (GP) substantially accelerates genetic gains as compared to the traditional phenotypic selection, by allowing early selection of promising genotypes, without the corresponding phenotypic information (Heffner *et al.* 2009). This is of acute interest in perennial fruit species because of the long juvenile period during which most traits of interest cannot be phenotyped. MAS and GP are now widely developed in many perennial species such as pear (Kumar *et al.* 2019), oil palm (Cros *et al.* 2015; Kwong *et al.* 2017), citrus (Gois *et al.* 2016), apple (Muranty *et al.* 2015), and *Coffea* (Ferrão *et al.* 2019). In grapevine, quantitative trait loci (QTL) detection in bi-parental populations led to the identification of major genes for traits with a simple genetic architecture such as resistance to downy and powdery mildews, berry color, seedlessness, and Muscat flavor (Fischer *et al.* 2004; Welter *et al.* 2007; Fournier-Level *et al.* 2009; Emanuelli *et al.* 2010; Mejìa *et al.* 2011; Schwander *et al.* 2012). Based on these results, most breeding efforts in grapevine use MAS to improve disease resistance. However, genetic improvement is also needed for traits with a more complex genetic determinism, as well as for others, such as drought-related traits, that are difficult to phenotype. Many minor QTLs have been found for tolerance to abiotic stresses (Marguerit *et al.* 2012; Coupel-Ledru *et al.* 2014, 2016), yield components (Doligez *et al.* 2010, 2013), and fruit quality (Huang *et al.* 2012), as reviewed in Vezzulli *et al.* (2019a). But MAS is not well suited to traits with many underlying minor QTLs (Bernardo 2008). Genomic prediction, which relies on high-density genotyping, is a promising tool for breeding for such complex traits, especially in perennial plants (Kumar *et al.* 2012). Nevertheless, in grapevine, GP has been used in three published papers, only once on experimental data (Viana *et al.* 2016a; Migicovsky *et al.* 2017) and once on simulated data (Fodor *et al.* 2014). Thus, before applying GP to this species, it has to be empirically validated by thoroughly investigating the efficiency of different methods on traits with various genetic architectures.

Both QTL detection and genomic prediction rely on finding statistical associations between genotypic and phenotypic variation. So far, QTL detection in grapevine has been mainly achieved by using interval mapping (IM) methods in bi-parental populations, or more recently genome-wide association studies (GWAS) in diversity panels [see Vezzulli *et al.* (2019a) for a comprehensive review of QTL detection studies in grapevine]. However, most quantitative traits are explained by many minor QTLs, which are difficult to detect either by IM methods or GWAS where each QTL has to exceed a significance threshold. In contrast, GP methods, by focusing on prediction, are less restrictive on the number of useful markers, sometimes resulting in all markers being retained as predictive with a nonzero effect. This is why GP methods are more efficient at predicting genotypic values (Goddard and Hayes 2007) and therefore increasingly popular with breeders (Heffner *et al.* 2010; Crossa *et al.* 2017; Kumar *et al.* 2020).

Widely used methods for GP are based on penalized regression (Hastie *et al.* 2009), notably RR [Ridge Regression, equivalent to Genomic BLUP, GBLUP, Habier *et al.* (2007)] and LASSO (Least Absolute Shrinkage and Selection Operator). Bayesian approaches are also commonly used (*e.g.*, de los Campos *et al.* 2013; Kemper *et al.* 2018), see Desta and Ortiz (2014) for a classification of GP methods. However, overall, Bayesian methods do not achieve better predictive ability than RR or LASSO, while they bear a heavy computational cost when fitted using Markov chain Monte-Carlo algorithms (Ferrão *et al.* 2019). Other methods based on nonparametric models (*e.g.*, Support Vector Machine, Reproducing Kernel Hilbert Space, Random Forest) have been shown to yield lower predictive ability than parametric models (frequentist or Bayesian) when the trait displayed an additive genetic architecture (Azodi *et al.* 2019).

Traits are often analyzed one by one in GP, using univariate methods. Nevertheless, breeders want to select the best genotypes that combine good performance for many favorable traits. Analyzing several traits jointly in GP allows taking into account any genetic correlation between traits (Henderson and Quaas 1976). Calus and Veerkamp (2011), Jia and Jannink (2012), Hayashi and Iwata (2013), and Guo *et al.* (2014) compared univariate *vs* multivariate models’ performance. They found a slight advantage for multivariate analysis when heritability was low and data were missing. Predictive ability was particularly improved if the test set had been phenotyped for one trait while prediction was applied to another correlated trait (trait-assisted prediction) as in Thompson and Meyer (1986), Jia and Jannink (2012), Pszczola *et al.* (2013), Lado *et al.* (2018), Velazco *et al.* (2019), and Liu *et al.* (2020). However, this breaks independence between the training and test sets, leading to over-optimistic prediction accuracy (Runcie and Cheng 2019). Multivariate methods have also been proposed for QTL detection by Jiang and Zeng (1995), Korol *et al.* (1995), Meuwissen and Goddard (2004), notably for distinguishing between linkage and pleiotropy when a QTL is found common to several traits. Some methods of multivariate penalized regression, such as in Chiquet *et al.* (2017), were designed to allow both QTL detection and genotypic value prediction. Multivariate GP methods are expected to perform better if traits are genetically correlated, but this remains to be confirmed with additional data. We also hypothesize that these methods will have higher QTL detection power, by making better use of information on the genetic architecture of several intertwined traits.

Methods designed for QTL detection are rarely used for genotypic value prediction. As they select only the largest QTLs, we hypothesize that these methods will provide an accurate prediction so long as the genetic architecture is simple, but would result in poor prediction performance otherwise, as determined in several studies (Heffner *et al.* 2011; Wang *et al.* 2014; Arruda *et al.* 2016). Conversely, some methods for GP, such as the LASSO and its extensions, are also able to select markers with nonnull effects, and hence to perform QTL detection. Their accuracy in detecting QTLs has been partially investigated in an inbred species by Li and Sillanpää (2012) on a single trait and simulated data and by Cho *et al.* (2010) on human data and a binary trait. Additional analyses are thus clearly needed.

This article aims to compare the ability of various methods to predict genotypic values and to detect QTLs in a bi-parental grapevine progeny, by focusing on traits related to climate change adaptation. We first complemented the only available, low density, SSR genetic map (Huang *et al.* 2012) by restriction-assisted DNA sequencing, to construct a saturated SNP map. Using this map, we then simulated phenotypic data to compare several univariate and multivariate methods and assess the impact of simulation parameters. Finally, we reanalyzed the phenotypic data on water stress from Coupel-Ledru *et al.* (2014, 2016), obtained in semi-controlled conditions. The same genotyping data and methods as those applied to simulated data were compared, providing deeper insight into the genetic determinism of key traits underlying water use efficiency, by finding new QTLs and candidate genes.

## Materials and methods

### Plant material

This study was based on a pseudo-F1 progeny of 188 offspring from a reciprocal cross made in 1995 between *Vitis vinifera* L. cultivars Syrah and Grenache (Adam-Blondon *et al.* 2005).

### GBS markers

Genotyping was done by sequencing was performed after genomic reduction, using RAD-sequencing technology, with *ApeKI* restriction enzyme (Elshire *et al.* 2011), as described in Flutre *et al.* (2020). Keygene N.V. owns patents and patent applications protecting its Sequence Based Genotyping technologies. This yielded a total number of 17,298 SNPs.

### Consensus genetic map

The genetic map was built with Lep-MAP3 (Rastas 2017), as described in https://doi.org/10.15454/QEDX2V. The resulting map had 3961 fully-informative markers (abxcd segregation) without missing data (missing marker genotypes being automatically imputed in Lep-MAP3). These data were numerically recoded in biallelic doses (0,1,2) according to the initial biallelic segregation and phase (Supplementary Table S1).

### Simulation

Phenotype simulations were carried out to (i) compare several methods for prediction accuracy, and (ii) assess the efficacy of these methods to select the markers most strongly associated with trait variation.

Two traits, $y_{1}$ and $y_{2}$ were jointly simulated according to the following bivariate linear regression model: Y=XB+E, where **Y** is the *n *×* k* matrix of traits, **X** the *n *×* p* design matrix of allelic effects, **B** the *p *×* k* matrix of allelic effects, and **E** the *n *×* k* matrix of errors. For **X**, the 3961 SNP markers mapped for the SxG progeny were encoded in four additives and four dominance effects. Therefore *n *=* *188, *k *=* *2, and *p *=* *31,688. For **B**, allelic effects corresponding to *s* additive QTLs were drawn from a matrix-variate Normal distribution, $B∼MV(0,I,V_{B})$, with **I** the *p *×* p* identity matrix and $V_{B}$ the *k *×* k* genetic variance-covariance matrix between traits such that $V_{B}=\left[\begin{array}{cc} \sigma_{B_{1}}^{2} & \rho_{B}\sigma_{B_{1}}\sigma_{B_{2}}\\ \rho_{B}\sigma_{B_{1}}\sigma_{B_{2}} & \sigma_{B_{2}}^{2} \end{array}\right]$, where *ρ_B_* is the genetic correlation among traits and $\sigma_{B_{1}}^{2}$ and $\sigma_{B_{2}}^{2}$ are the genetic variances for both traits $y_{1}$ and $y_{2}$. In the same way, $E∼MV(0,I,V_{E})$, with the *k *×* k* error variance-covariance matrix $V_{E}=\left[\begin{array}{cc} \sigma_{E_{1}}^{2} & \rho_{E}\sigma_{E_{1}}\sigma_{E_{2}}\\ \rho_{E}\sigma_{E_{1}}\sigma_{E_{2}} & \sigma_{E_{2}}^{2} \end{array}\right]$, where *ρ_E_* is the residual error correlation between traits, and $\sigma_{E}^{2}$ the error variance. We set *ρ_B_* to 0.8, $\sigma_{B_{1}}^{2}$ and $\sigma_{B_{2}}^{2}$ to 0.1, *ρ_E_* to 0, and narrow-sense heritability to 0.1, 0.2, 0.4 or 0.8, and $\sigma_{E}^{2}$ was deduced.

To explore different genetic architectures, we simulated *s *=* *2 or 50 additive QTLs, located at *s* SNP markers, so that all corresponding additive allelic effects had nonzero values in **B**. Because all allelic effects were drawn from the same distribution, all QTLs had “major” or “minor” effects for *s *=* *2 and *s *=* *50, respectively. All dominant allelic effects were set to zero. Two QTL distributions across traits were also simulated. For the first one, called “same,” all QTLs were at the same markers for both traits. For the second one, called “diff,” the two traits had no QTL in common. Thus, there was genetic correlation among traits only for the “same” QTL distribution.

For each configuration (2 or 50 QTLs, combined with “same” or “diff” distribution), the simulation procedure was replicated *t *=* *10 times, each with a different seed for the pseudo-random number generator, resulting in different QTL positions and effects.

In a first simulation set, narrow-sense heritability was assumed equal for both traits and prediction was done with all methods described below. In a second set, we simulated two traits with different heritability values (0.1 and 0.5), for the “same” QTL distribution with *s *=* *20 and *s *=* *200 QTLs, with QTL effects drawn from a matrix-variate distribution with $\sigma_{B}^{2}$=1 and *ρ_B_* = 0.5, in order to test the simulation parameters from Jia and Jannink (2012) with our genotyping data. For this second simulation set, prediction was done with a subset of methods only. Simulation parameters are summarized in Table 1.

**Table 1:** Parameter values in two sets of simulation of two traits in a bi-parental population

Simulation parameter	Same heritability values	Different heritability values
QTL number	2–50	20–200
Heritability value	0.8/0.8–0.4/0.4– 0.2/0.2–0.1/0.1	0.1/0.5
Genetic variance	0.1/0.1	1/1
Genetic correlation	0.8	0.5
QTL distribution	Same-Diff	Same

### Experimental design, phenotyping, and statistical analysis

Seven phenotypes related to drought tolerance had already been measured for 2 consecutive years on the Syrah × Grenache progeny (on 186 genotypes among the existing 188), in semi-controlled conditions on the PhenoArch platform (https://www6.montpellier.inrae.fr/lepse_eng/M3P, last accessed on 07-21-21) in Montpellier, France, as detailed in Coupel-Ledru *et al.* (2014, 2016). Briefly, of all replicates (six and five per genotype respectively in 2012 and 2013), three (in 2012), or two (in 2013) were maintained under well-watered conditions (well-watered condition, WW), whereas the other three were submitted to a moderate water deficit (water deficit condition, WD). Specific transpiration, *i.e.* transpiration rate per leaf area unit, was measured during daytime (*TrS*) and night-time (TrS_night). Midday leaf water potential (*ψ_M_*, *PsiM*) was also measured and the difference between soil and leaf water potential (Δψ, *DeltaPsi*) calculated. Soil-to-leaf hydraulic conductance on a leaf area basis (*KS*) was calculated as the ratio between *TrS* and *DeltaPsi*. Growth rate (*DeltaBiomass*) was estimated by image analysis. Transpiration efficiency (TE) was calculated over a period of 10 to 15 days as the ratio between growth and total water loss through transpiration during this period.

These seven phenotypes were studied under each watering condition (WW and WD). We thus considered 14 traits in this study, a trait being defined as a phenotype × watering condition combination, and used the raw data available online (https://doi.org/10.15454/YTRKV6). For each trait, a linear mixed model was fitted with R/lme4 version 1.1-21 (Bates *et al.* 2014) using data from both years:

(1) $$\begin{array}{c} y=\mu +Y+R+x_{g}+y_{g}+x_{c}+y_{c}+O+C+D\\ \underline{G}+\underline{G:Y}+\underline{G:D}+\epsilon \end{array}$$

First, model 1 with nine fixed effects (*Y* year, *R* replicate, *x_g_*, *y_g_* coordinates in the platform within the greenhouse, *x_c_*, *y_c_* coordinates in the controlled-environment chamber where *PsiM* and *TrS* were measured, *O* operator for *PsiM* measurements, *C* controlled-environment chamber and *D* date of measurement) and three random effects (*G* genotype, G:Y genotype-year, and G:D genotype-date interactions) was fitted with maximum likelihood (ML). The R/lme4 output was given to R/lmerTest version 3.1-2 (Kuznetsova *et al.* 2017) to use its function “step.” Backward elimination of random-effect terms was performed using likelihood ratio test, followed by backward elimination of fixed-effect terms using *F*-test for all marginal terms, *i.e.*, terms that can be dropped from the model while respecting the hierarchy of terms in the model (Kuznetsova *et al.* 2017).

The final model after backward elimination was then fitted with restricted maximum likelihood (ReML) to obtain unbiased estimates of the variance components and empirical BLUPs (Best Linear Unbiased Predictions) of the genotypic values. The acceptability of underlying assumptions (homoscedasticity, normality, independence) was visually assessed by plotting residuals and BLUPs. Broad-sense heritability on a genotype-mean basis was computed assuming a balanced design [see the introduction of Piepho and Möhring (2007)], as:

(2) $$H^{2}=\frac{\sigma_{G}^{2}}{\sigma_{G}^{2}+\frac{\sigma_{G:Y}^{2}}{{\bar{n}}_{\mathrm{year}}}+\frac{\sigma_{e}^{2}}{{\bar{n}}_{\mathrm{year}}\times {\bar{n}}_{\mathrm{rep}}}},$$

with $\sigma_{G:Y}^{2}$ the genotype-year interaction variance, $\sigma_{e}^{2}$ the residual variance, ${\bar{n}}_{\mathrm{year}}$ the arithmetic mean number of trials (years) and ${\bar{n}}_{\mathrm{rep}}$ the mean number of replicates per trial. Its coefficient of variation was estimated by bootstrapping with R/lme4 and R/boot packages.

### Comparison of genotypic BLUPs

We first recomputed genotypic BLUPs from the raw phenotypic data of Coupel-Ledru *et al.* (2014, 2016) in order to control the model selection step in a reproducible way. These new BLUPs had a strong linear correlation (>0.9) with those used in Coupel-Ledru *et al.* (2014, 2016), as shown in Supplementary Figure S2. The estimates of broad-sense heritability followed the same trend as in Coupel-Ledru *et al.* (2014, 2016) (Supplementary Figure S3). They were higher in WD condition than in WW condition for all traits except *DeltaBiomass*.

Genetic correlation between traits varied widely, some absolute correlation values being very high (*e.g.*, up to 0.99 between *PsiM* and *DeltaPsi* in both conditions) when traits derived from others (Supplementary Figure S4).

### Interval mapping methods

Two univariate IM methods were compared, using R/qtl version 1.46-2 (Broman *et al.* 2003). For both, the probability of each genotypic class was first inferred at markers and every 0.1 cM between markers along with the genetic map, using the R/qtl::calcgenoprob function.

#### Simple interval mapping:

Simple interval mapping (SIM, Lander and Botstein 1989) assumes that there is at most one QTL per chromosome. A LOD score was computed every 0.1 cM with R/qtl::scanone, then 1000 permutations were performed to determine the LOD threshold so that the family-wise (genome wide) error rate (FWER) was controlled at 5%.

#### Multiple interval mapping:

Multiple interval mapping (MIM, Kao *et al.* 1999) allows the simultaneous detection of several QTLs. It was performed with R/qtl::stepwiseqtl, using a forward/backward selection of Haley-Knott regression model (Haley and Knott 1992), with a maximum number of QTLs set to 4 (or 10 for ROC curve construction, see below), replicated 10 times to overcome occasional instability issues. Only main effects were included (no pairwise QTL × QTL interaction). The LOD threshold was computed with permutations (1000 for QTL detection and 10 for cross-validation of GP, see below) to determine the main penalty with R/qtl::scantwo. QTL positions and effects were determined with R/qtl::refineqtl and R/qtl::fitqtl, respectively. For both methods, QTL positions were determined as those of LOD peaks above the threshold, with LOD-1 confidence intervals (Lander and Botstein 1989).

#### Penalized regression methods:

Genomic prediction can be seen as a high-dimension regression problem with more allelic effects (in **B**) to estimate than observations (in **Y**), known as the “n≪p” problem. The likelihood of such models must be regularized and various extensions, called penalized regression of the Ordinary Least Squares (OLS) algorithm were proposed. Such penalization generally induces a bias in the estimation of allelic effects.

### Univariate methods

#### Ridge regression:

Ridge regression (RR, Hoerl and Kennard 1970) adds to the OLS a penalty on the effects using the *L*2 norm and solves the following equation: $\hat{\beta_{RR}}=\mathrm{argmi}n_{\lambda }||Y-X\beta |{|}_{2}^{2}+\lambda ||\beta |{|}_{2}^{2}$. As a result, all estimated allelic effects are shrunk toward zero, yet none is exactly zero. The amount of shrinkage is controlled by a regularization parameter (*λ*). We tuned it by cross-validation using the cv.glmnet function of the R/glmnet package version 3.0-2 (Friedman *et al.* 2010) with default parameters, except family =“gaussian” and *α*  =  0, keeping the *λ* value that minimizes the mean square error (MSE). Note that effects associated with correlated predictors are averaged so that they are close to identical, for a high level of regularization.

#### Least absolute shrinkage and selection operator:

Least absolute shrinkage and selection operator (LASSO, Tibshirani 1996) adds to the OLS a penalty on the effects using the *L*1 norm and solves the following equation: $\hat{\beta_{\mathrm{LASSO}}}=\mathrm{argmi}n_{\lambda }||Y-X\beta |{|}_{2}^{2}+\lambda ||\beta |{|}_{1}$. As a result, some allelic effects are exactly equal to zero, while others are shrunk toward zero. Hence LASSO performs predictor selection, *i.e.*, provides a sparse solution of predictors included in the best model, in addition to estimating their allelic effect. The LASSO regularization parameter (*λ*) was tuned by cross-validation with cv.glmnet function (family =“gaussian,” *α*  =  1), as above. If *n *<* p*, LASSO selects at most *n* predictors.

#### Extreme gradient boosting:

We first applied LASSO for dimension reduction and then Extreme Gradient Boosting, a popular machine learning method (Mason *et al.* 1999), to estimate marker effects. Hence, we called this method LASSO.GB. As gradient boosting is a nonlinear method, it can take into account any nonlinear interaction between markers, providing better prediction. Briefly, Extreme Gradient Boosting iteratively updates the estimation of weak predictors, in order to reduce the loss. This method requires an optimization of many parameters associated with the loss function (MSE). This optimization was done with train function from R/caret package version 6.0-86 (Kuhn 2008) using the “xgbTree” method. As the optimization of numerous parameters was computationally heavy, we fixed some of them (nrounds = max_depth = 2, colsample_bytree = 0.7, gamma = 0, min_child_weight = 1 and subsample = 0.5), while testing a grid of varying parameters (nrounds = 25, 50, 100, 150; eta = 0.07, 0.1, 0.2).

#### Elastic net:

Elastic net (EN, Zou and Hastie 2005) adds to the OLS both *L*1 and *L*2 penalties, the balance between them being controlled by a parameter (*α*); it solves the following equation: $\hat{\beta_{EN}}=\mathrm{argmi}n_{\lambda }||Y-X\beta |{|}_{2}^{2}+(1-\alpha )\lambda ||\beta |{|}_{2}^{2}+\alpha \lambda ||\beta |{|}_{1}$. Both *α* and *λ* were tuned by nested cross-validation: 20 values of *α* were tested between 0 and 1 and, for each of them, we used cv.glmnet function to choose between 500 values of *λ*. EN performs predictor selection but is less sparse than LASSO.

Note that RR, LASSO, and EN all assume a common variance for all allelic effects.

### Multivariate methods

#### Multi-task group-EN:

Multi-task group-EN (MTV_EN, Hastie and Qian 2016) is a multivariate extension of EN, it solves the following equation: $\hat{B_{\mathrm{MTV}\_EN}}=\mathrm{argmi}n_{\lambda }||Y-X\beta |{|}_{F}^{2}+(1-\alpha )\lambda ||\beta |{|}_{F}^{2}+\alpha \lambda ||\beta |{|}_{2}$, F being the Frobenius norm. It assumes that each predictor variable has either a zero or nonzero effect across all traits, allowing nonzero effects to have different values among traits. *λ* and *α* parameters were tuned using cv.glmnet (family = “mgaussian”). MTV_RR is the multivariate extension of RR, also tuned with cv.glmnet (family = “mgaussian,” *α*  =  0). MTV_LASSO is the multivariate extension of LASSO, also tuned with cv.glmnet (family = “mgaussian,” *α*  =  1). The implementation of these three methods is identical.

#### The multivariate structured penalized regression:

The multivariate structured penalized regression (called SPRING in Chiquet *et al.* 2017) applies a L1−penalty (*λ*_1_ parameter) for controlling sparsity (like LASSO) and a smooth L2−penalty (*λ*_2_ parameter) for controlling the amount of structure among predictor variables (*L*) to add in the model, *i.e.*, the correlation between markers according to their position on the genetic map. Both parameters *λ*_1_ and *λ*_2_ were tuned by cross-validation using cv.spring function (from R/spring package, version 0.1-0). The regression equation can be written as: Y=XB+ϵ with ϵ∼N(0,R), *R* is the covariance matrix of residuals (Gaussian noise). The allelic effects are: $B=-\Omega_{Xy}\Omega_{yy}^{-1}$ and they comprise both direct effects Ω_*Xy*_ and indirect ones Ω_*yy*_.

SPRING solves the following equation: $(\hat{\Omega_{Xy}},\hat{\Omega_{yy}})=\mathrm{argmin}-\frac{1}{n}\text{log}\;\ell (\Omega_{Xy},\Omega_{yy})+\frac{\lambda_{2}}{2}tr(\Omega_{yX}L\Omega_{Xy}\Omega_{yy}^{-1})+\lambda_{1}||\Omega_{Xy}|{|}_{1}$. Unlike multi-task group-EN, SPRING selects specific predictors for each trait, *i.e.*, a selected predictor can have a nonzero effect for a subset of the traits. Moreover, SPRING allows the distinction between direct and indirect effects by using conditional Gaussian graphical modeling. These effects are due to covariance of the noise such as environmental effects affecting several traits simultaneously. This distinction results in two kinds of estimated allelic effects: the direct ones, re-estimated with OLS, which are best suited for QTL detection (we called the corresponding prediction method *spring.dir.ols*) and the regression ones, which involve both direct and indirect effects and are best suited for prediction (*spring.reg* method).

### Robust extension for marker selection

To enhance the reliability of marker selection by penalized methods, we used two approaches: stability selection (SS) (Meinshausen and Buhlmann 2009) and marginal False Discovery Rate (Breheny 2019), both of which aim at controlling the number of false-positive QTLs. We did not use these methods for genomic prediction, as they are not designed for this purpose.

#### Stability selection:

SS is a method that controls FWER, it computes the empirical selection probability of each predictor by applying a high-dimensional variable selection procedure, *e.g.*, LASSO, to a different subset of half the observations for all *λ* values from a given set, and then retains only predictors with a selection probability above a user-chosen threshold. SS is implemented in R/stabs package version 0.6-3 (Hofner and Hothorn 2017) and can also be adapted to a multivariate framework. For QTL detection on experimental data, the probability threshold we applied was 0.6 for LASSO.SS and 0.7 for MTV_LASSO.SS.

#### Marginal false discovery rate:

Marginal false discovery rate (mFDR) has been defined by Breheny (2019) as a modified version of the FDR in which those variables correlated with the causative features are not considered as false discovery. This study provided an accurate estimation of mFDR for a given *λ* when using EN or LASSO, thus allowing the selection of a more conservative value of *λ* in order to remain below a given mFDR threshold. We applied mFDR with the R/nvcreg package version 3.12.0 (Breheny 2019). For QTL detection on experimental data, we set mFDR to 10% for LASSO.mFDR and EN.mFDR. To our knowledge, this approach had not been adapted yet to a multivariate framework.

### Evaluation and comparison of methods

All methods were compared on two aspects: their ability to predict genotypic values, and their ability to select relevant markers, *i.e.*, to detect QTLs. To assess the prediction of genotypic values on simulated data, we used the Pearson’s correlation coefficient between predicted and simulated genotypic values (prediction accuracy). On experimental data, we used the same criterion, but the actual genotypic values being unknown, we used their empirical BLUPs instead (predictive ability).

For QTL detection on simulated data, the methods were compared using criteria of binary classification based on the numbers of true positives and false negatives. On experimental data, because true QTLs are unknown, no such comparison could be performed; instead, we compared the outcome of the different methods and QTLs were deemed reliable when found with several methods.

### Genomic prediction

A nested cross-validation (CV) was applied to assess prediction by the various methods.

- An outer k1−fold CV was performed to estimate the performance metrics, with an inner k2−fold CV applied to the training set of each outer fold to find the optimal tuning parameters for the method under study (Supplementary Figure S5). Both k1 and k2 were set to 5 (see Arlot and Lerasle (2016). The partitions of the outer CV were kept constant among traits and methods.
- For IM methods, the optimal tuning parameter was the LOD threshold obtained from permutations, and the effects for the four additive genotypic classes (ac, ad, bc, and bd) were estimated by fitting a multiple linear regression model with genotype probabilities at all QTL peak positions as predictors, using R/stat::lm. For penalized regression methods, parameters were optimized with specific functions such as cv.glmnet and cv.spring.
- As performance metrics, we used mainly Pearson’s correlation (corP) but we also calculated the root mean square predicted error (RMSPE), Spearman correlation (corS), the model efficiency (Mayer and Butler 1993), and test statistics on bias and slope from the linear regression of observations on predictions (Piñeiro *et al.* 2008).

For experimental data, the whole nested cross-validation process was repeated 10 times (*r *=* *10), whereas for simulated data it was performed only once, but on 10 different simulation replicates (*r *=* *1 and *t *=* *10). The 14 traits were analyzed jointly for MTV_RR, MTV_LASSO, and MTV_EN. But for SPRING, since analyzing all traits together was computationally too heavy, we split traits into three groups by hierarchical clustering (Supplementary Figure S6) performed with R/hclust applied to genotypic BLUPs. All traits within a given cluster were analyzed together.

For simulated data with the same heritability values for both traits, performance results were averaged not only over simulation replicates and partitions of outer CV, but also over traits, because both traits were equivalent in terms of simulation parameters. For simulated data with different heritability values, performance results were averaged only over simulation replicates and partitions of outer CV. For experimental data, performance results were averaged over partitions of outer CV and outer CV repetitions.

### QTL detection

#### Simulated data:

The quality of a predictor selection method is usually assessed through the relationship between statistical power (*i.e.*, the true positive rate, TPR) and type I error rate (*i.e.* the false positive rate, FPR). To compare methods, we thus used the ROC (receiver operating characteristic) curve (Swets *et al.* 1979), which is the plot of TPR as a function of FPR over a range of parameters (Table 2), and the pAUC (partial Area Under the Curve; McClish 1989; Dodd and Pepe 2003). Any marker selected at ±2 cM of a simulated QTL was counted as a True Positive.

**Table 2:** Parameter ranges for ROC curve computation, for comparing predictor selection performance of different methods

Method	SIM/MIM	LASSO/MTV_LASSO	Stability Selection	SPRING	EN	mFDR
Parameter name	*LOD*	*λ*	*probability threshold*	*λ_1_*	*λ*	*mFDR*
Lowest constraint	0	10e-5	0.5	10e-8	10e-4	0.3
Highest constraint	14	0.25	0.9	0.25	8	0

For methods with two tuning parameters, one parameter was kept constant (*α* at 0.7 for EN and EN.mFDR, and *λ*_2_ at 10e-8 for SPRING). We tested several *α* values for EN but it did not change much the results (data not shown). For MIM, the maximum number of QTLs that can be integrated into the model was set to 10.

#### Experimental data:

Comparison between methods was based on the number of detected QTLs, the magnitude of their effects, and the percentage of variance globally explained by all detected QTLs.

For MTV_LASSO and SPRING, we split traits into three groups as described above, for computational reasons (for SPRING) and to test whether such splitting evidenced more reliable QTLs (for MTV_LASSO). The parameters of penalized methods were tuned by cross-validation, with MSE as the cost function. We compared predictor selection between methods in terms of the number of common selected markers per trait, *i.e.*, the intersection between markers selected by penalized methods and markers inside confidence intervals found by IM methods. Then all markers in high LD with those selected were considered as selected too. The threshold was defined as the 95% quantile of LD value distribution, for all pairs of markers belonging to the same chromosome (Supplementary Figure S7), which gave a LD threshold of 0.84.

We deemed selected markers as highly reliable if they were either (i) selected by at least five methods, whatever the methods, (ii) or selected by both EN.mFDR and MIM (see Results). Then, we defined a highly reliable QTL as the interval of ± 3 cM around each highly reliable marker (Price 2006; Viana *et al.* 2016b), as predicted by polynomial local regression (loess) fitting of genetic positions to physical position. When several markers were selected inside the 6 cM interval, the QTL interval was extended accordingly. The genetic positions of this interval were then converted into physical positions, by fitting loess. QTLs overlapping for several traits on the SNP map were merged into a single QTL, by physical intervals’ union.

#### Candidate genes exploration:

After merging the most highly reliable QTLs colocalized between traits, we searched for underlying candidate genes. We retrieved the list of genes overlapping the intervals of our QTLs from the reference *Vitis* genome 12X.v2 and the VCost.v3 annotation (Canaguier *et al.* 2017). We then used the correspondence between IGGP (International Grapevine Genome Program) and NCBI RefSeq gene model identifiers provided by URGI (https://urgi.versailles.inra.fr/Species/Vitis/Annotations, last accessed on 07-21-21) to identify putative functions from NCBI, when available. For those genes with a putative function, we then refined the analysis to retrieve additional information about their function and expression. We searched UniProt (https://www.uniprot.org/, last accessed on 07-21-21) and TAIR (https://www.arabidopsis.org/, last accessed on 07-21-21) databases based on homologies to access a complete description of gene function, name, and corresponding locus in *Arabidopsis*. In addition, we used the GREAT (GRape Expression Atlas) RNA-seq data analysis workflow (https://great.colmar.inrae.fr/app/GREAT, last accessed on 07-21-21), which gathers published expression data, to assess the level of expression of our candidate genes in grapevine leaves and shoots, relevant organs for the traits under study. RNA-seq data are normalized as detailed in the ‘User manual’ section of the GREAT platform: “from the raw read counts, the normalized counts (library size normalization) and the RPKM (gene size normalization) are calculated for each gene in each sample.” Data were retrieved with all filters set to “Select All” except for the organ considered that was restricted to ‘Leaves’ and ‘Shoot’.

## Results

### Comparison of methods with simulated data prediction: cross-validation results

#### Traits with the same heritability value:

Methods were compared for prediction accuracy by applying cross-validation on simulated data with four different configurations and four heritability values.

**Figure 1:**
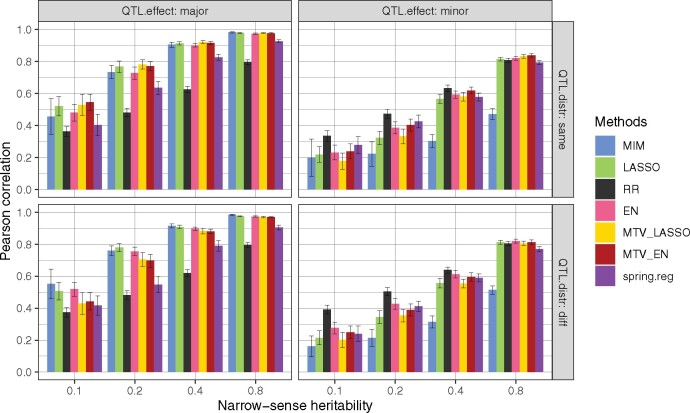
Genomic prediction accuracy (Pearson’s correlation between predicted and true genotypic values) of seven methods applied to 3961 markers and two simulated traits in a bi-parental population with different heritability values and four QTL configurations (number × distribution among traits). major: 2 QTLs; minor: 50 QTLs; same: QTLs at the same positions for both traits; diff: QTLs at different positions between traits. For each heritability value and configuration, prediction accuracy was averaged over 100 values (2 traits × 10 simulation replicates × 5 cross-validation folds). The error bar corresponds to the 95% confidence interval around the mean.

Mean Pearson’s correlation coefficient varied from 0.16 to 0.98, with a strong effect of heritability on prediction accuracy in all configurations, for the seven main methods (Figure 1). As expected, MIM performed very well in the “major” configurations across all heritability values but yielded the least accurate prediction in the “minor” ones. On the opposite, RR performed very well in the “minor” configurations, but yielded the least accurate prediction in the “major” ones. EN prediction performance was always intermediate between those of RR and LASSO. QTL distribution among traits – “same” (for QTLs at the same positions) or “diff” (for QTLs at different positions) - had very little effect on prediction accuracy. Moreover, we did not observe any superiority of multivariate methods over univariate ones, despite the strong genetic correlation simulated between traits (*ρ_B_*=0.8) and no correlation between errors.

The prediction accuracy of four additional methods is shown in Supplementary Figure S8 and prediction accuracy values. Other performance metrics (see Materials and Methods) are given for all methods in Supplementary Table S9. All IM methods yielded equivalent prediction accuracy. LASSO.GB did not improve performance compared to LASSO. MTV_RR showed equivalent performance to univariate RR. Prediction accuracy with *spring.dir.ols* was always lower than with *spring.reg*, and even very low for “minor” configurations. With 100 or 1000 simulated QTLs (under each QTL distribution) the ranking of methods based on prediction accuracy did not change compared to “minor” configurations (Supplementary Figure S10).

#### Traits with different heritability values:

To further compare prediction accuracy between univariate and multivariate methods, we simulated two correlated traits with different heritability values, 0.1 and 0.5. MTV_LASSO performed slightly better than univariate LASSO for the lowest heritability trait; however, differences were not significant (Supplementary Figure S11). On the opposite, prediction accuracy was lower with MTV_LASSO than with univariate LASSO for the highest heritability trait, reaching quite low values with 200 simulated QTLs. The same trends were also visible for MTV_EN and EN. MTV_RR never improved prediction over RR and *spring.reg* never performed better than RR.

Since these results were unexpected, we also compared prediction accuracy of the above methods using the simulated data published by Jia and Jannink (2012). We obtained very similar differences among methods to those with our own simulated data, even though prediction accuracy was higher in all cases (Supplementary Figure S12).

#### QTL detection: ROC curve results

**Figure 2:**
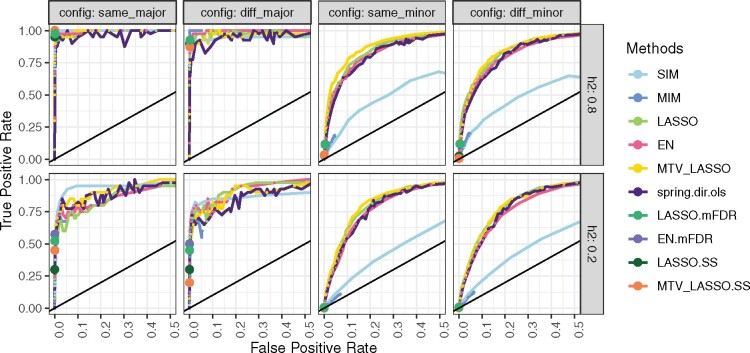
ROC curves for 10 methods applied to 3961 markers and two simulated traits in a bi-parental population with two heritability values and four QTL configurations (number × distribution among traits). major: 2 QTLs; minor: 50 QTLs; same: QTLs at the same positions for both traits; diff: QTLs at different positions between traits. Results are averaged over 2 traits × 10 simulation replicates. TPR: (number of correctly found QTLs/number of simulated QTLs), FPR: (number of falsely found QTLs/number of markers outside a QTL). For robust methods (mFDR and SS), as the FPR remained very low, we display only a single point corresponding to the lowest parameter constraint and thus to the highest TPR.

We compared the main methods mentioned above (except RR that does not perform marker selection), as well as some robust extensions, for their marker selection performance, by means of ROC curves, using the same simulated data in the four configurations (Figure 2). The closer a ROC curve obtained through a given method approaches the optimum point (*i.e.*, FPR = 0 and TPR = 1), the better is the method’s selection performance. As expected, IM methods (SIM and MIM) showed low selection performance when many minor QTLs were simulated and high selection performance when a few major QTLs were simulated. Note that the MIM curve was hardly visible; it roughly overlapped with the SIM curve but stopped at a low FPR because it could not select many QTLs by design.

The penalized regression methods always performed at least as well as the IM methods and even much better in the case of “minor” configurations. Among penalized methods, none was clearly better than the others in all configurations, except for a slight superiority of MTV_LASSO in the “same_minor” configuration. These methods, and particularly *spring.dir.ols*, displayed high variability in classification results with two simulated QTLs (“major” configurations). Indeed, when one QTL was not detected in one trait, impact on TPR was stronger than with 50 simulated QTLs.

The most interesting part of the ROC curve for QTL detection is the left most part, *i.e.*, that with a low FPR (*e.g.*, below 0.1). We thus calculated the partial Area Under the Curve (pAUC) for FPR between 0 and 0.1 for methods reaching that value (Supplementary Figure S13). EN resulted in constantly high pAUC across configurations and heritability values. In contrast, pAUC for SIM was quite high at low heritability values for the “same_major” configuration but dropped for other configurations and heritability values.

### Results on experimental data

#### Genomic predictive ability:

**Figure 3:**
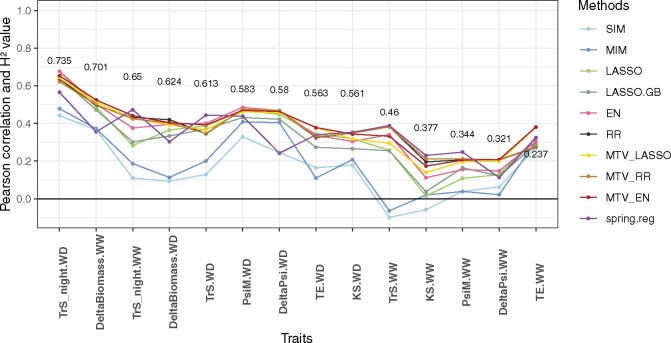
Mean genomic predictive ability (Pearson’s correlation between genotypic BLUPs and their predicted values), obtained by cross-validation for 10 methods applied to 14 traits related to water deficit and GBS gene-dose data, within a grapevine bi-parental population. Broad-sense heritability values are reported for each trait (y-position of the number corresponds to heritability estimate). Traits are ordered by decreasing heritability. For each trait, predictive ability is averaged over 10 cross-validation replicates × 5 cross-validation folds).

Mean genomic predictive ability per trait ranged from −0.10 to 0.68 (Figure 3 and Supplementary Table S14). It decreased with broad-sense heritability. IM methods (in blue) were always among the three poorest methods for prediction. Based on the mean predictive ability averaged across traits, MTV_EN yielded the highest correlation (0.384), followed by RR (0.3721), MTV_RR (0.3716), MTV_LASSO (0.369), EN (0.357), *spring.reg* (0.344), LASSO (0.329), LASSO.GB (0.313), MIM (0.200), and SIM (0.162). However, based on the number of traits for which each method gave the best prediction, *spring.reg* had the highest score, with 6 traits out of 14, followed by MTV_EN (3 out of 14) and EN (2 out of 14).

In a nutshell, MTV_EN and RR, tied with MTV_RR, provided the best mean predictive ability across traits. Even though *spring.reg* outperformed them for some traits, its performance was unstable and especially low for *DeltaBiomass.WW*, *DeltaBiomass.WD*, *DeltaPsi.WW*, and *DeltaPsi.WD*. For computational reasons, all traits could not be analyzed together with *spring.reg*, but were divided into three groups. These four traits with low predictive ability belonged to the same group. Yet, the effect of group membership on predictive ability was not significant at 5% (*P-value *=* *0.30 and percentage of variance explained = 24%).

#### QTL detection:

**Figure 4:**
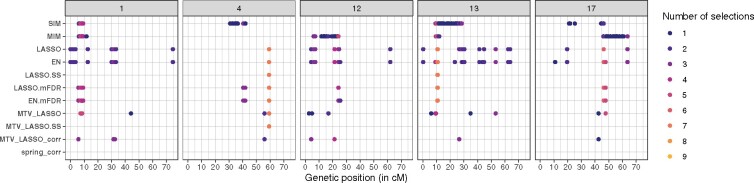
Marker selection by all methods for *TrS_night.WD* trait on chromosomes 1, 4, 12, 13, and 17. Each marker selected by a given method is represented by a colored point, the color indicating the number of methods that have selected that specific marker. The boxes correspond to chromosomes and the *x*-axis to the position along the genetic map (in cM).

To address the intersection of SNP selection by all methods, and determine the number of reliable intervals (QTLs) and their position, we examined in detail marker selection for each trait and chromosome. These results are given in Supplementary Table S15, together with genetic and physical positions and the percentage of variance explained. These results are plotted in Figure 4 for night-time transpiration under water deficit (*TrS_night.WD*) and in Supplementary Figure S16 for all traits.

Most of the time, more markers were selected for traits under water deficit than for traits in well-watered conditions, and they were most often selected by several methods. Penalized methods tended to select exactly the same markers, not only close ones; for example, for TrS_night.WD on chromosome 4, the same marker (at physical position 21,079,664 bp) was selected by seven different methods (Figure 4).

We considered markers selected by both MIM and EN.mFDR as highly reliable ones for three reasons: (1) markers selected by both MIM and EN were considered as reliable ones, because most markers selected by LASSO were also selected by EN, whereas MIM marker selection was quite different; (2) simulations showed that MIM and mFDR methods led to a very low FPR; (3) these methods belong to different method classes (IM *vs* penalized regression). We also considered as highly reliable those markers selected by at least five methods. These criteria resulted in a set of 59 highly reliable selected markers, which were converted to genetic intervals of ± 3 cM around each selected marker. Overlapping intervals per trait were merged, resulting in 25 highly reliable QTLs.

These 25 QTLs involved nine traits, mostly under water deficit, and were located on seven chromosomes (Supplementary Figure S17, Supplementary Table S18). Some QTLs colocalized for different traits, such as on chromosome 1, and had similar distributions of genotypic BLUPs according to genotypic classes (Supplementary Figure S19).

Among these 25 QTLs, we found eight new highly reliable QTLs compared to Coupel-Ledru *et al.* (2014, 2016), among which five were not detected by MIM. In particular, a completely new QTL for *TrS_night.WD* was found alone on chromosome 12. Most other new QTLs were colocalized with QTLs previously found in single-year analysis and/or for the other watering condition. Notably, we observed colocalization of *TrS_night.WD*, *TE.WD* and *DeltaBiomass.WD* QTLs on chromosomes 4 and 17.

In total, the percentage of variance explained (adjusted *R*^2^) per trait was 51.3% for *TrS_night.WD* (36% in 2012 for Coupel-Ledru *et al.* 2016), 33.9% for *PsiM.WD*, 31.4% for *DeltaPsi.WD*, 26.9% for *DeltaBiomass.WW*, 19.4% for *TE.WD*, 18.6% for *TE.WW*, 17.0% for *KS.WD*, 14.9% for *DeltaBiomass.WD*, and 8.5% for *TrS.WD*.

### Candidate genes

**Figure 5:**
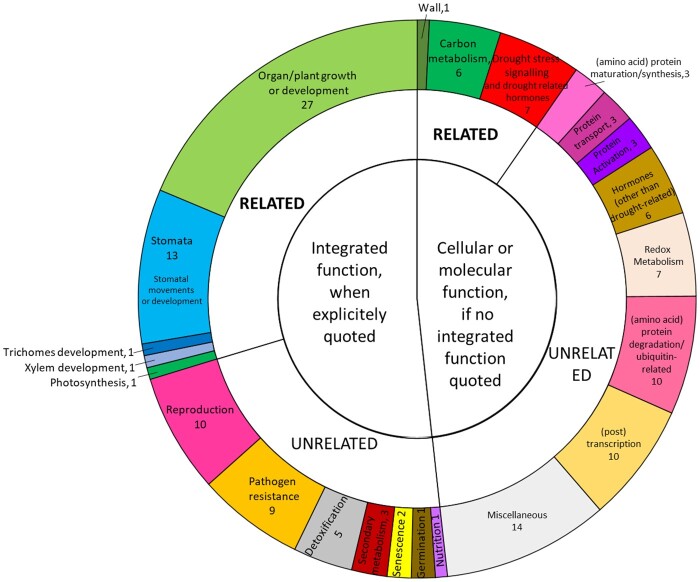
Functional classification of the annotated genes underlying the highly reliable QTL detected on chromosome 4 for night-time transpiration, growth, and TE. Hierarchical classification of the 161 genes based on their functions. See Supplementary Table S21 for the details of this classification. When an integrated function at the organ or plant level was explicitly quoted in the gene annotation, genes were classified on this basis. When no integrated function was explicitly quoted, they were classified based on their cellular or molecular function. In both cases, functions were then classified as “Related” if related to the traits of interest in this QTL, or “Unrelated” if not.

After merging the QTLs colocalized between traits, we obtained 12 intervals, located on chromosomes 1, 4, 10, 12, 13, 17, and 18, harboring a total of 3461 genes according to the VCost.v3 annotation (Canaguier *et al.* 2017). Among them, 2379 had an NCBI Refseq identifier and 1757 a putative function (Supplementary Table S20). We then focused our analysis on the eight “new” intervals, *i.e.*, those that were not overlapping with QTL intervals repeated over years by Coupel-Ledru *et al.* (2014, 2016). They encompassed 1155 genes, half of which were annotated. We were able to retrieve from TAIR and/or UniProt a more precise description of the gene function for 86% of the annotated genes (Supplementary Table S20). The remaining ones either did not have any homologous gene in *Arabidopsis* or were not described in the above-mentioned databases. RNA-seq data was available on the GREAT platform for 90% of the annotated genes. We further focused on the highly reliable QTL co-localized on chromosome 4 for *TE*, *TrS_night* and *DeltaBiomass* under various conditions. We proceeded to a functional classification of the 161 annotated genes underlying this QTL, based on the full description previously retrieved (Supplementary Tables S5 and S21). For 75 genes, an integrated function at the plant or organ level was explicitly quoted in the homologous-based description. We grouped these integrated functions into 12 major groups (Figure 5). For a substantial part of genes, functions consistently related to traits involved: 15 genes related to hydraulics (stomata, xylem, and trichomes), relevant for *TrS_night* and thus *TE*; 27 to growth or development and one to photosynthesis, both relevant to *DeltaBiomass* and thus *TE*. For the 86 genes for which no integrated function was explicitly quoted, we further built a classification based on their cellular or molecular function. Among them, we found six genes related to carbon metabolism, one to wall formation (both relevant for *DeltaBiomass*) and six to drought stress signaling and drought-related hormones (relevant for *TrS_night*).

## Discussion

This study contributes to our knowledge of the complex genetic determinism of vegetative traits under different watering conditions in three different ways. We compared by simulation several univariate and multivariate methods for genomic prediction and QTL detection, and re-analyzed grapevine phenotypes obtained under semi-controlled conditions. In particular, we showed that penalized methods are valuable not only for prediction but also for QTL detection. Indeed, we found new QTLs using these methods and identified relevant candidate genes.

### Methodological aspects: method comparison

#### Handling linkage disequilibrium:

IM methods estimate genotypic probabilities between markers according to a genetic map which is computationally costly to build. On the other hand, most penalized methods do not require any previous knowledge on LD.

The LASSO assumption that all predictor variables are independent is all the more violated that there are many markers. In the case of a group of correlated predictors (*e.g.*, SNPs in LD), EN selects either no or all predictors within the group with close estimated effects (Zou and Hastie 2005), whereas LASSO selects a single predictor. In that sense, EN aims at correcting the drawbacks of LASSO when predictor variables are highly correlated. By exploring a large number of configurations of the finite-sample high-dimensional regression problem, Wang *et al.* (2020) showed that EN is competitive for both prediction and selection in most cases with highly correlated predictors. In agreement with these results, we showed that EN performed well for both prediction and selection on our simulated data, and that multivariate EN performed best for prediction on grapevine experimental data.

It would be interesting to test whether EN still remains the main default method when applied to a population with a shorter LD, *e.g.*, a diversity panel as defined in Nicolas *et al.* (2016). Indeed, the ranking of methods is likely to depend not only on linkage disequilibrium and population size, but also on the genetic architecture of the traits of interest as well as the accuracy with which phenotypic values were obtained, and all these variables can interact with each other, but studying this was out of the scope of the current work.

#### Comparison between interval-mapping and penalized regression methods for genomic prediction:

As expected, IM methods performed poorly to predict accurate genotypic values when the QTL number was large. These conclusions are in agreement with previous studies (Figure 1 and Supplementary Figure S8), even though most implemented marker selection methods other than interval-mapping (Bernardo and Yu 2007; Lorenzana and Bernardo 2009; Mayor and Bernardo 2009; Olatoye *et al.* 2019). This confirms that for complex traits, genomic prediction should not be based only on QTLs detected by IM methods.

None of the penalized univariate methods performed optimally in all cases (Figures 1 and 3 and Supplementary Figure S8), as also found in the literature (Heslot *et al.* 2012; Riedelsheimer *et al.* 2012; Azodi *et al.* 2019). As shown by simulation, RR was better adapted to highly polygenic genetic architecture, whereas LASSO was better adapted to a few major QTLs. Moreover, in the case of many minor QTLs, RR was the most stable method across heritability values, as previously described for several traits and species (Heslot *et al.* 2012; Azodi *et al.* 2019). However, RR prediction accuracy dropped when the QTL number was too small, whereas EN still predicted as well as LASSO. EN was hence well adapted to various numbers and distributions of QTLs.

#### Multivariate vs univariate:

When the same heritability was simulated for both trait variables, no superiority of multivariate methods was observed, even when both traits had QTLs at the same positions (Figure 1 and Supplementary Figure S8). When different heritability values were simulated for the two traits, we observed a slight superiority of MTV_LASSO (resp. MTV_EN) over LASSO (resp. EN) only in the “same” and “major” configuration (with both traits sharing the same two QTLs) for the trait with small heritability (Supplementary Figure S11).

Other authors who tested multivariate GP on simulated data systematically applied different heritability values; they found a superiority of multivariate methods over univariate ones for the trait with the smallest heritability (Calus and Veerkamp 2011; Guo *et al.* 2014; Jiang *et al.* 2015; Dagnachew and Meuwissen 2019). However, all these studies were based on a smaller, more favorable, *p*/*n* ratio, a key component of high-dimensional models (Verzelen 2012). For example, in Jia and Jannink (2012), their 500 observations for 2020 predictors correspond to a ratio of ∼4, compared to our 188 observations for 3961 predictors corresponding to a ratio of ∼21. Indeed, parameters *n* and *p* are involved in the sample complexity function defined in Obozinski *et al.* (2011), which predicts the theoretical cases where MTV_LASSO is superior to its univariate counterpart in terms of variable selection. Accordingly, applying our methods to Jia and Jannink (2012) data allowed us to evidence a larger difference between univariate and multivariate LASSO than with our simulated data.

Unexpectedly, when reanalyzing the data simulated by Jia and Jannink (2012), we obtained lower prediction accuracy with our MTV_LASSO (Supplementary Figure S11) than they did with their multivariate BayesA (their Figure 1A). A similar result in a univariate setting was found by Guan and Stephens (2011), who compared BSVR (comparable to BayesA) and the LASSO. They found that BSVR had markedly higher power than LASSO. Moreover, the parameters of both BSVR [in Guan and Stephens (2011)] and BayesA [in Jia and Jannink (2012)] were estimated with a MCMC algorithm. No inner cross-validation was needed, hence the sample used to train the model was larger. This difference may explain why Figure 1A from Jia and Jannink (2012) shows better prediction accuracies for multi-trait models compared to their single-trait counterparts, although no confidence interval was displayed. Note that our RR prediction accuracies were close to those of their GBLUP (univariate and multivariate). In conclusion, prediction accuracy is affected both by the dimension of the problem (*i.e.*, *n* and *p*) and the method used (*i.e.*, Bayesian with MCMC or cross-validation).

For experimental data, we observed that MTV_LASSO (respectively MTV_EN) was superior to LASSO (resp. EN) for the five traits with the smallest heritability (Figure 3). Such this improvement suggests that MTV_LASSO (resp. MTV_EN) was able to borrow signals from the most heritable traits to the least heritable ones, likely because of a partially overlapping genetic architecture between these traits. This interpretation is reinforced by the fact that a QTL for low-H2 trait, *TE.WW*, colocalizes on chromosome 4 with QTLs for four high-to-moderate-H2 traits (*TrS_night.WD*, *DeltaBiomass.WW*, *DeltaBiomass.WD* and *TE.WD*). This improvement was not found in Jia and Jannink (2012), who also tested their methods on real pine data from Resende *et al.* (2012). These observations suggest that the number of traits analyzed (14 in our case and 2 in Jia and Jannink 2012 study) may also play a role in the gain in prediction accuracy of multivariate over univariate methods.

Furthermore, we simulated data with various levels of residual correlation among traits (0, 0.4, and 1) but this did not significantly change prediction results (data not shown). A more detailed methodological analysis is out of the scope of the current work.

#### Comparison between interval-mapping and penalized regression methods for QTL detection:

Comparison with the ROC curve between IM and penalized regression methods for marker selection has not been extensively studied before. As expected, we found that IM methods are well adapted to detect a few major QTLs but not many minor QTLs (Figure 2). Yi *et al.* (2015) similarly compared the FDR and TPR reached by single marker analysis and different penalized regression methods, some of which being adapted to control FDR; they found contrasting results, depending on the criteria studied (modified version of TPR or FDR). However, they focused only on an association panel whereas we worked on a bi-parental population. Other authors (Cho *et al.* 2010; Li and Sillanpää 2012; Waldmann *et al.* 2013) successfully applied LASSO or EN for performing GWAS, but without comparing IM and penalized methods for QTL detection. Moreover, we found that penalized methods could be as good at marker selection as IM methods, and even far better when there were many minor QTLs. Among the penalized methods we compared, none clearly outperformed the others for marker selection in all configurations.

##### Multivariate vs univariate:

As MTV_LASSO selects one predictor for all traits, its superiority over univariate LASSO depends on QTL distribution across traits, notably on the amount of genetic basis shared by the traits (Obozinski *et al.* 2011). However, as for prediction, we showed that MTV_LASSO performance was not different whether QTLs were at the same or at different positions across traits (Figure 2). Nevertheless, we observed that MTV_LASSO was slightly better than LASSO when many QTLs were simulated.

SPRING had never been evaluated before for its quality of predictor selection. As for prediction, SPRING showed unstable results across our simulation replicates and hyper-parameter values. However, for the ROC curve, we did not include predictor structure in the model, which may have hampered marker selection quality.

#### Efficient default method for both QTL detection and genomic prediction:

IM methods were designed for marker selection; hence they are not expected to be optimal for prediction, as confirmed in our study. Among penalized regression methods, some may be better at prediction than at marker selection, and vice versa. For example, our results showed that EN performed well across several configurations for both aims. Some methods such as SPRING are specially adapted to handle both purposes but this method produced too variable results for either prediction or QTL detection. However, SPRING is a recent method that still can be improved to correct this drawback.

New penalized regression methods are continuously being developed. In particular, graph structured sparse subset selection (Grass) recently proved to outperform existing methods for both prediction and predictor selection, thanks to a *L*0 regularization that limits the number of nonzero coefficients in the model (Do *et al.* 2020). It could be tested on our data when available. Moreover, multivariate methods are presented as being more efficient at using the whole signal in the data, whether for marker selection (Inouye *et al.* 2012) or prediction (Jia and Jannink 2012; Guo *et al.* 2014), but our results revealed no systematic advantage of multivariate methods over univariate ones for both aims.

Using penalized methods for both marker selection and genomic prediction requires adapted hyper-parameter values. For EN, LASSO, and SPRING, the *λ* value controls sparsity (*e.g.*, the number of selected markers). Thus, the optimal *λ* value might not be the same if the aim is to limit FPR or to maximize predictive ability (Li and Sillanpää 2012). For prediction, we traditionally use cross-validation to tune hyper-parameters by minimizing MSE. For marker selection, there is no direct equivalence. That is why we tested extensions of these methods (mFDR and SS) that control sparsity for robust marker selection; both proved efficient in selecting the most relevant markers.

In order to shed light on the link between prediction accuracy and marker selection, we plotted the prediction accuracy at each point of the ROC curve for EN and EN.mFDR against FPR for minor configurations (with 50 simulated QTLs) (Supplementary Figure S22). For EN, we showed that prediction accuracy reached its maximum when FPR was below 0.05. Then, FPR increased while prediction accuracy decreased until it reached a plateau. This means that prediction quality is intimately linked to selection quality, especially at low heritability. For EN.mFDR, FPR always stayed below 0.015 but prediction accuracy was lower.

As a consequence, as an efficient default method, we advise at this stage to apply EN for performing genomic prediction, and its extension EN.mFDR for performing sparser marker selection.

#### Genetic determinism and prediction of grapevine response to water deficit:

Based on experimental data on the Syrah × Grenache progeny (new genotypic data and already published phenotypic data), we compared the same methods as above for both prediction and marker selection. To the best of our knowledge, grapevine GP within a bi-parental family has only been applied to a limited number of traits, with very few methods and never multivariate GP. Fodor *et al.* (2014) studied GP in grapevine with simulated data on a diverse structured population; they tested RR-BLUP, Bayesian Lasso, and a combination of marker selection and RR. Viana *et al.* (2016a) used an inter-specific grapevine bi-parental population. They predicted cluster and berry phenotypes (number and length of clusters, number, and weight of berries, juice pH, titrable acidity) with RR-BLUP and Bayesian LASSO applied to table grape breeding. In addition to yielding further insights into method comparison beyond those obtained by simulation, our study brought valuable novel biological knowledge about grapevine water use under different watering conditions. Indeed, new methods and the new SNP genetic map allowed us to find novel QTLs, as compared to those previously detected with the same phenotypic data (Coupel-Ledru *et al.* 2014, 2016). Our study also provides novel results of practical interest to grapevine breeders. We showed what predictive ability they can expect for drought-related traits within a progeny: here, always higher than 0.3, and up to more than 0.65 for some traits. Even though these traits are difficult to phenotype, they correspond to crucial breeding targets in the context of climate change. Our results may help motivate their phenotyping in the training panels of breeding programs.

#### Predictive ability and genetic architecture:

Among univariate penalized methods, RR generally had equivalent or better predictive ability compared to LASSO. For the traits with the largest discrepancy between RR and LASSO, this suggests that trait variability was rather due to many minor QTLs than to a few major ones. On the other hand, for a few traits, *e.g.*, *PsiM.WD*, *DeltaPsi.WD*, and *TE.WW*, predictive abilities with sparse methods (*s*, LASSO, and IM methods) were better than with RR, suggesting a genetic architecture with few major QTLs rather than an infinitesimal one in those cases.

Finally, while not considered by the penalized methods used, nonadditive genetic effects such as epistasis could be involved. We, therefore, tested the superiority of LASSO.GB over LASSO. Extreme Gradient Boosting methods are indeed among the best machine learning methods (Chen and Guestrin 2016). LASSO.GB did not markedly increase predictive ability on experimental data (Figure 3). However, we cannot exclude that this might be due to a poor optimization of Extreme Gradient Boosting parameters or to an insufficient number of observations to correctly fit the model. We also tested if coding differently the design matrix to estimate dominance genetic effects improved predictive ability and it was not the case (data not shown).

#### Candidate gene analysis:

The thorough methodology deployed for candidate genes analysis allowed us not only to retrieve a list of genes potentially underlying the QTLs of interest, but also to classify them based on their function and expression to point at the most likely candidates. We focused on the highly reliable QTL detected on chromosome 4 for *TrS_night*, *TE*, and *DeltaBiomass*. *TrS_night* QTL was previously described as a promising target for marker-assisted selection, as alleles limiting night-time transpiration also favor plant growth, resulting in a doubly, beneficial impact on improving TE (Coupel-Ledru *et al.* 2016). Moreover, this QTL was found using seven methods. Among the plethora of integrated functions represented within the list of annotated genes underlying this QTL, we show here that a subset of more likely candidates can be defined as possibly related to the traits of interest. On the one hand, these include genes related to broad-sense hydraulics and water loss, with a possible direct impact on *TrS_night*: seven genes involved in stomatal development, nine involved in stomatal opening—sometimes through the abscicic acid signaling pathway—, one related to xylem development and one to trichome development (Supplementary Table S21). One of these genes, the trihelix transcription factor GT-2 (*Vitvi04g01604*), was specifically shown to impact transpiration and TE in *Arabidopsis* by acting as a negative regulator of stomatal density. On the other hand, 27 genes in the list are directly related to growth, development, or photosynthesis, hinting to a possible direct impact on *DeltaBiomass*. A histidine kinase 1 (*Vitvi04g01483*) may be a particularly interesting candidate for its multiple roles in *Arabidopsis* in ABA signaling, stomatal development, and plant growth, hence potentially simultaneously acting on both components of *TE*. Both these likely candidates were often highly expressed in grapevine leaves. More precise analyses of these candidate genes, including functional genomics work and possibly gene editing, will now be necessary to identify the causative polymorphisms under these new QTLs.

## Conclusions

Rather than decoupling genomic prediction from the identification of major QTLs, we argue for the need to pursue both goals jointly. Indeed, they provide complementary information on the genetic architecture of the target traits, as well the key underlying functions. Our study provided encouraging findings for further implementing genomic prediction in grapevine breeding programs. Applied to both simulated and 14 experimental traits, univariate and multivariate Elastic Net proved to be efficient for both goals, followed by mFDR control for the robust identification of QTLs. Moreover, of interest to plant biologists seeking to understand the response to water stress, our results highlighted several candidate genes underlying integrated traits such as night-time transpiration, TE, and biomass production. For some, their putative functions suggest causal links with stomatal functioning, trichome development, or the ABA pathway.

## Code availability

All software we used was free and open-source and most analyses were done with R (R Core Team 2020), notably graphs that were created using the ggplot2 package (Wickham 2016), version 3.3.2. All R scripts used for analysis, *i.e.* genetic mapping, simulation, phenotypic analysis, prediction, and QTL detection, are available online at https://doi.org/10.15454/NOUQY2. Many of the custom functions we used are available in a package for reproducibility purposes, R/rutilstimflutre (Flutre 2019).

## Author contribution statement

T.F., A.D., and L.L.C. conceived the idea of the study and contributed to funding acquisition; A.C.L. and T.S. obtained the phenotypic data used in this work; C.B. and A.D. carried out the analysis of the genotypic data, with input data previously analyzed by T.F.; T.F. and J.C. performed preliminary multivariate genomic prediction analysis; A.C.L. and T.S. interpreted the results from the candidate gene analysis and wrote the corresponding parts in the manuscript; PT is the PhD supervisor of CB; CB wrote the original draft, which was reviewed and edited by all authors. All authors read and approved the final manuscript.

## Ethical standards

The authors declare that the experiments comply with the current laws of the country in which they were carried out.

## Supplementary Material

jkab248_Supplementary_Data

## Data Availability

Genotypic data, as well as the genetic map, are available online at https://doi.org/10.15454/QEDX2V. Raw phenotypic data are available in a second online repository at https://doi.org/10.15454/YTRKV6. Supplementary material is available at *G3* online.
