# Supplementary material for: Harnessing multivariate, penalized regression methods for genomic prediction and QTL detection of drought-related traits in grapevine
Source: G3 (Bethesda). 2021 Jul 22;11(9):jkab248. doi: 10.1093/g3journal/jkab248 (PMC8496232; doi:10.1093/g3journal/jkab248)
Supplement: jkab248_Supplementary_Data [file jkab248_supplementary_data.zip › jkab248-suppl_data/GENETICS-G3-2021-402101-s01.zip!/Fig5.pdf]

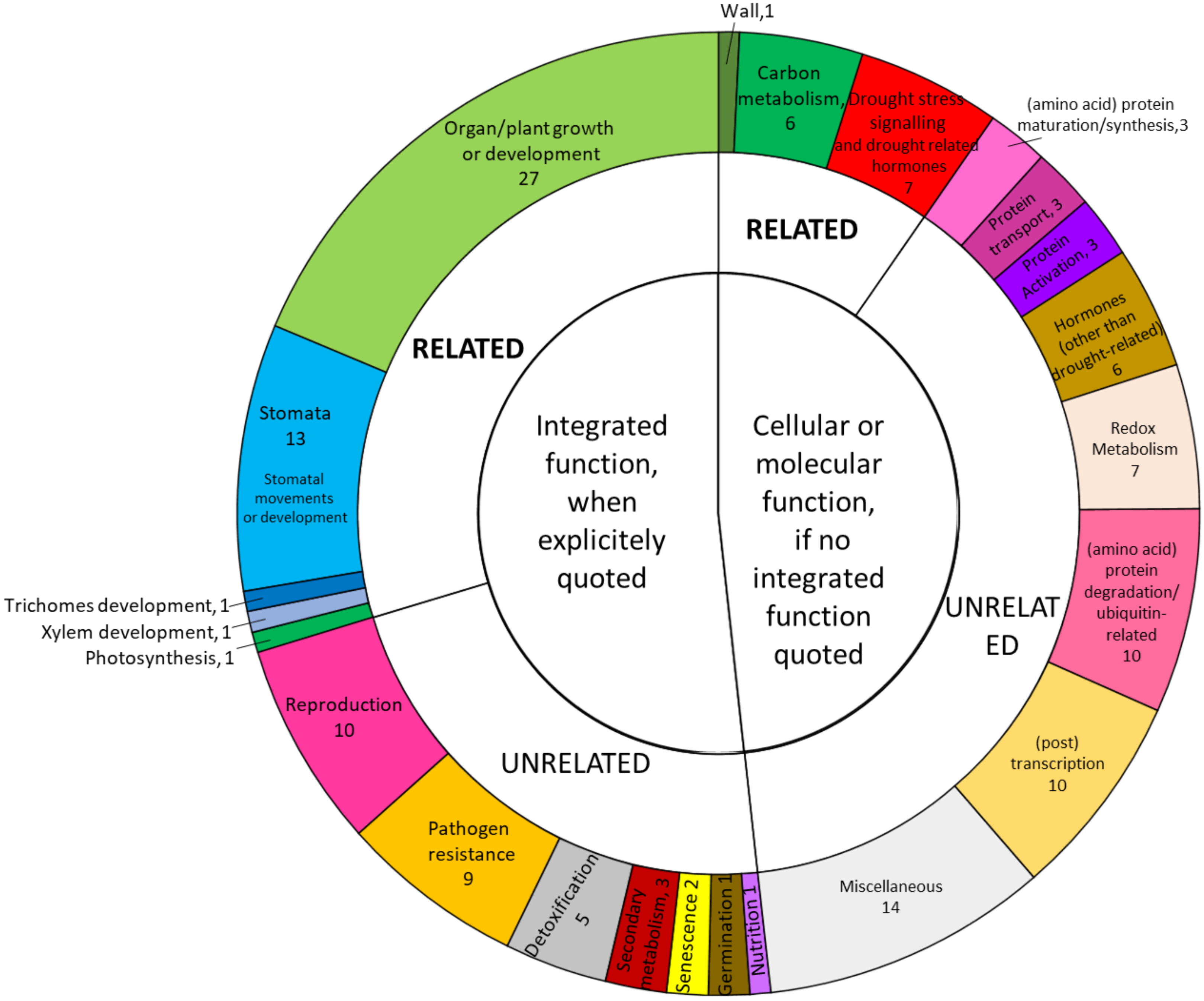

Wall,1
Carbon metabolism, 6
Drought stress signalling and drought related hormones 7
(amino acid) protein maturation/synthesis,3
Protein transport, 3
Protein Activation, 3
Hormones (other than drought-related) 6
Redox Metabolism 7
(amino acid) protein degradation/ ubiquitin-related 10
(post) transcription 10
Miscellaneous 14
Nutrition 1
Germination 1
Senescence 2
Secondary metabolism, 3
Detoxification 5
Pathogen resistance 9
Reproduction 10
Photosynthesis, 1
Xylem development, 1
Trichomes development, 1
Stomata 13
Stomatal movements or development
Organ/plant growth or development 27
RELATED
RELATED
UNRELATED
UNRELATED
Integrated function, when explicitely quoted
Cellular or molecular function, if no integrated function quoted
