# Supplementary material for: Harnessing multivariate, penalized regression methods for genomic prediction and QTL detection of drought-related traits in grapevine
Source: G3 (Bethesda). 2021 Jul 22;11(9):jkab248. doi: 10.1093/g3journal/jkab248 (PMC8496232; doi:10.1093/g3journal/jkab248)
Supplement: jkab248_Supplementary_Data [file jkab248_supplementary_data.zip › jkab248-suppl_data/GENETICS-G3-2021-402101-s01.zip!/G3Logo_Horiz.pdf]

G3 Genes | Genomes | Genetics
