# Supplementary material for: Harnessing multivariate, penalized regression methods for genomic prediction and QTL detection of drought-related traits in grapevine
Source: G3 (Bethesda). 2021 Jul 22;11(9):jkab248. doi: 10.1093/g3journal/jkab248 (PMC8496232; doi:10.1093/g3journal/jkab248)
Supplement: jkab248_Supplementary_Data [file jkab248_supplementary_data.zip › jkab248-suppl_data/GENETICS-G3-2021-402101-s02.zip!/supp/FigureS16_selection_all-resp.pdf]

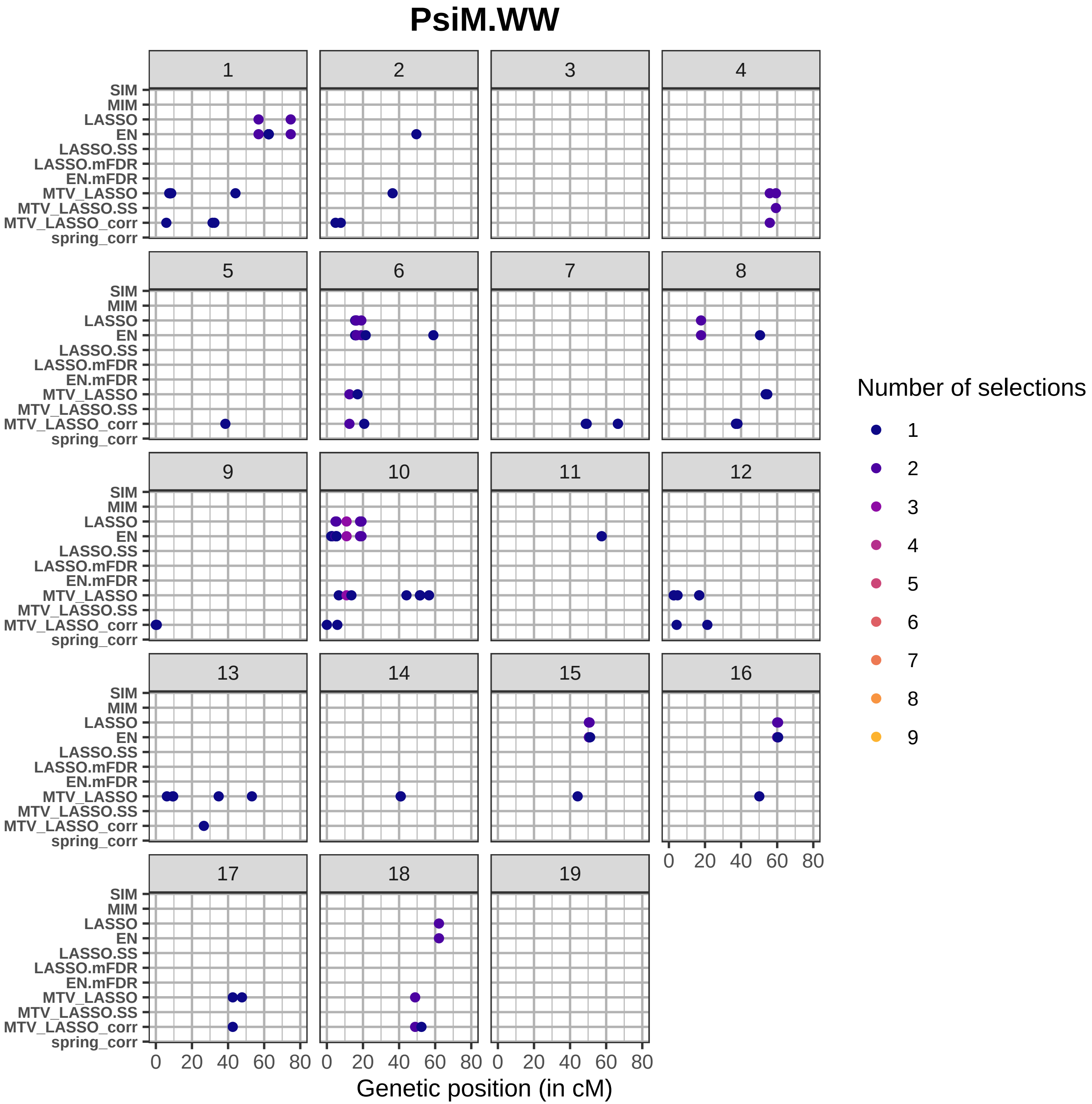

PsiM.WW
1
2
3
4
5
6
7
8
9
10
11
12
13
14
15
16
17
18
19
SIM
MIM
LASSO
EN
LASSO.SS
LASSO.mFDR
EN.mFDR
MTV_LASSO
MTV_LASSO.SS
MTV_LASSO_corr
spring_corr
0
20
40
60
80
Genetic position (in cM)
Number of selections
1
2
3
4
5
6
7
8
9

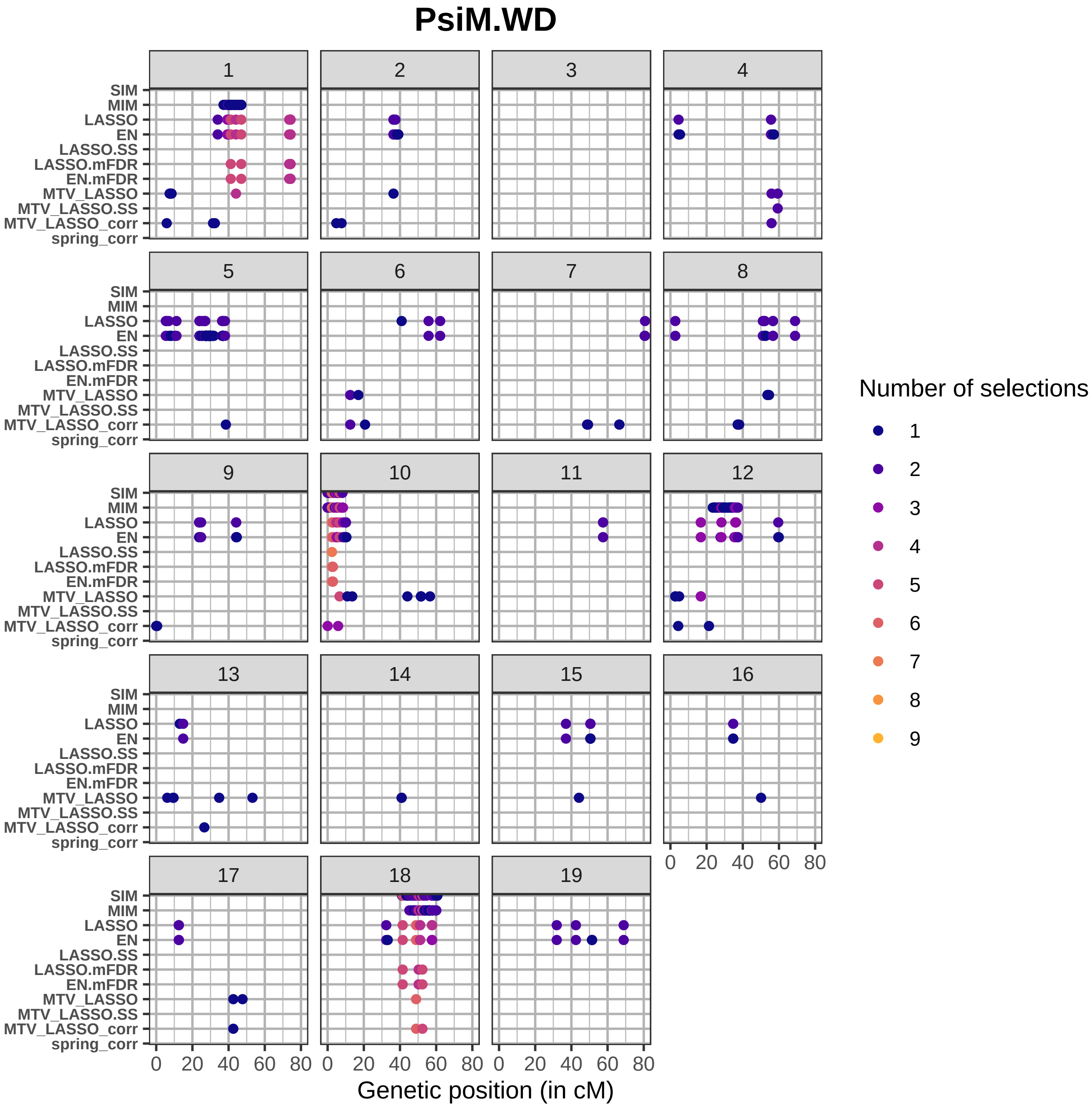
PsiM.WD
1
2
3
4
5
6
7
8
9
10
11
12
13
14
15
16
17
18
19
SIM
MIM
LASSO
EN
LASSO.SS
LASSO.mFDR
EN.mFDR
MTV_LASSO
MTV_LASSO.SS
MTV_LASSO_corr
spring_corr
0 20 40 60 80
Genetic position (in cM)
Number of selections
1
2
3
4
5
6
7
8
9

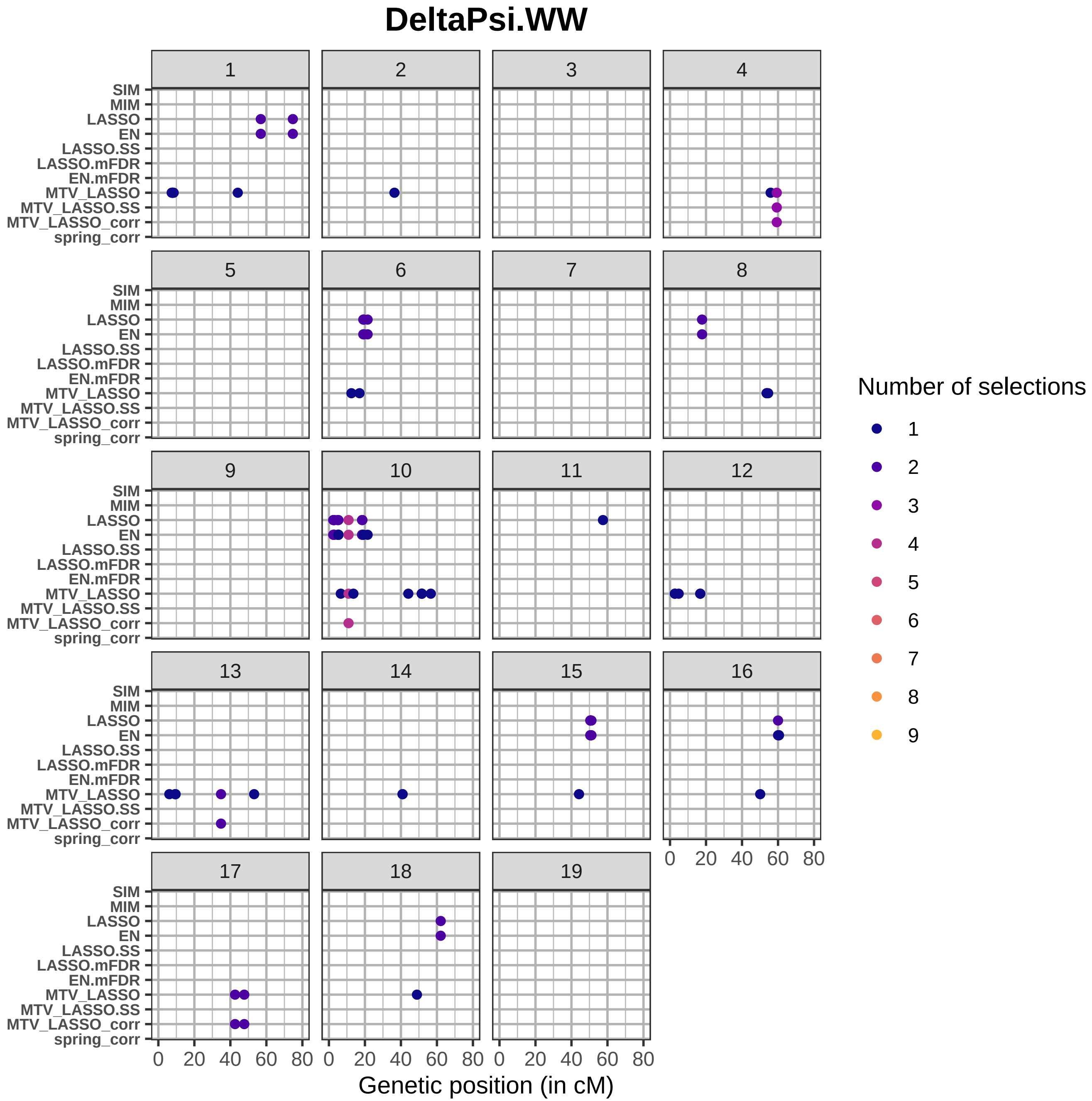
DeltaPsi.WW
SIM
MIM
LASSO
EN
LASSO.SS
LASSO.mFDR
EN.mFDR
MTV_LASSO
MTV_LASSO.SS
MTV_LASSO_corr
spring_corr
1
2
3
4
5
6
7
8
9
10
11
12
13
14
15
16
17
18
19
0
20
40
60
80
Genetic position (in cM)
Number of selections
1
2
3
4
5
6
7
8
9

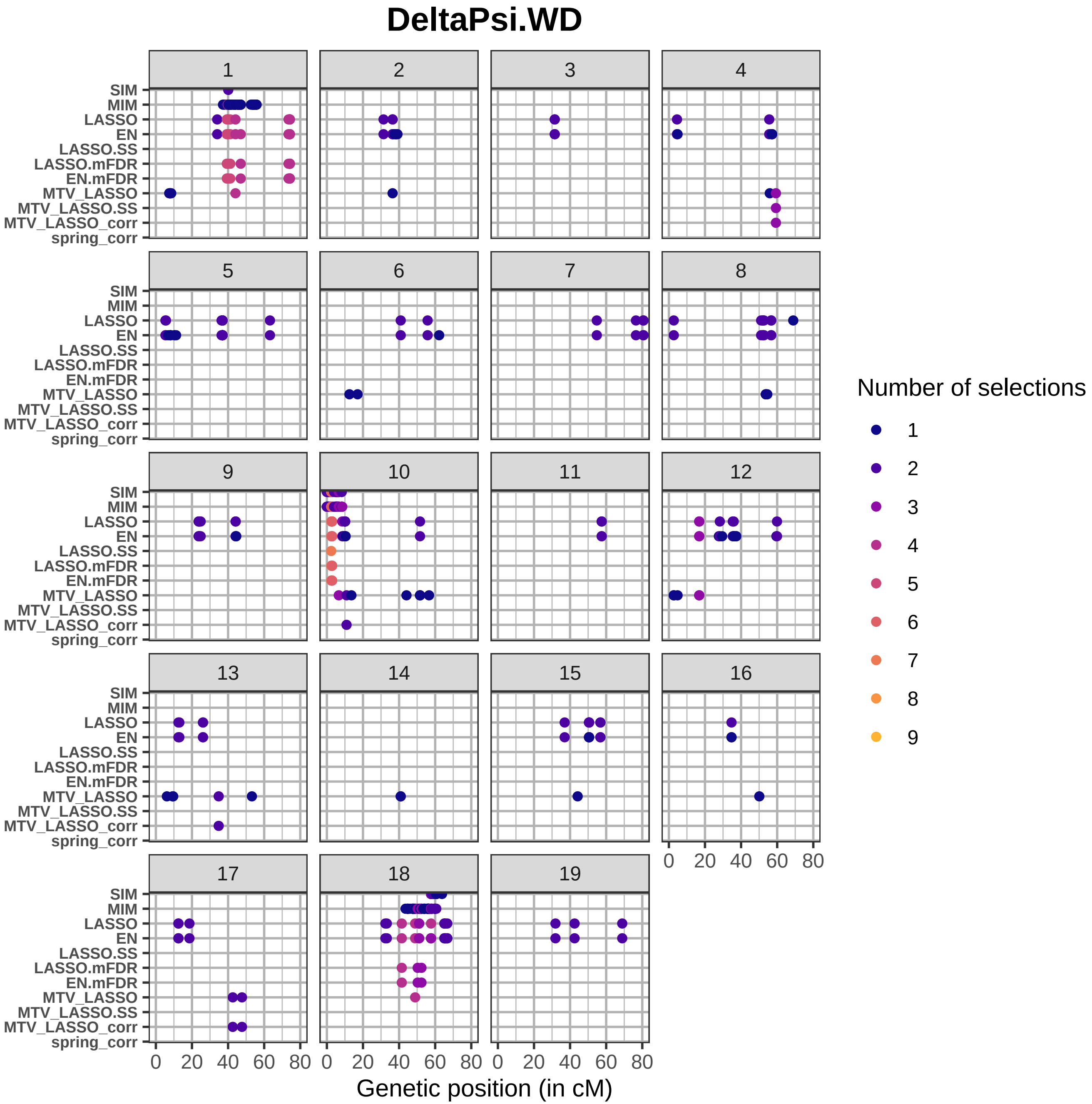
DeltaPsi.WD
1
2
3
4
5
6
7
8
9
10
11
12
13
14
15
16
17
18
19
SIM
MIM
LASSO
EN
LASSO.SS
LASSO.mFDR
EN.mFDR
MTV_LASSO
MTV_LASSO.SS
MTV_LASSO_corr
spring_corr
0 20 40 60 80
Genetic position (in cM)
Number of selections
1
2
3
4
5
6
7
8
9

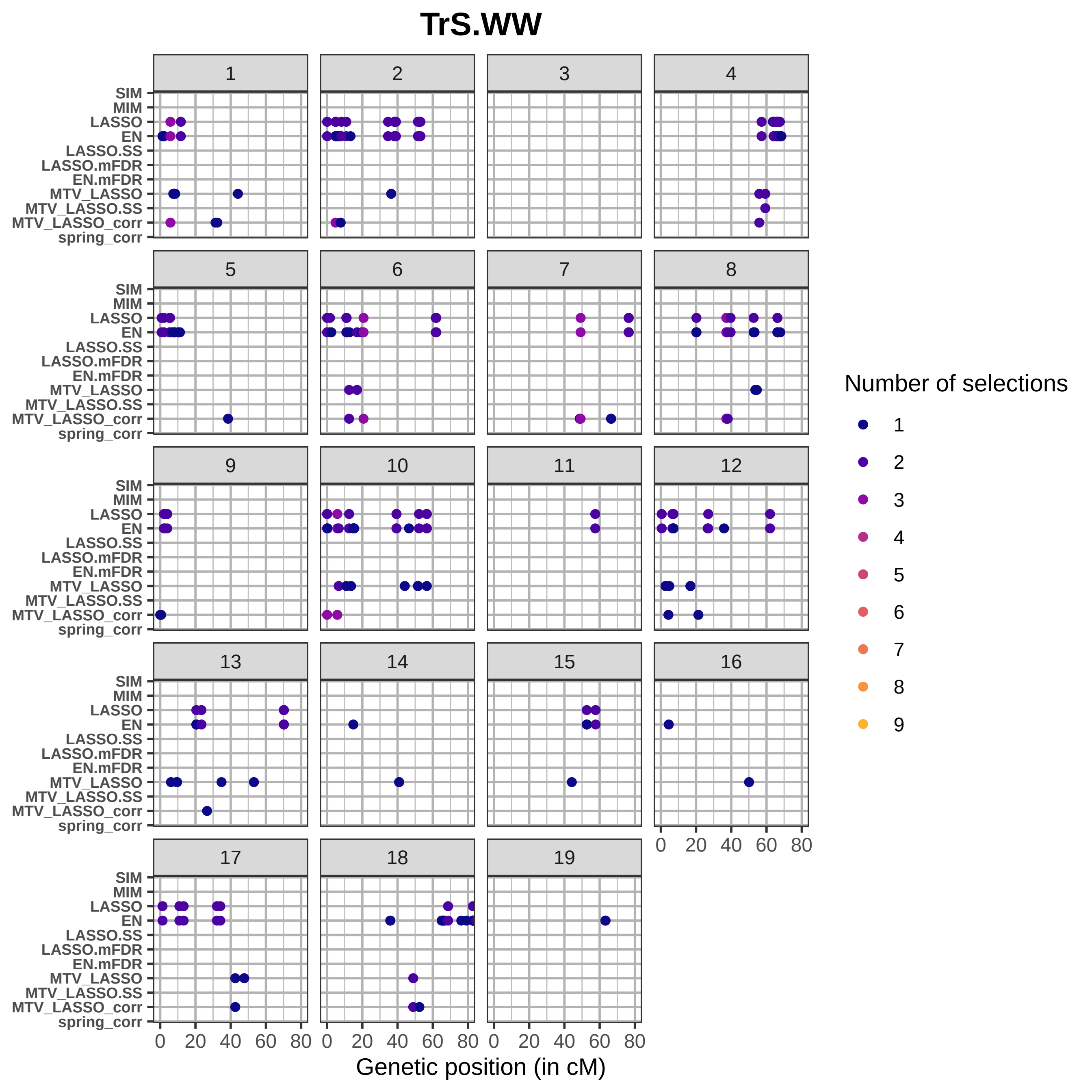
TrS.WW
1
2
3
4
5
6
7
8
9
10
11
12
13
14
15
16
17
18
19
SIM
MIM
LASSO
EN
LASSO.SS
LASSO.mFDR
EN.mFDR
MTV_LASSO
MTV_LASSO.SS
MTV_LASSO_corr
spring_corr
0 20 40 60 80
Genetic position (in cM)
Number of selections
1
2
3
4
5
6
7
8
9

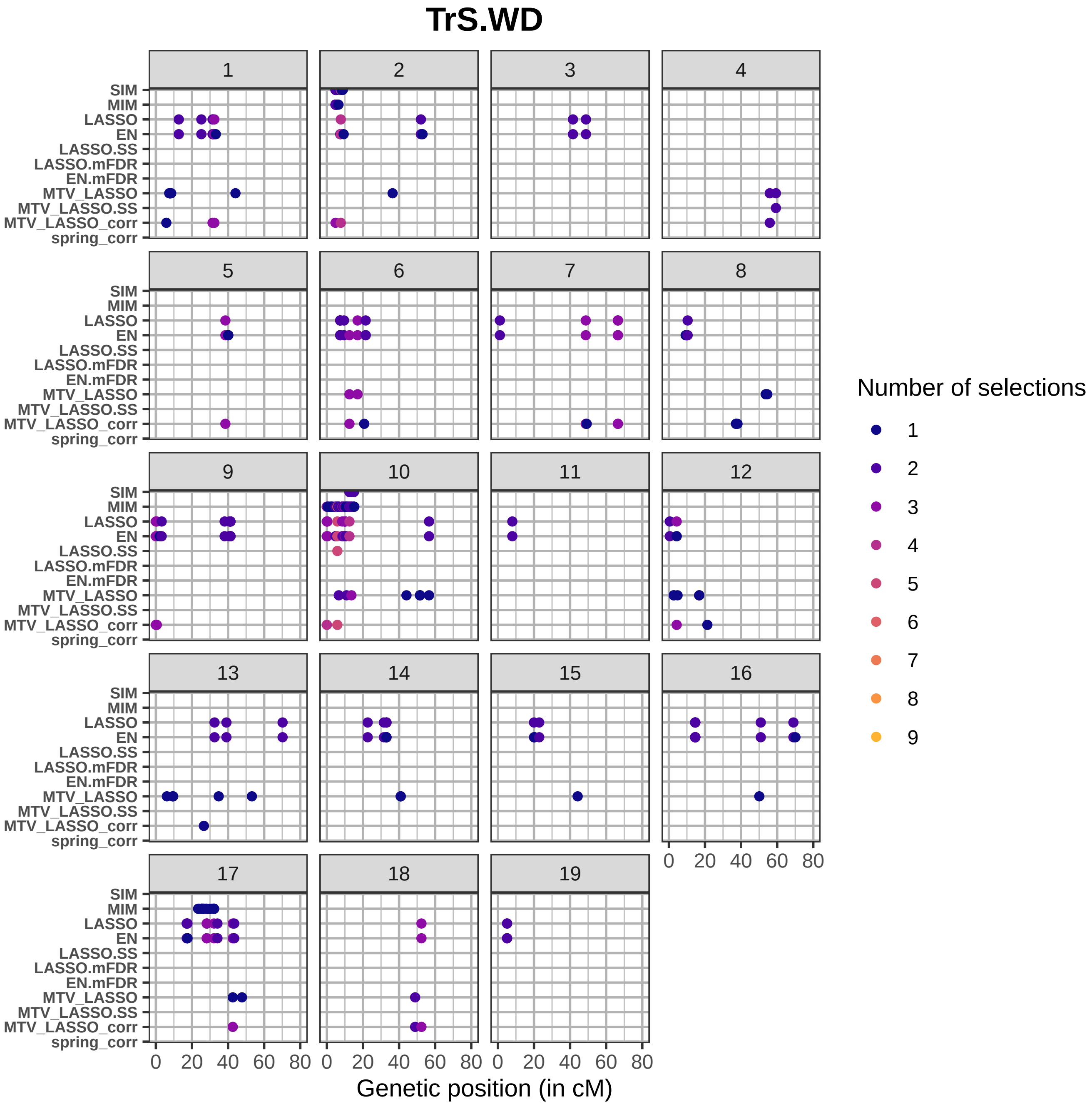
TrS.WD
1
2
3
4
5
6
7
8
9
10
11
12
13
14
15
16
17
18
19
SIM
MIM
LASSO
EN
LASSO.SS
LASSO.mFDR
EN.mFDR
MTV_LASSO
MTV_LASSO.SS
MTV_LASSO_corr
spring_corr
0
20
40
60
80
Genetic position (in cM)
Number of selections
1
2
3
4
5
6
7
8
9

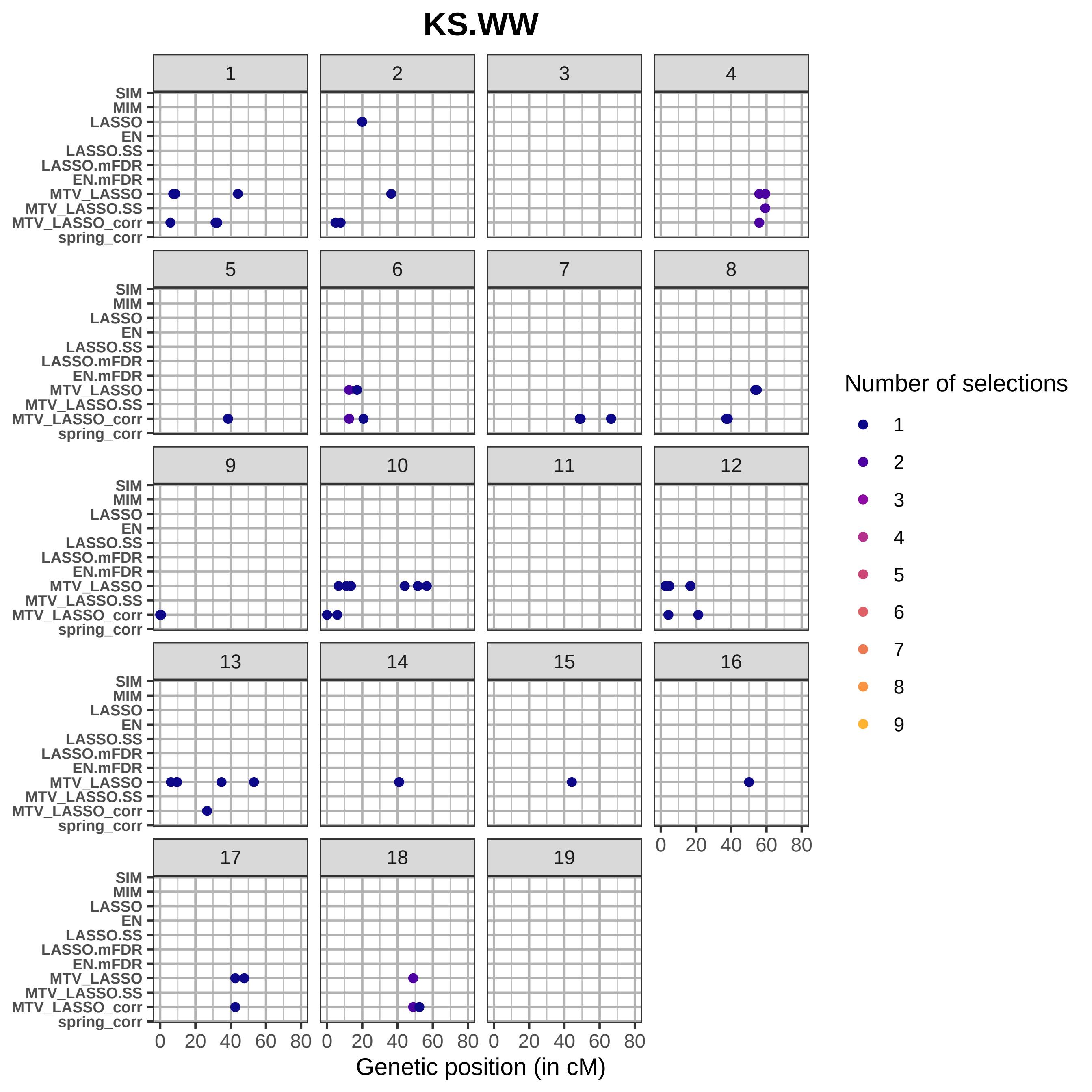

KS.WW
1
2
3
4
5
6
7
8
9
10
11
12
13
14
15
16
17
18
19
SIM
MIM
LASSO
EN
LASSO.SS
LASSO.mFDR
EN.mFDR
MTV_LASSO
MTV_LASSO.SS
MTV_LASSO_corr
spring_corr
0
20
40
60
80
Genetic position (in cM)
Number of selections
1
2
3
4
5
6
7
8
9

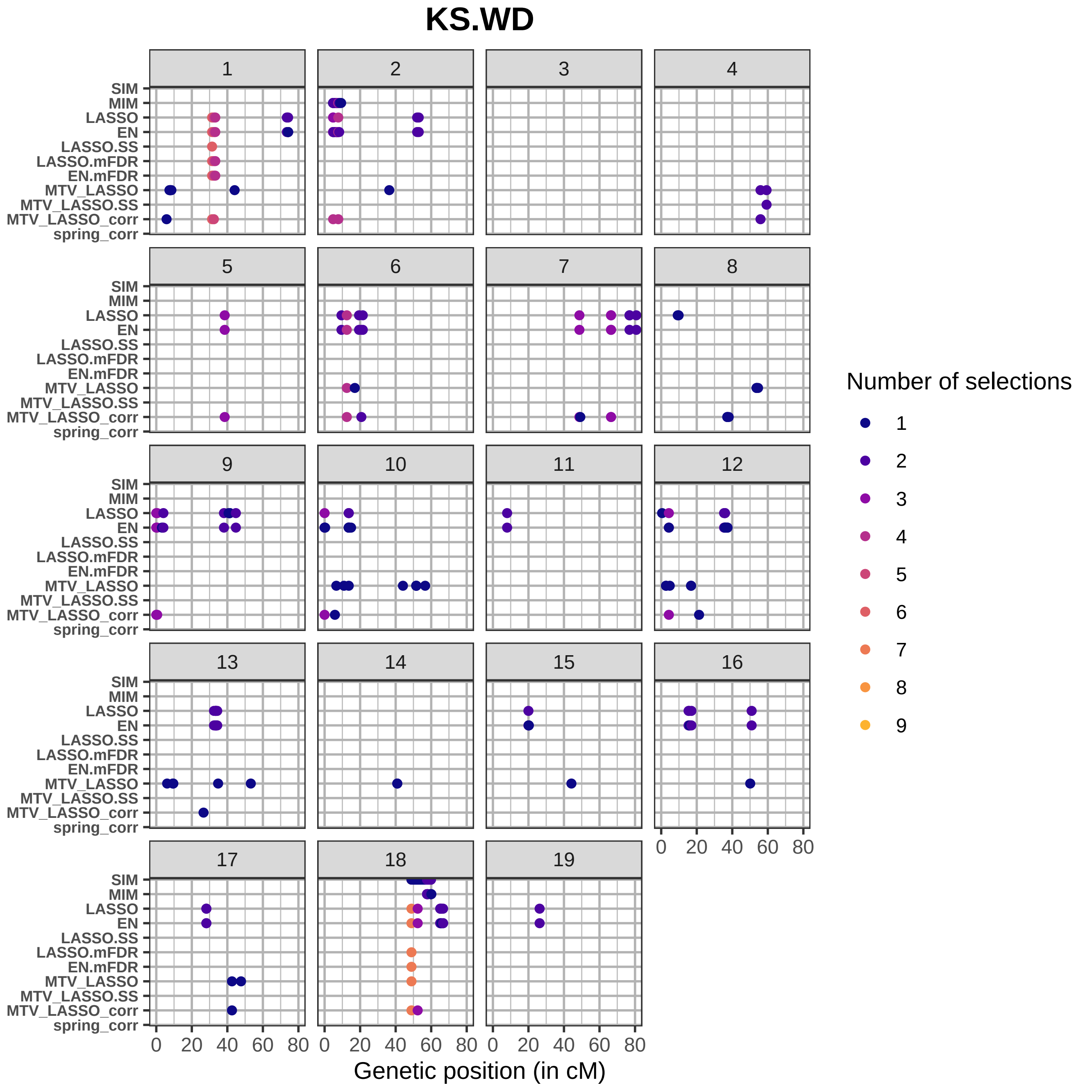

KS.WD
1
2
3
4
5
6
7
8
9
10
11
12
13
14
15
16
17
18
19
SIM
MIM
LASSO
EN
LASSO.SS
LASSO.mFDR
EN.mFDR
MTV_LASSO
MTV_LASSO.SS
MTV_LASSO_corr
spring_corr
0 20 40 60 80
Genetic position (in cM)
Number of selections
1
2
3
4
5
6
7
8
9

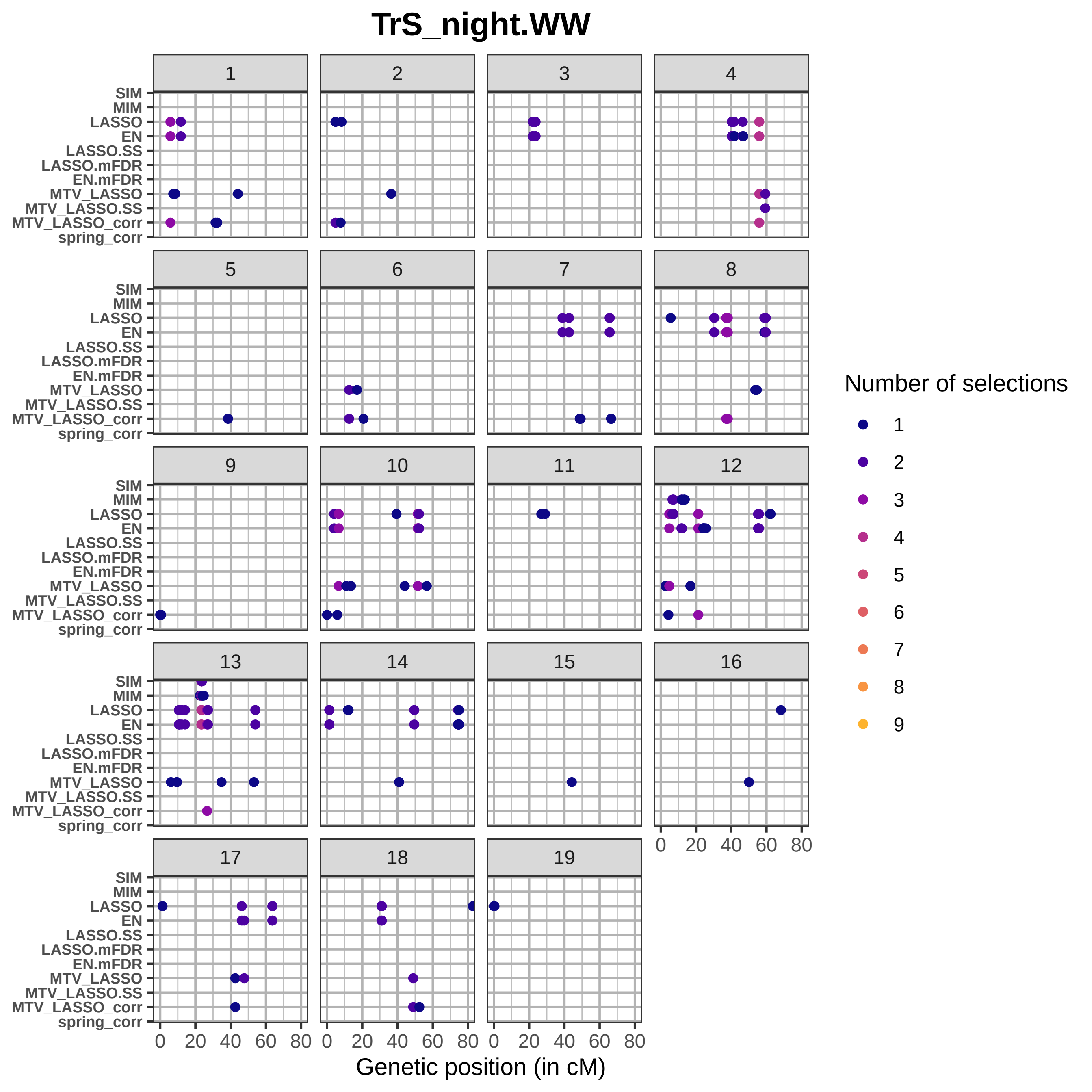
TrS_night.WW
1
2
3
4
5
6
7
8
9
10
11
12
13
14
15
16
17
18
19
SIM
MIM
LASSO
EN
LASSO.SS
LASSO.mFDR
EN.mFDR
MTV_LASSO
MTV_LASSO.SS
MTV_LASSO_corr
spring_corr
0 20 40 60 80
Genetic position (in cM)
Number of selections
1
2
3
4
5
6
7
8
9

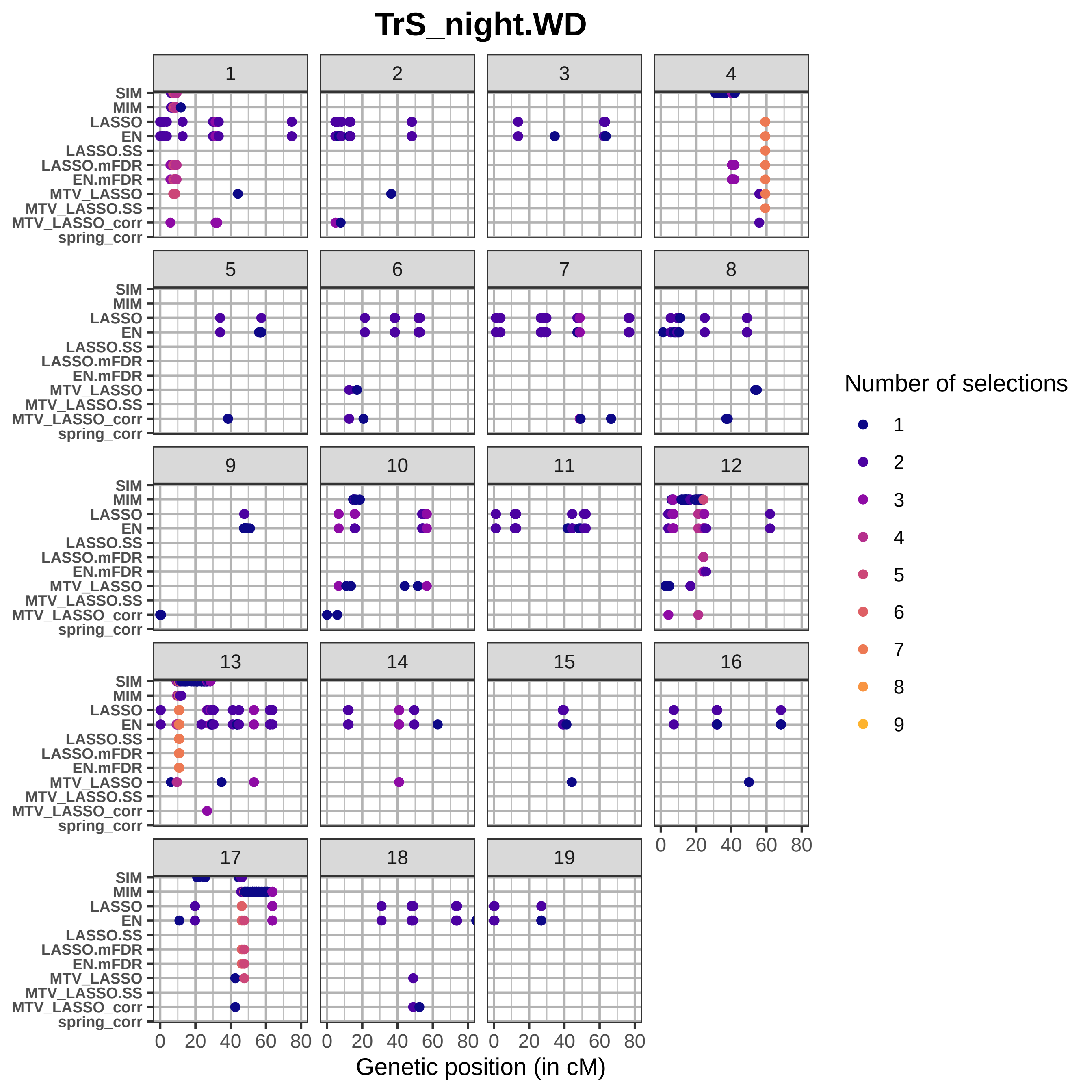
TrS_night.WD
1
2
3
4
5
6
7
8
9
10
11
12
13
14
15
16
17
18
19
SIM
MIM
LASSO
EN
LASSO.SS
LASSO.mFDR
EN.mFDR
MTV_LASSO
MTV_LASSO.SS
MTV_LASSO_corr
spring_corr
0
20
40
60
80
Genetic position (in cM)
Number of selections
1
2
3
4
5
6
7
8
9

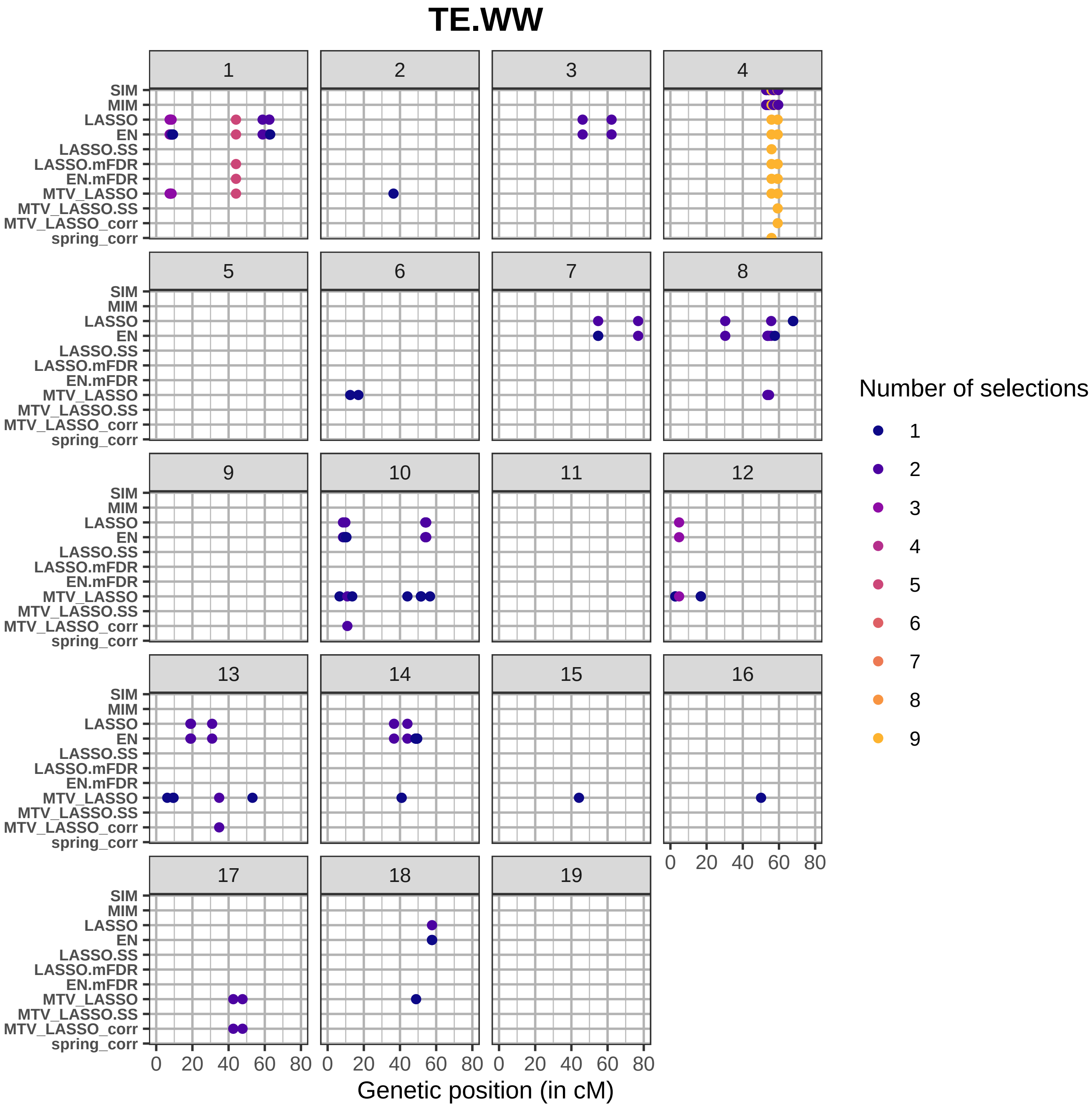
TE.WW
1
2
3
4
5
6
7
8
9
10
11
12
13
14
15
16
17
18
19
SIM
MIM
LASSO
EN
LASSO.SS
LASSO.mFDR
EN.mFDR
MTV_LASSO
MTV_LASSO.SS
MTV_LASSO_corr
spring_corr
0
20
40
60
80
Genetic position (in cM)
Number of selections
1
2
3
4
5
6
7
8
9

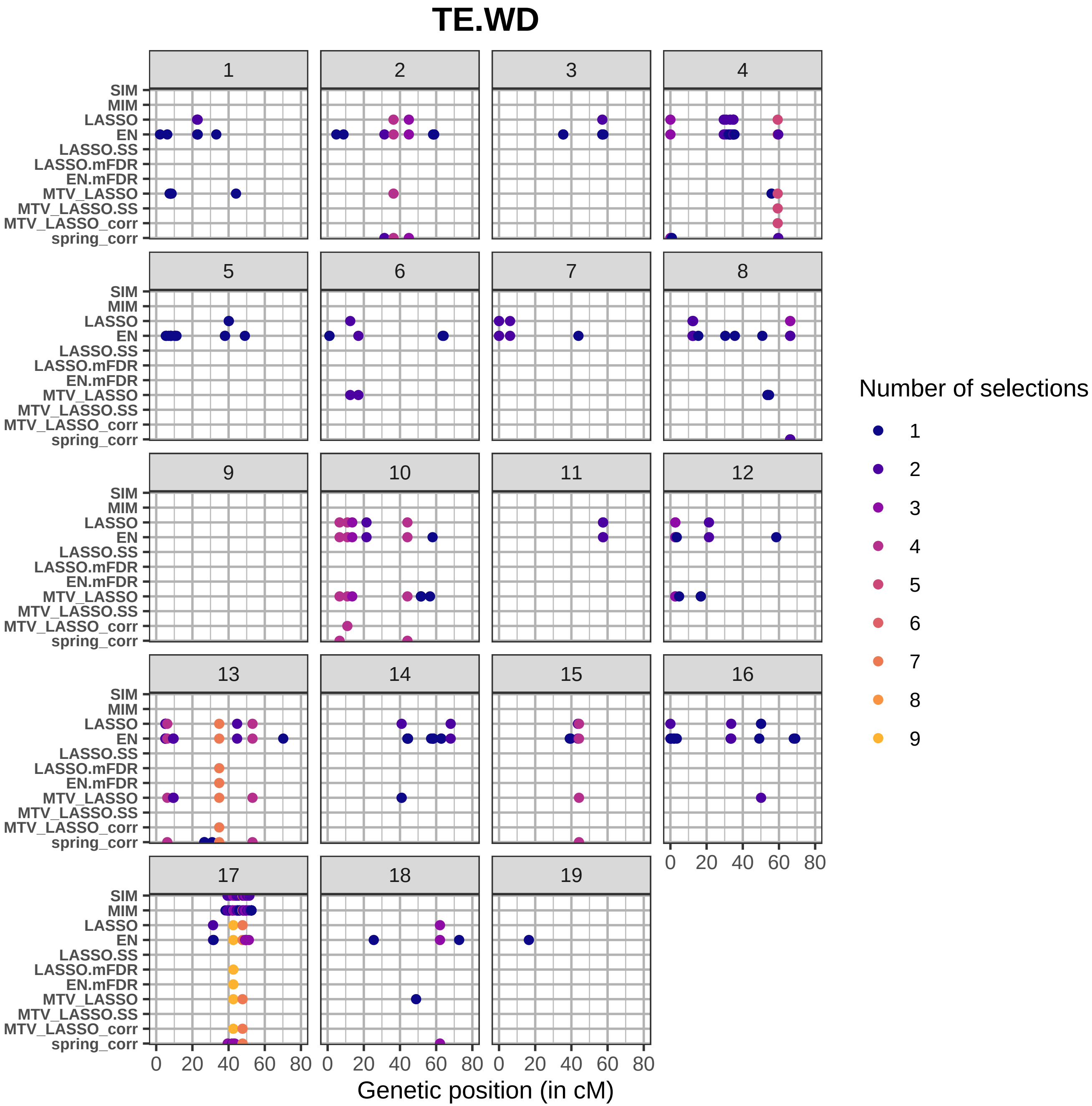
TE.WD
1
2
3
4
5
6
7
8
9
10
11
12
13
14
15
16
17
18
19
SIM
MIM
LASSO
EN
LASSO.SS
LASSO.mFDR
EN.mFDR
MTV_LASSO
MTV_LASSO.SS
MTV_LASSO_corr
spring_corr
0
20
40
60
80
Genetic position (in cM)
Number of selections
1
2
3
4
5
6
7
8
9

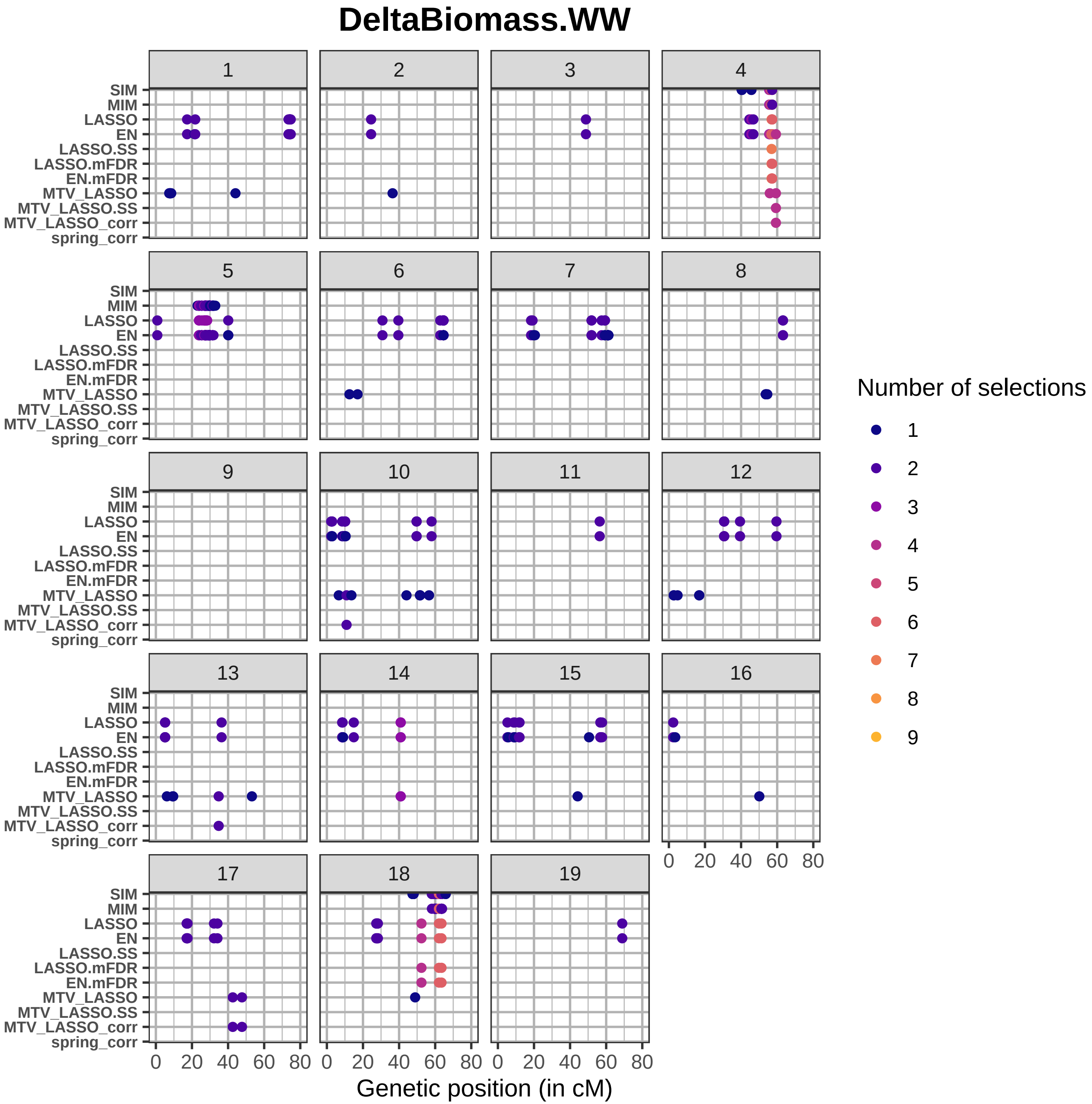

DeltaBiomass.WW
1
2
3
4
5
6
7
8
9
10
11
12
13
14
15
16
17
18
19
SIM
MIM
LASSO
EN
LASSO.SS
LASSO.mFDR
EN.mFDR
MTV_LASSO
MTV_LASSO.SS
MTV_LASSO_corr
spring_corr
0
20
40
60
80
Genetic position (in cM)
Number of selections
1
2
3
4
5
6
7
8
9

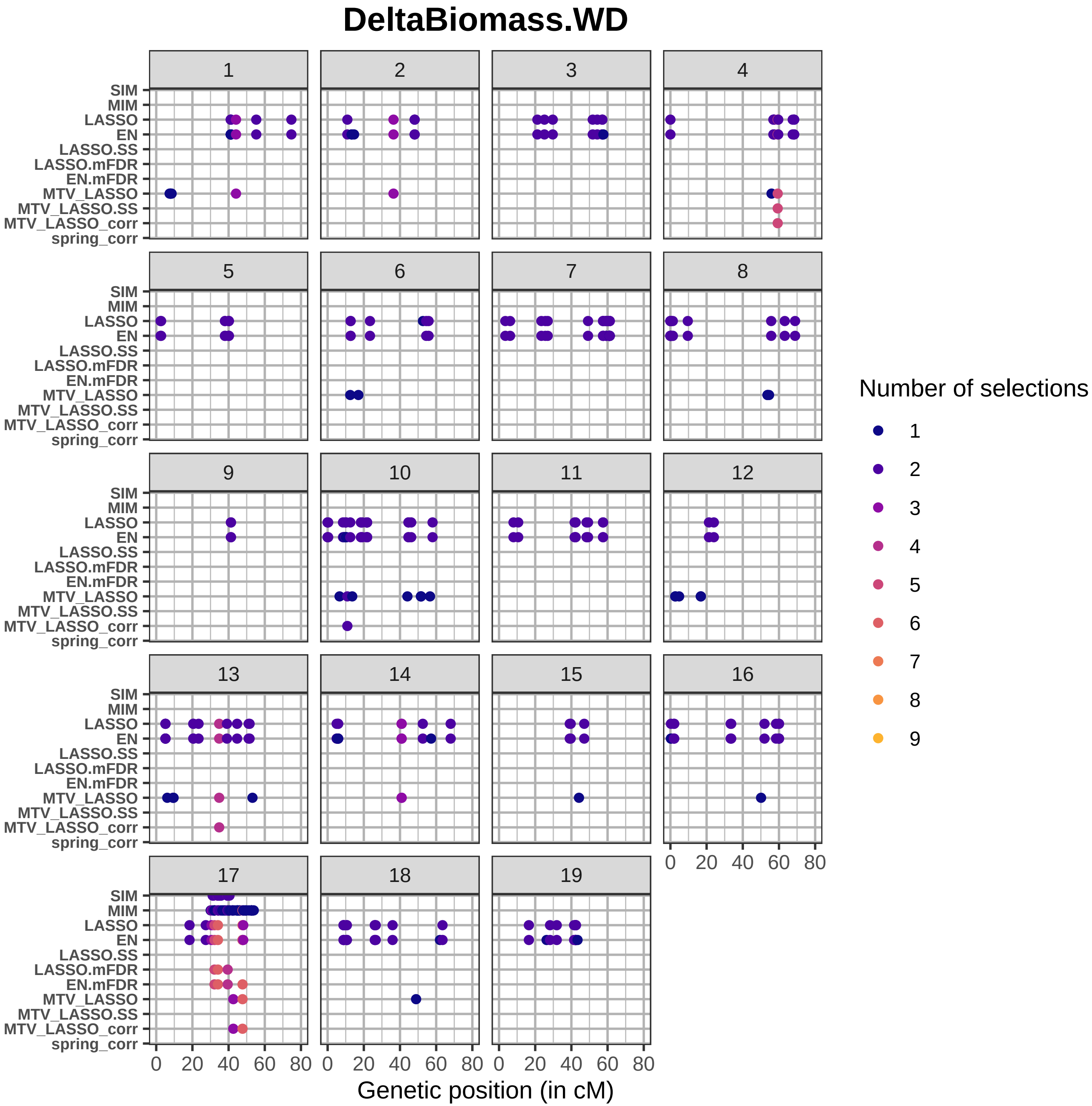
DeltaBiomass.WD
1
2
3
4
5
6
7
8
9
10
11
12
13
14
15
16
17
18
19
SIM
MIM
LASSO
EN
LASSO.SS
LASSO.mFDR
EN.mFDR
MTV_LASSO
MTV_LASSO.SS
MTV_LASSO_corr
spring_corr
0
20
40
60
80
Genetic position (in cM)
Number of selections
1
2
3
4
5
6
7
8
9
