# Supplementary material for: Harnessing multivariate, penalized regression methods for genomic prediction and QTL detection of drought-related traits in grapevine
Source: G3 (Bethesda). 2021 Jul 22;11(9):jkab248. doi: 10.1093/g3journal/jkab248 (PMC8496232; doi:10.1093/g3journal/jkab248)
Supplement: jkab248_Supplementary_Data [file jkab248_supplementary_data.zip › jkab248-suppl_data/GENETICS-G3-2021-402101-s02.zip!/supp/FigureS2_BLUP_comparison_old-new.pdf]

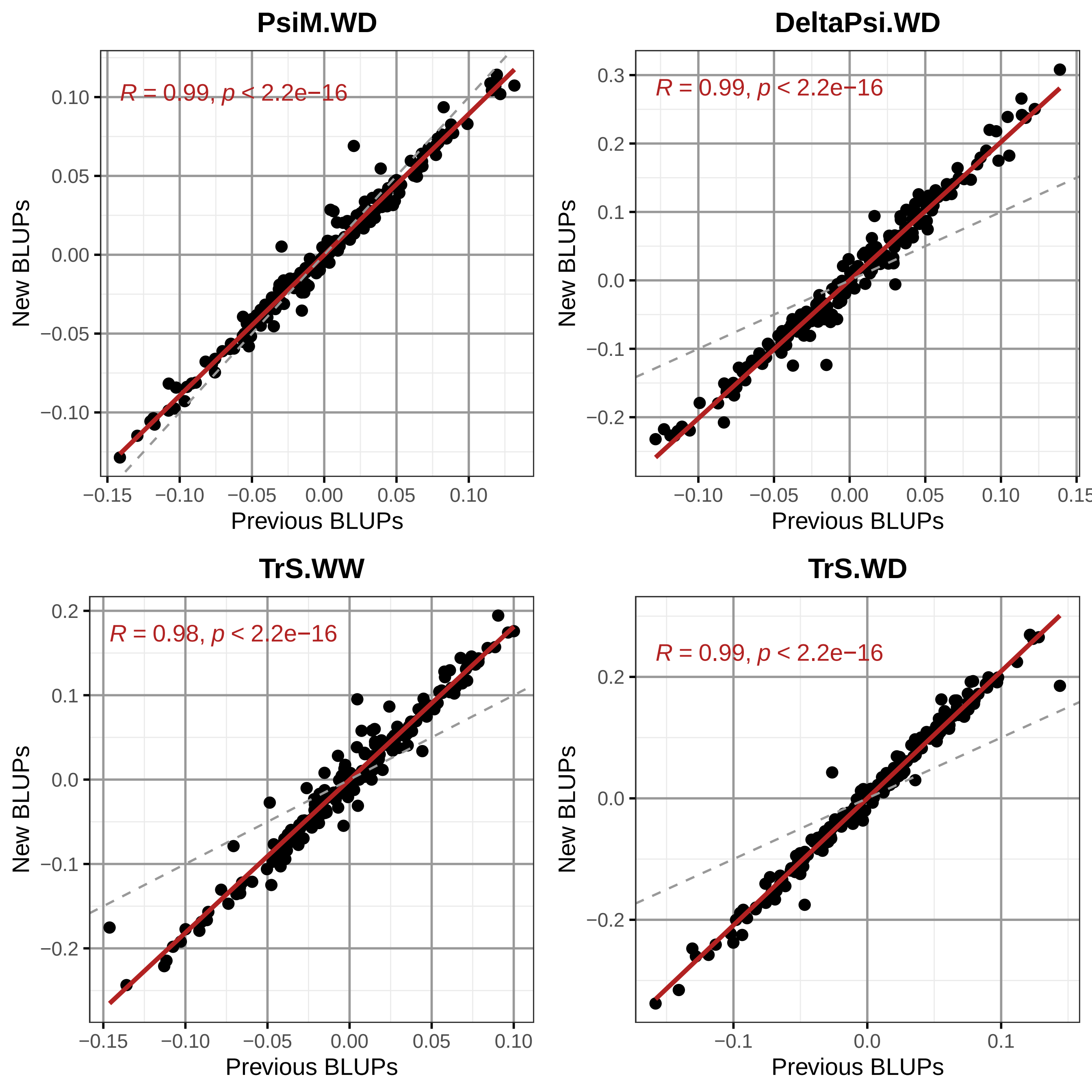
PsiM.WD
R = 0.99, p < 2.2e−16
New BLUPs
Previous BLUPs
0.10
0.05
0.00
−0.05
−0.10
−0.15
DeltaPsi.WD
R = 0.99, p < 2.2e−16
New BLUPs
Previous BLUPs
0.3
0.2
0.1
0.0
−0.1
−0.2
0.15
TrS.WW
R = 0.98, p < 2.2e−16
New BLUPs
Previous BLUPs
TrS.WD
R = 0.99, p < 2.2e−16
New BLUPs
Previous BLUPs

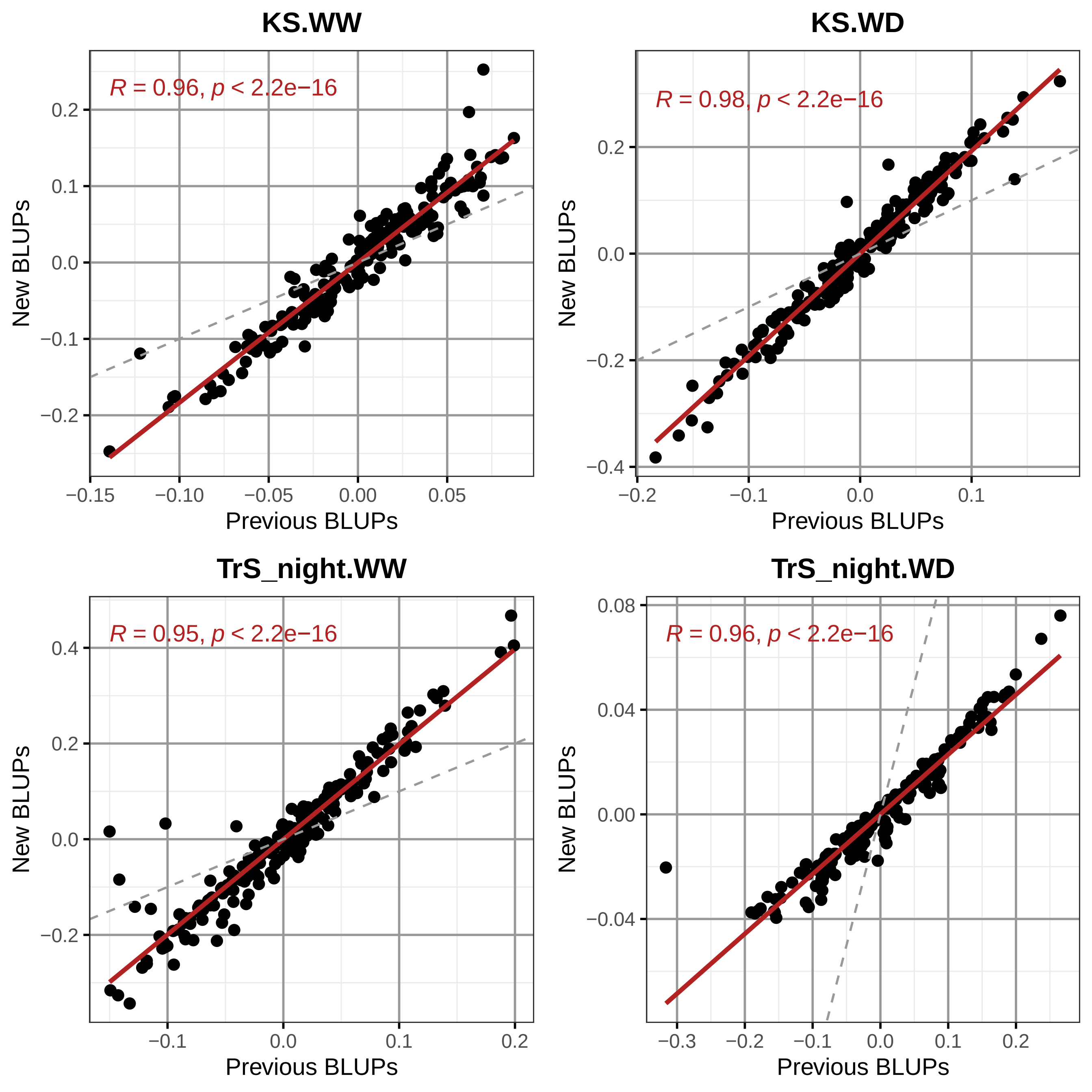
KS.WW
R = 0.96, p < 2.2e−16
New BLUPs
Previous BLUPs
KS.WD
R = 0.98, p < 2.2e−16
New BLUPs
Previous BLUPs
TrS_night.WW
R = 0.95, p < 2.2e−16
New BLUPs
Previous BLUPs
TrS_night.WD
R = 0.96, p < 2.2e−16
New BLUPs
Previous BLUPs

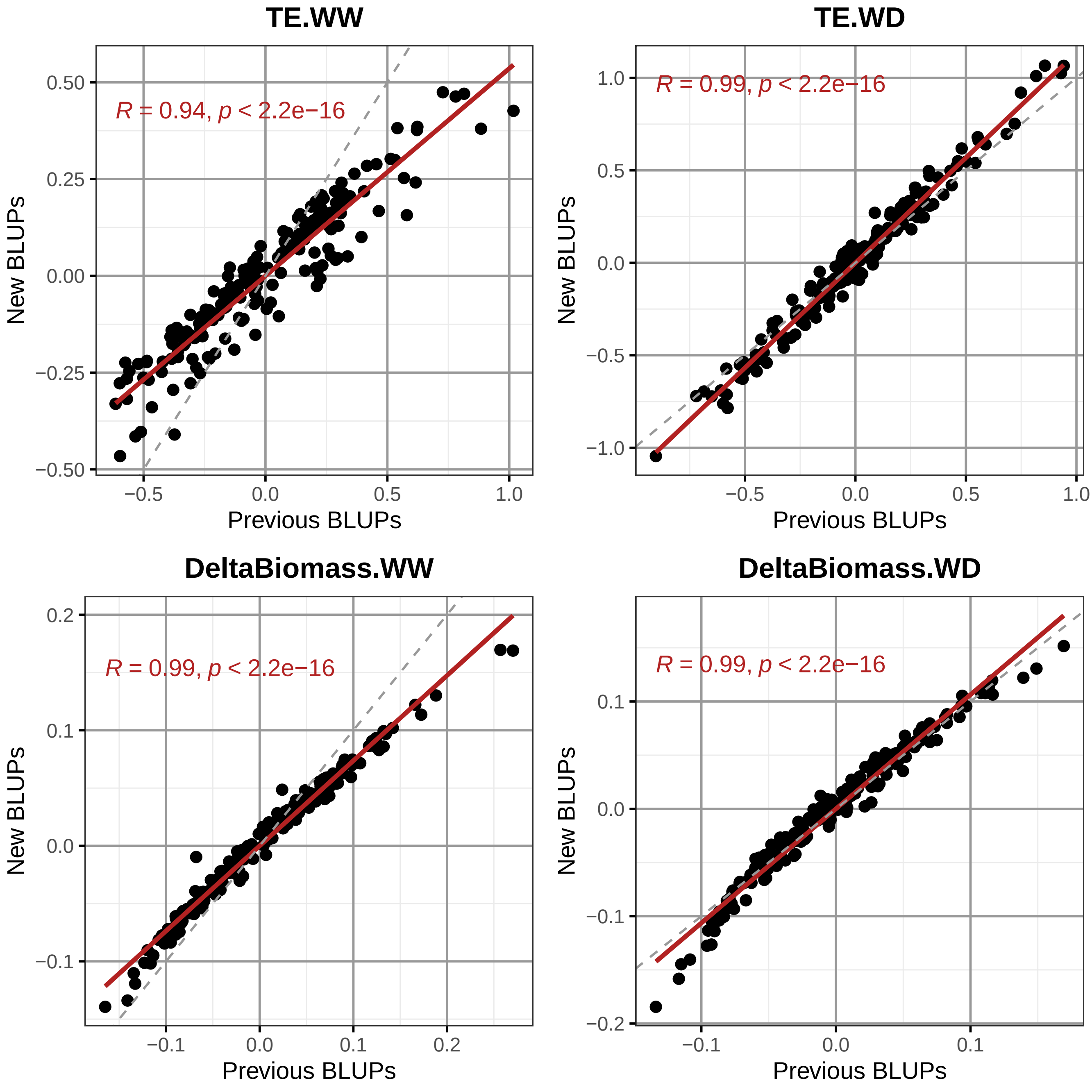

TE.WW
R = 0.94, p < 2.2e−16
New BLUPs
Previous BLUPs
TE.WD
R = 0.99, p < 2.2e−16
New BLUPs
Previous BLUPs
DeltaBiomass.WW
R = 0.99, p < 2.2e−16
New BLUPs
Previous BLUPs
DeltaBiomass.WD
R = 0.99, p < 2.2e−16
New BLUPs
Previous BLUPs
