# Supplementary material for: Harnessing multivariate, penalized regression methods for genomic prediction and QTL detection of drought-related traits in grapevine
Source: G3 (Bethesda). 2021 Jul 22;11(9):jkab248. doi: 10.1093/g3journal/jkab248 (PMC8496232; doi:10.1093/g3journal/jkab248)
Supplement: jkab248_Supplementary_Data [file jkab248_supplementary_data.zip › jkab248-suppl_data/GENETICS-G3-2021-402101-s02.zip!/supp/FigureS19_boxplots_most_reliable_QTLs.pdf]

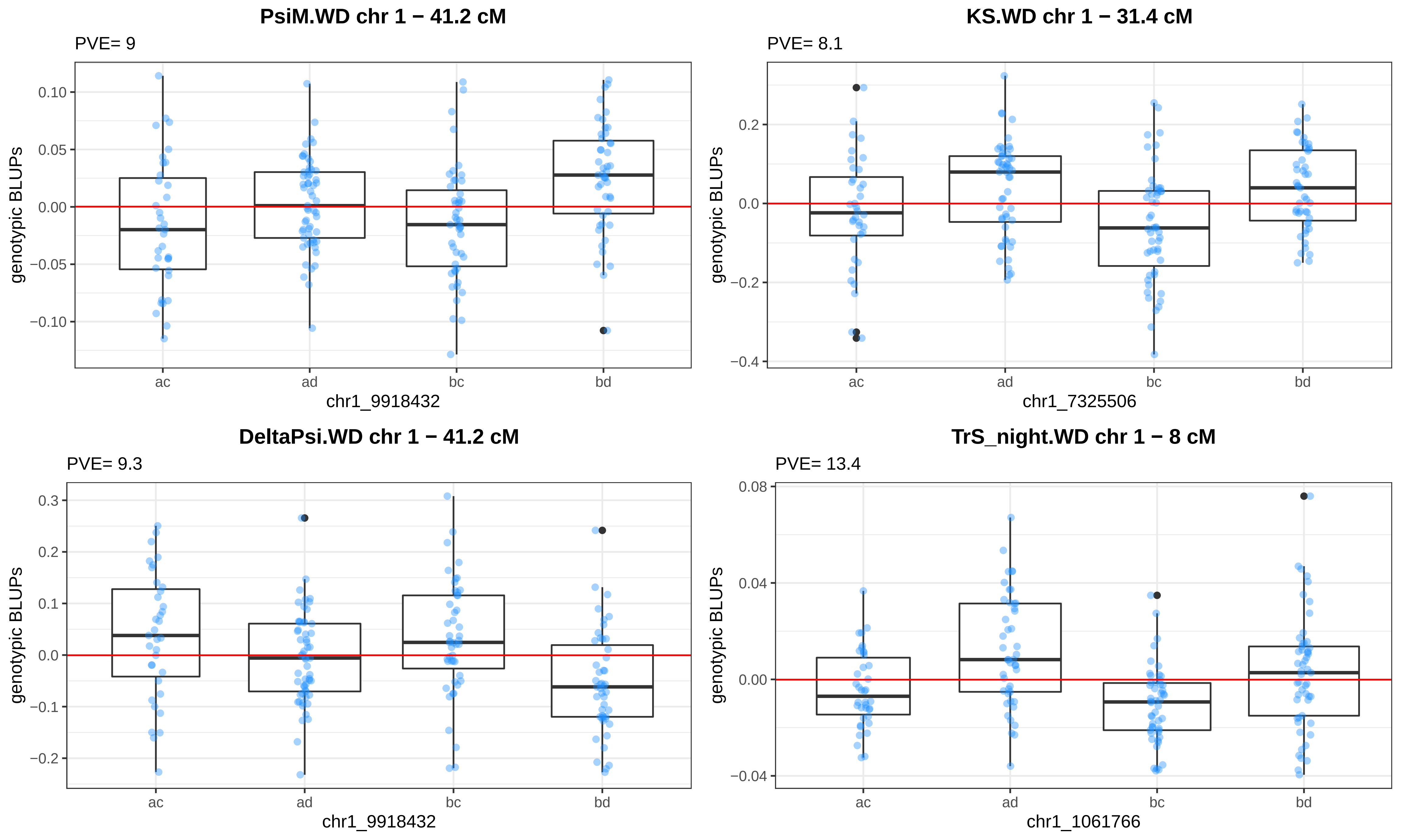
PsiM.WD chr 1 − 41.2 cM
PVE= 9
0.10
0.05
0.00
−0.05
−0.10
genotypic BLUPs
ac
ad
bc
bd
chr1_9918432
KS.WD chr 1 − 31.4 cM
PVE= 8.1
0.2
0.0
−0.2
−0.4
genotypic BLUPs
ac
ad
bc
bd
chr1_7325506
DeltaPsi.WD chr 1 − 41.2 cM
PVE= 9.3
0.3
0.2
0.1
0.0
−0.1
−0.2
genotypic BLUPs
ac
ad
bc
bd
chr1_9918432
TrS_night.WD chr 1 − 8 cM
PVE= 13.4
0.08
0.04
0.00
−0.04
genotypic BLUPs
ac
ad
bc
bd
chr1_1061766

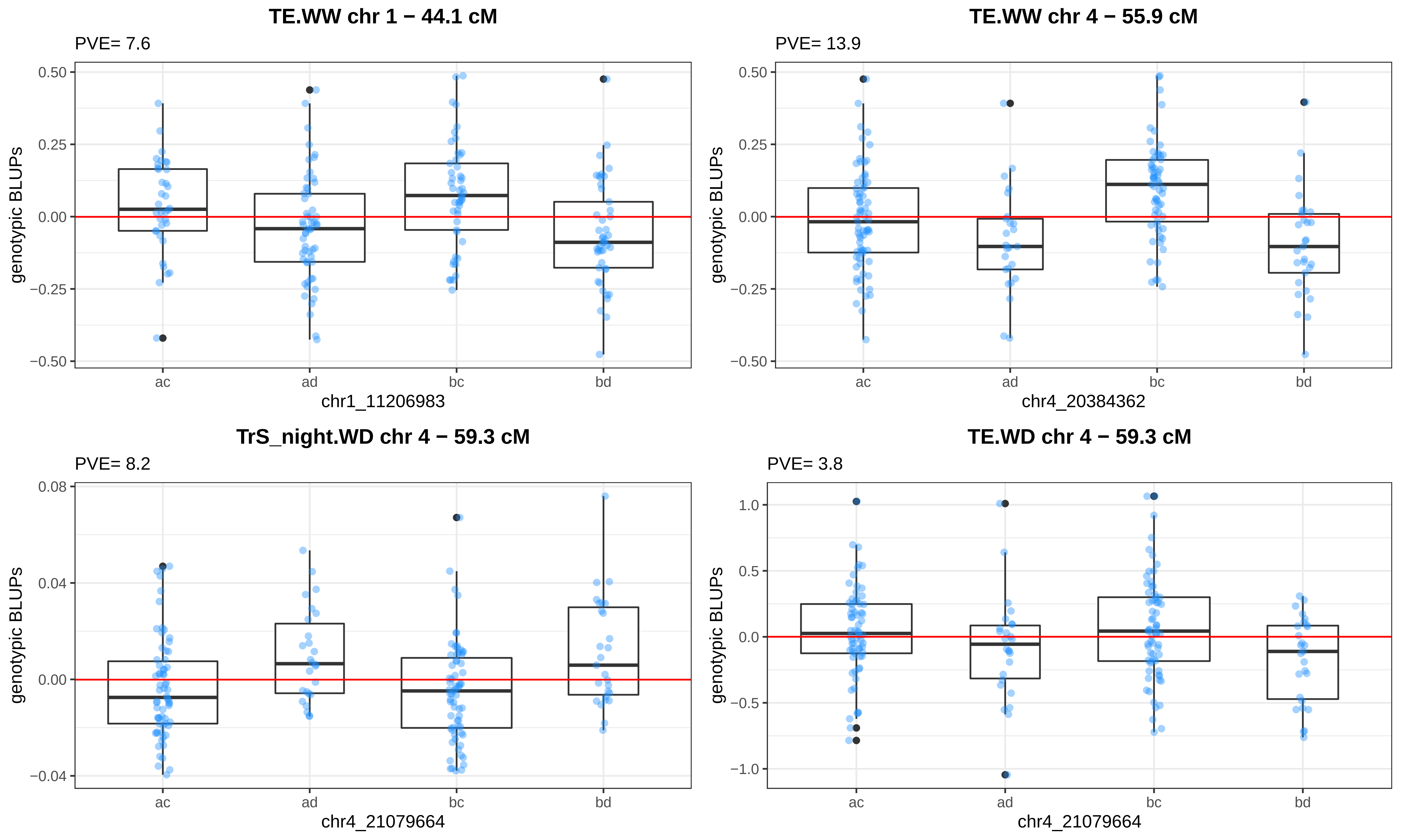
TE.WW chr 1 − 44.1 cM
PVE= 7.6
genotypic BLUPs
0.50
0.25
0.00
−0.25
−0.50
ac
ad
bc
bd
chr1_11206983
TE.WW chr 4 − 55.9 cM
PVE= 13.9
genotypic BLUPs
0.50
0.25
0.00
−0.25
−0.50
ac
ad
bc
bd
chr4_20384362
TrS_night.WD chr 4 − 59.3 cM
PVE= 8.2
genotypic BLUPs
0.08
0.04
0.00
−0.04
ac
ad
bc
bd
chr4_21079664
TE.WD chr 4 − 59.3 cM
PVE= 3.8
genotypic BLUPs
1.0
0.5
0.0
−0.5
−1.0
ac
ad
bc
bd
chr4_21079664

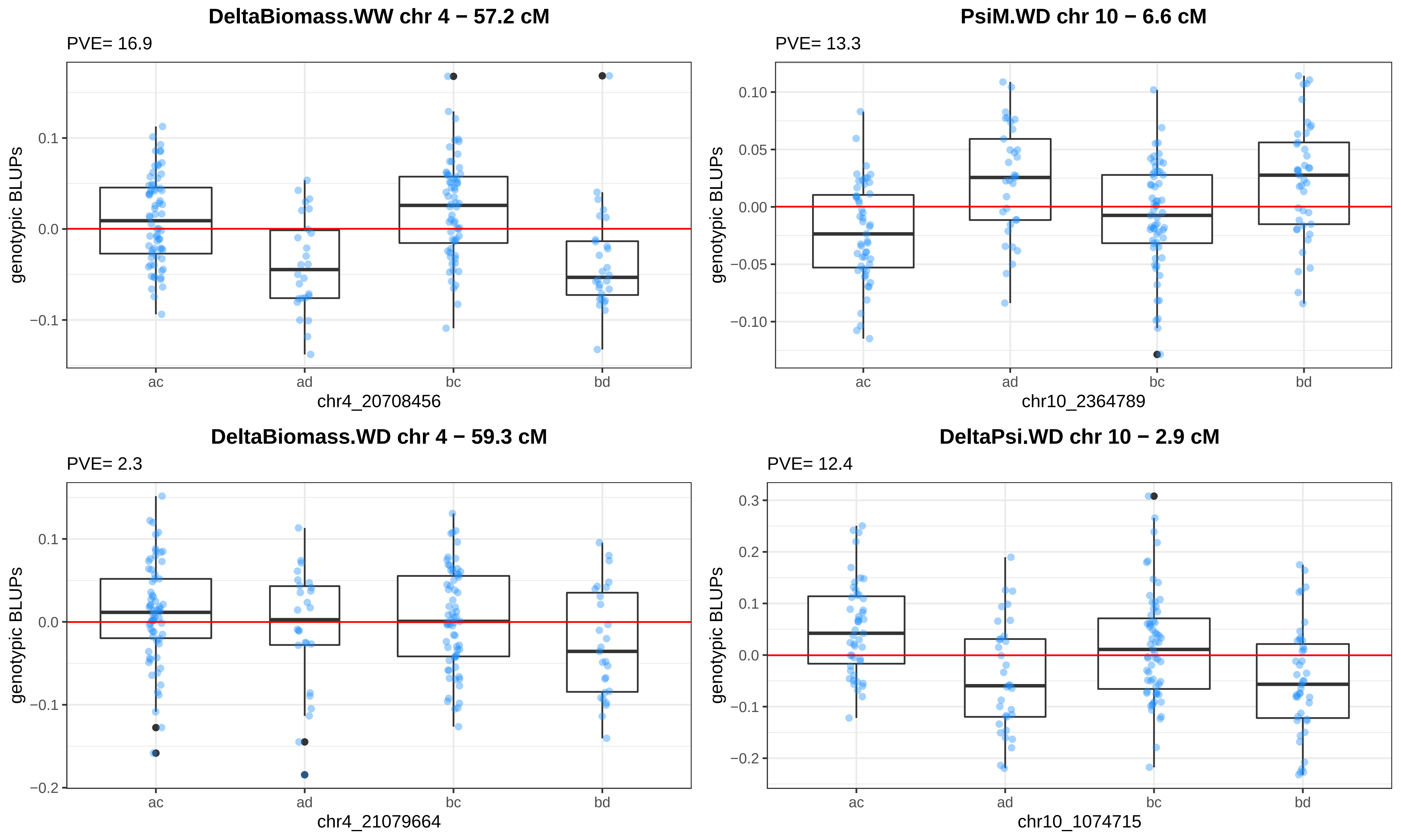
DeltaBiomass.WW chr 4 − 57.2 cM
PVE= 16.9
genotypic BLUPs
0.1
0.0
−0.1
ac
ad
bc
bd
chr4_20708456
PsiM.WD chr 10 − 6.6 cM
PVE= 13.3
genotypic BLUPs
0.10
0.05
0.00
−0.05
−0.10
ac
ad
bc
bd
chr10_2364789
DeltaBiomass.WD chr 4 − 59.3 cM
PVE= 2.3
genotypic BLUPs
0.1
0.0
−0.1
−0.2
ac
ad
bc
bd
chr4_21079664
DeltaPsi.WD chr 10 − 2.9 cM
PVE= 12.4
genotypic BLUPs
0.3
0.2
0.1
0.0
−0.1
−0.2
ac
ad
bc
bd
chr10_1074715

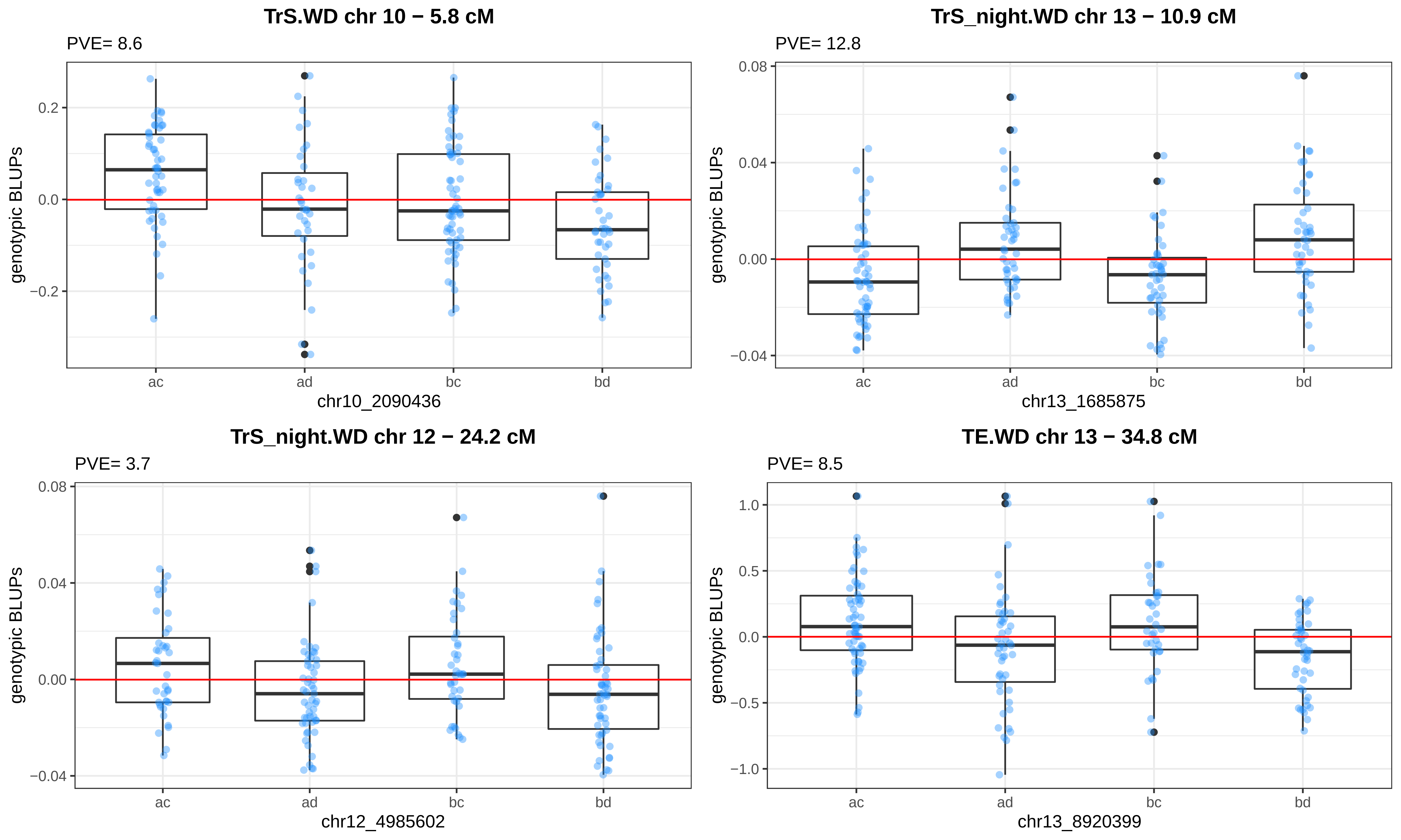

TrS.WD chr 10 − 5.8 cM
PVE= 8.6
genotypic BLUPs
0.2
0.0
−0.2
ac
ad
bc
bd
chr10_2090436
TrS_night.WD chr 13 − 10.9 cM
PVE= 12.8
genotypic BLUPs
0.08
0.04
0.00
−0.04
ac
ad
bc
bd
chr13_1685875
TrS_night.WD chr 12 − 24.2 cM
PVE= 3.7
genotypic BLUPs
0.08
0.04
0.00
−0.04
ac
ad
bc
bd
chr12_4985602
TE.WD chr 13 − 34.8 cM
PVE= 8.5
genotypic BLUPs
1.0
0.5
0.0
−0.5
−1.0
ac
ad
bc
bd
chr13_8920399

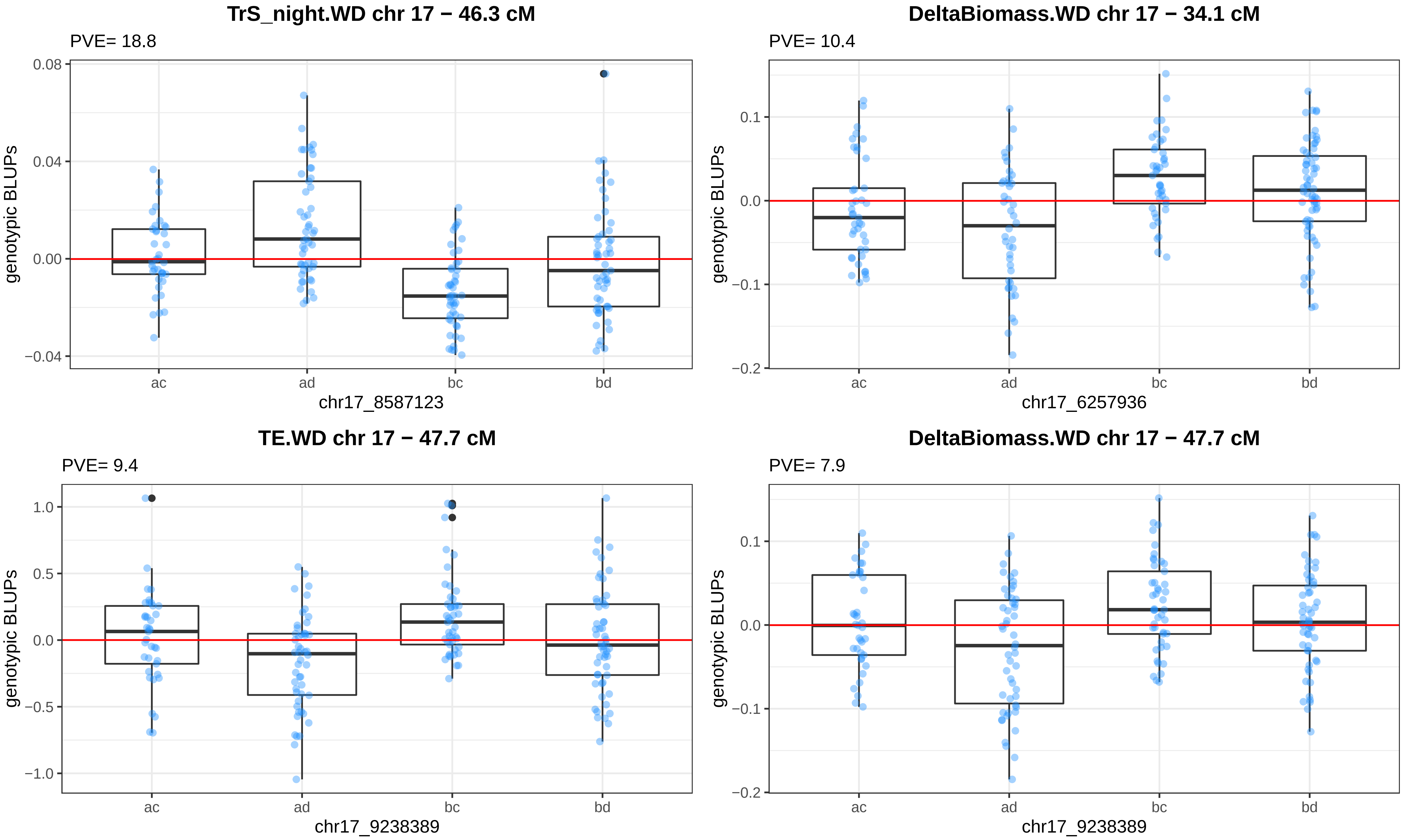
TrS_night.WD chr 17 − 46.3 cM
PVE= 18.8
genotypic BLUPs
0.08
0.04
0.00
−0.04
ac
ad
bc
bd
chr17_8587123
DeltaBiomass.WD chr 17 − 34.1 cM
PVE= 10.4
genotypic BLUPs
0.1
0.0
−0.1
−0.2
ac
ad
bc
bd
chr17_6257936
TE.WD chr 17 − 47.7 cM
PVE= 9.4
genotypic BLUPs
1.0
0.5
0.0
−0.5
−1.0
ac
ad
bc
bd
chr17_9238389
DeltaBiomass.WD chr 17 − 47.7 cM
PVE= 7.9
genotypic BLUPs
0.1
0.0
−0.1
−0.2
ac
ad
bc
bd
chr17_9238389

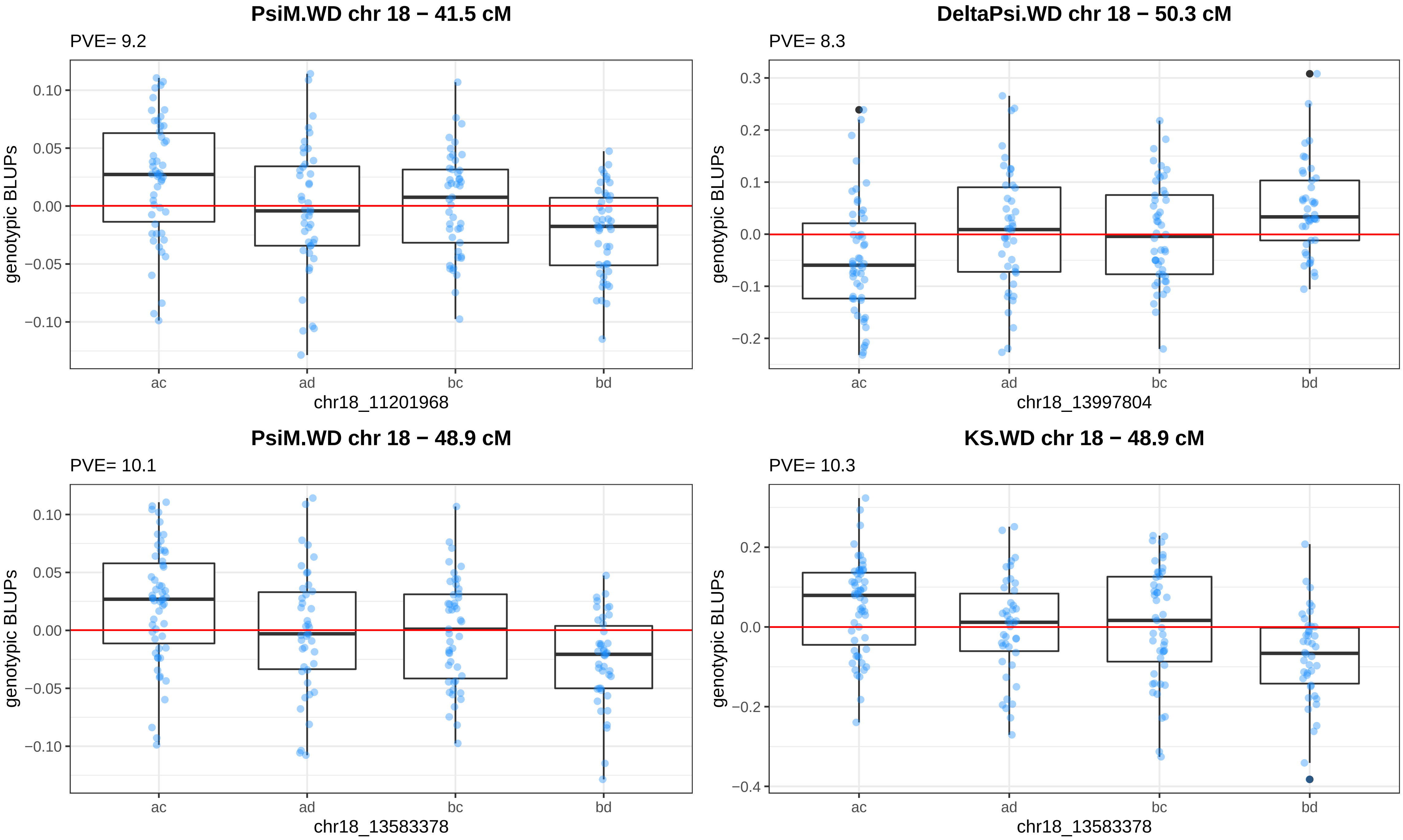

PsiM.WD chr 18 − 41.5 cM
PVE= 9.2
genotypic BLUPs
ac
ad
bc
bd
chr18_11201968
DeltaPsi.WD chr 18 − 50.3 cM
PVE= 8.3
genotypic BLUPs
ac
ad
bc
bd
chr18_13997804
PsiM.WD chr 18 − 48.9 cM
PVE= 10.1
genotypic BLUPs
ac
ad
bc
bd
chr18_13583378
KS.WD chr 18 − 48.9 cM
PVE= 10.3
genotypic BLUPs
ac
ad
bc
bd
chr18_13583378

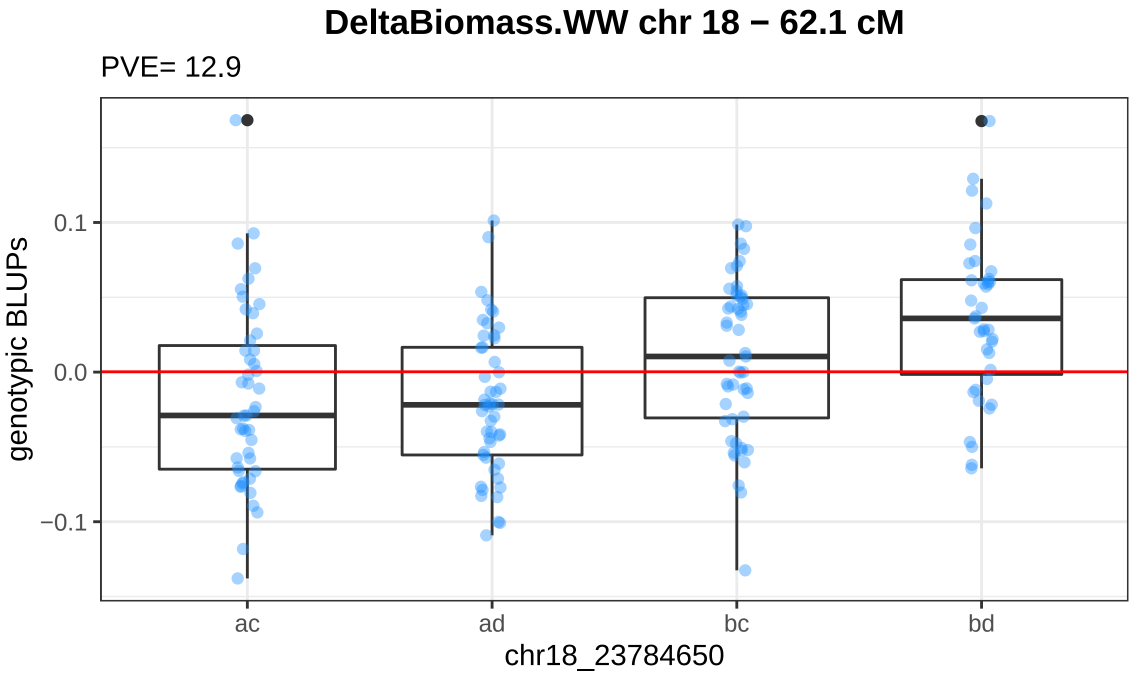
DeltaBiomass.WW chr 18 − 62.1 cM
PVE= 12.9
genotypic BLUPs
0.1
0.0
−0.1
ac
ad
bc
bd
chr18_23784650
