# Supplementary material for: Harnessing multivariate, penalized regression methods for genomic prediction and QTL detection of drought-related traits in grapevine
Source: G3 (Bethesda). 2021 Jul 22;11(9):jkab248. doi: 10.1093/g3journal/jkab248 (PMC8496232; doi:10.1093/g3journal/jkab248)
Supplement: jkab248_Supplementary_Data [file jkab248_supplementary_data.zip › jkab248-suppl_data/GENETICS-G3-2021-402101-s02.zip!/supp/supplementary_revised.pdf]

Supplemental information

Harnessing multivariate, penalized regression methods for genomic prediction and QTL detection of drought-related traits in grapevine

Authors: Brault C, Doligez A, Le Cunff L, Coupel-Ledru A, Simonneau T, Chiquet J, This P, Flutre T

Table of content:

Table S1 Conversion from genotypic class (in ab x cd segregation) to biallelic gene-dose.

Figure S2 Comparison between published and re-computed genotypic BLUPs.

Figure S3 Comparison between published and re-computed broad-sense heritability estimates.

Figure S4 Correlation and distribution of BLUPs for experimental data.

Figure S5 Nested cross-validation scheme.

Figure S6 Hierarchical clustering of PhenoArch traits.

Figure S7 Linkage Disequilibrium pattern in Syrah x Grenache progeny.

Figure S8 Prediction accuracy for all methods on simulated data.

Table S9 Genomic prediction performance on simulated data for all methods and configurations.

Figure S10 Prediction accuracy with 100 and 1000 simulated QTLs.

Figure S11 Prediction accuracy at different heritability values among traits.

Figure S12 Prediction accuracy with simulated data from Jia & Jannink (2012).

Figure S13 Partial Area Under the Curve for FPR up to 0.1.

Table S14 Genomic prediction performance on experimental grapevine data for all traits and methods.

Table S15 Results of marker selection for all traits and methods.

Figure S16 Genetic position of selected markers per trait and for all methods.

Figure S17 Most reliable QTL intervals displayed along the genetic map.

Table S18 Complementary information about most reliable QTLs.

Figure S19 Distribution of genotypic BLUPs according to genotypic class for each highly reliable QTL.

Table S20 List of genes underlying the most highly reliable QTLs with their annotations.

Table S21 List and functional classification of annotated genes underlying the highly reliable QTL detected on chromosome 4 for TrS_night.WD_LG4, TE.WW_LG4, TE.WD_LG4, DeltaBiomass.WW_LG4, DeltaBiomass.WD_LG4.

Figure S22 Prediction accuracy according to False Positive Rate for EN and EN.mFDR.

segreg	coding_ ab	phase	coding_phased	AC	AD	BC	BD	gene. dose. AC	gene. dose. AD	gene. dose. BC	gene. dose. BD
lmxll	abxaa	{0-}	abxaa	aa	aa	ba	ba	2	2	1	1
lmxll	abxaa	{1-}	baxaa	ba	ba	aa	aa	1	1	2	2
nnxnp	aaxab	{-0}	aaxab	aa	ab	aa	ab	2	1	2	1
nnxnp	aaxab	{-1}	aaxba	ab	aa	ab	aa	1	2	1	2
hkxhk	abxab	{00}	abxab	aa	ab	ba	bb	2	1	1	0
hkxhk	abxab	{01}	abxba	ab	aa	bb	ba	1	2	0	1
hkxhk	abxab	{10}	baxab	ba	bb	aa	ab	1	0	2	1
hkxhk	abxab	{11}	baxba	bb	ba	ab	aa	0	1	1	2

Table S1: Conversion from genotypic class (in ab x cd segregation) to biallelic gene-dose.
Table used to convert imputed genotypic class from Lep-MAP3 to additive gene dose according to the initial segregation of markers.

Grenache is considered as the father and Syrah the mother for the whole progeny for map construction

FigureS2_BLUPs_comparison_old-new.pdf
Figure S2: Comparison between published and re-computed genotypic BLUPs
For each trait, we plotted the new BLUPs with the BLUPs from Coupel-Ledru *et al.* (2014, 2016). The grey dashed line is the bisector and the red line is the regression line. Correlation between new and previous BLUPs as well as *p-value* are indicated.

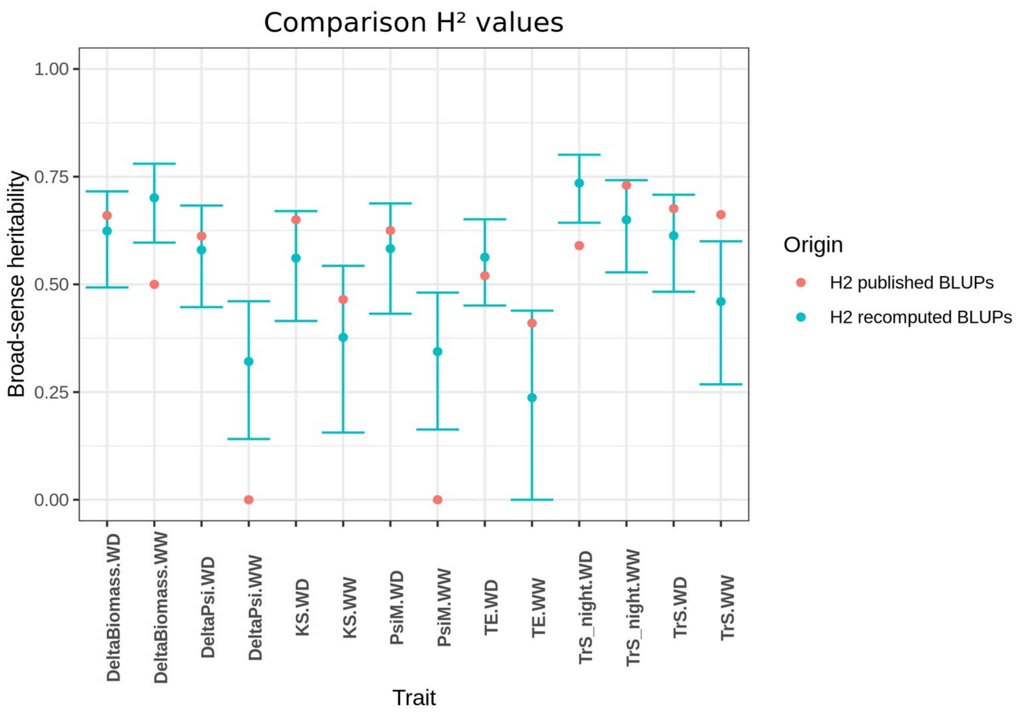

Figure S3: Comparison between published and re-computed broad-sense heritability estimates

Broad-sense heritability values estimated by Coupel-Ledru *et al.* (2014, 2016) are reported in red. Recomputed heritability values are reported in blue with the 95% confidence interval.

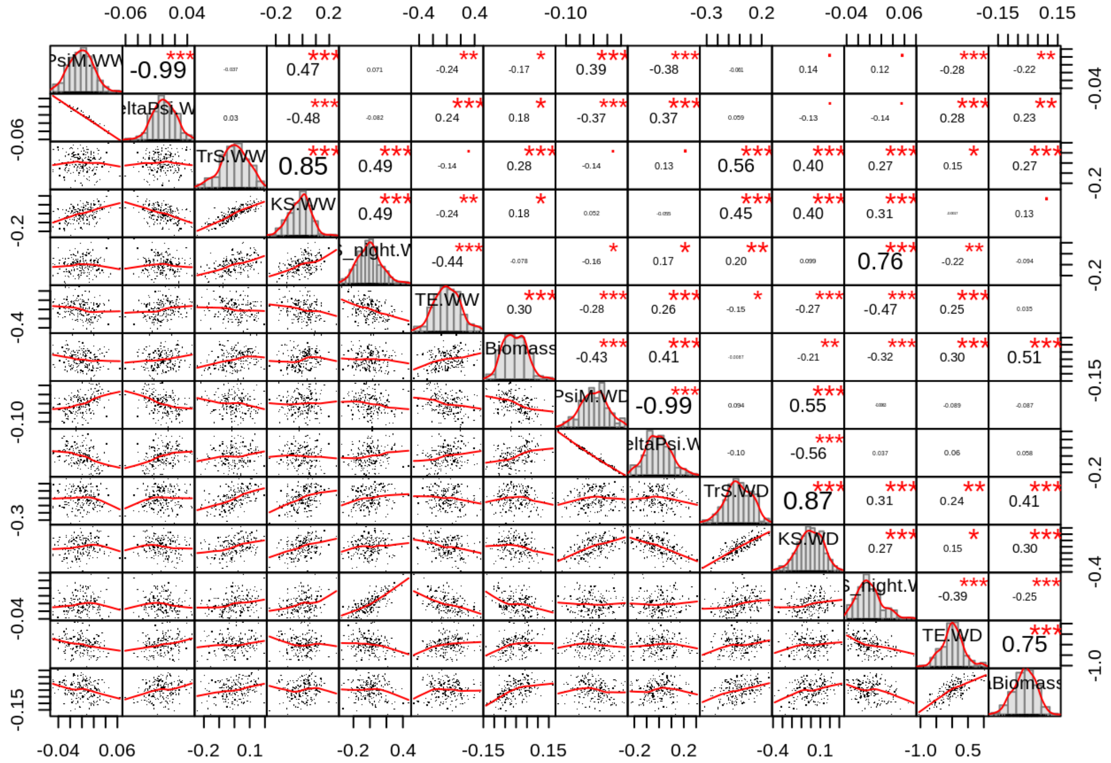

Figure S4: Correlation and distribution of BLUPs for experimental data

Lower panel: pairwise comparison of genotypic BLUP distributions with bivariate scatterplots with fitted lines (with local polynomes).

Upper panel: Pearson's correlation values and significant *p-values* ("." < 0.1, "*" < 0.05, "**" < 0.01, "***" < 0.001).

Diagonal panel: distributions of genotypic BLUPs for each trait

Trait order: *PsiM.WW*, *DeltaPsi.WW*, *TrS.WW, KS.WW, TrS_night.WW, TE.WW, DeltaBiomass.WW, PsiM.WD, DeltaPsi.WD, TrS.WD, KS.WD, TrS_night.WD, TE.WD, DeltaBiomass.WD.*

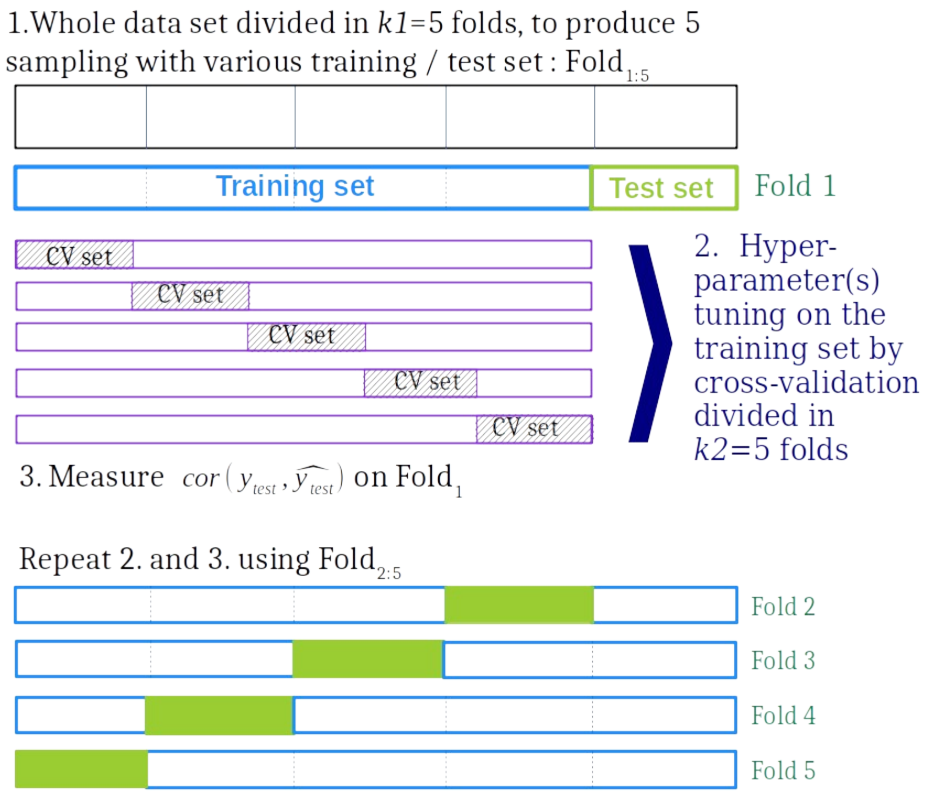

Figure S5: Nested cross-validation scheme

Procedure for cross-validation we applied on simulated and real data for prediction.

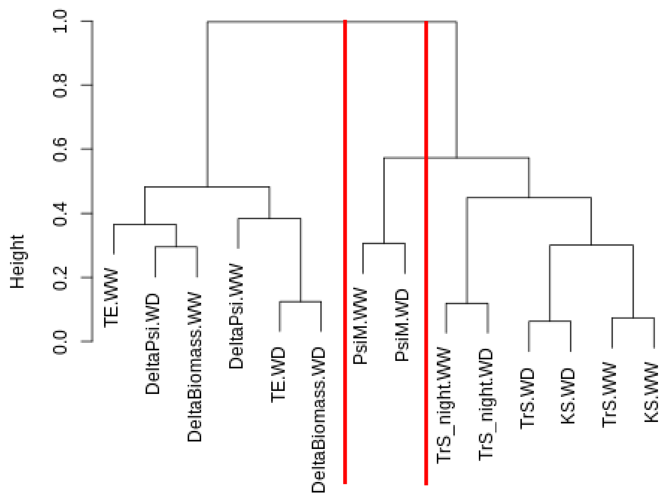

Figure S6: Hierarchical clustering of PhenoArch traits

Clusterisation was done with R/hclust function from stats package. Red bars were added and represent the three cluster of traits we have done.

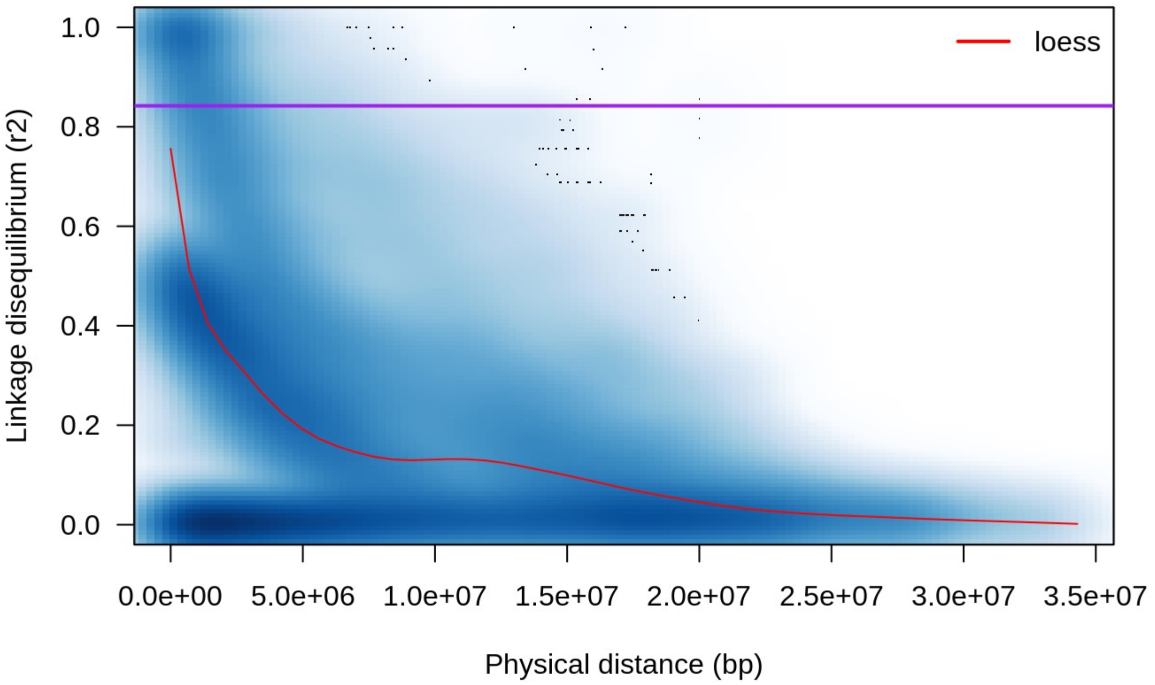

Figure S7: Linkage Disequilibrium pattern in Syrah x Grenache progeny

Linkage disequilibrium was calculated for each chromosome separately with the 3,961 SNP markers with estimLd function and plotted with plotLd function (both from rutilstimflutre R package). The purple line corresponds to the 95% quantile of the LD distribution (where r^2=0.84).

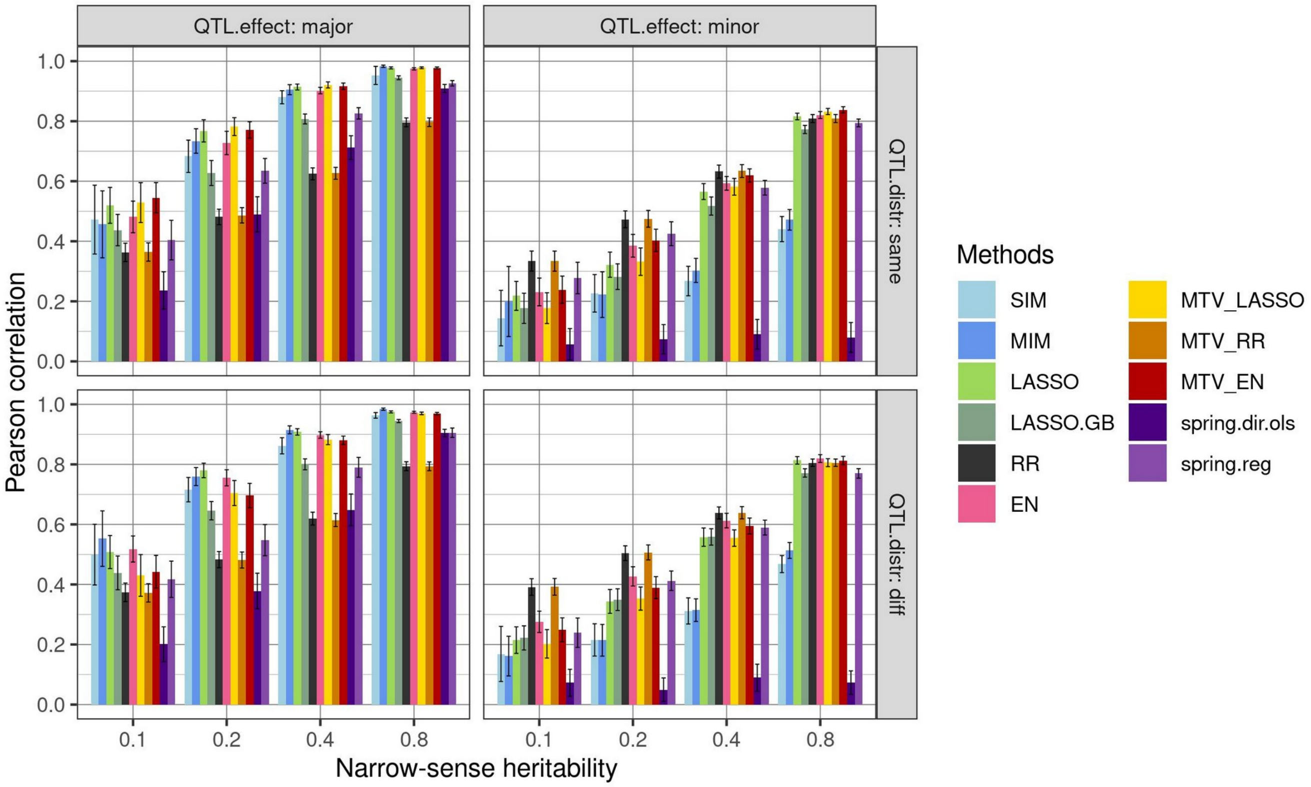

Figure S8: Prediction accuracy for all methods on simulated data

Genomic prediction accuracy (Pearson’s correlation between predicted and simulated genotypic values) of 11

methods applied to 3,961 markers and two simulated traits in a bi-parental population, with four heritability values and four QTL configurations (effect x distribution among traits). **Major**: 2 QTLs; **minor**: 50 QTLs; **same**: QTLs at the same positions for both traits; **diff**: QTLs at different positions between traits. For each heritability value and configuration, prediction accuracy was averaged over 100 values (2 traits x 10 simulation replicates x 5 cross-validation folds). The error bar corresponds to the 95% confidence interval around the mean. In comparison with Figure 1, four methods are added: SIM, LASSO.GB, MTV_RR and *spring.dir.ols*.

TableS9_results_all-methods.tsv

Table S9: Genomic prediction performance on simulated data for all methods and configurations

Genomic prediction performance, as measured by different metrics, of 11 methods applied to 3,961 markers and two simulated traits in a bi-parental population, with four heritability values and four QTL configurations (effect x distribution among traits). **Major**: 2 QTLs; **minor**: 50 QTLs; **same**: QTLs at the same positions for both traits; **diff**: QTLs at different positions between traits. Mean results are averaged over 2 traits, 10 simulation replicates x 5 cross-validation folds. For each metric measured, the standard error is also given (last columns, suffix "SE").

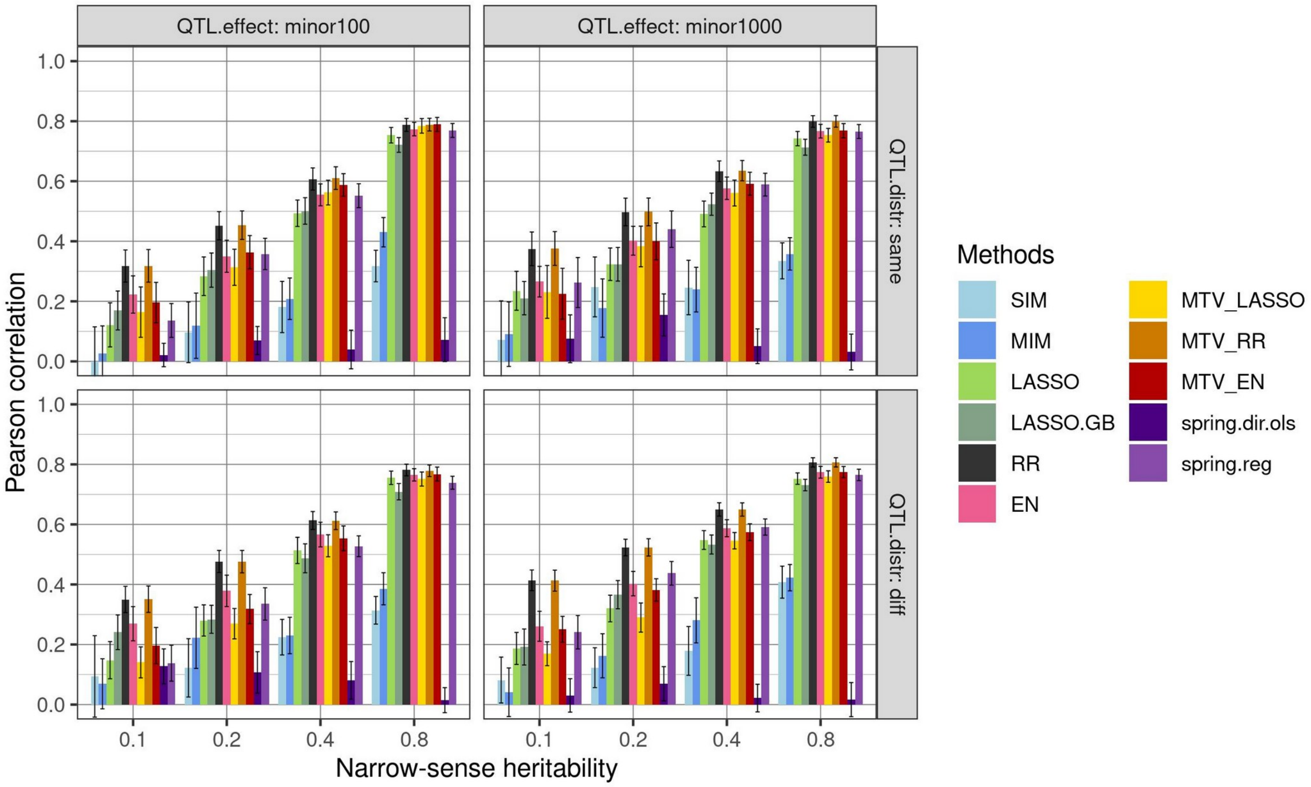

Figure S10: Prediction accuracy with 100 and 1000 simulated QTLs

Genomic prediction accuracy (Pearson's correlation between predicted and true genotypic values) of 11 methods applied to 3,961 markers and phenotypic data simulated in a bi-parental population with four heritability values and QTL configurations (effect x distribution among traits). **minor100**: 100 QTLs; **minor1000**: 1000 QTLs; **same**: QTLs at the same positions for both traits; **diff**: QTLs at different positions between traits. Results are averaged over 2 traits x 5 simulation replicates x 5 cross-validation folds. The error bar corresponds to the 95% confidence interval around the mean.

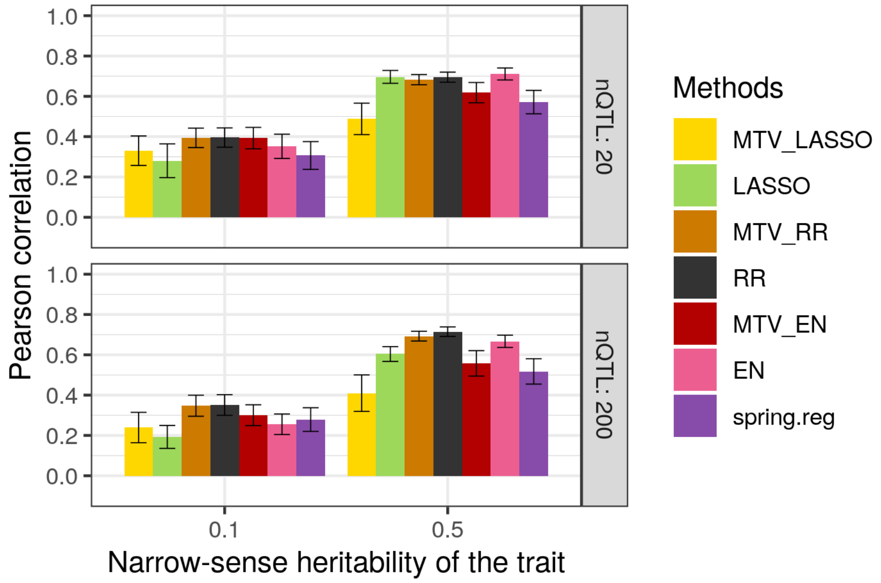

Figure S11: Prediction accuracy at different heritability values among traits

Genomic prediction accuracy (Pearson's correlation between predicted and simulated genotypic values) of seven methods applied to 3,961 markers and two simulated traits in a bi-parental population, with different heritability values (0.1 and 0.5), 20 or 200 QTLs, a genetic correlation of 0.5 and a genetic variance of 1. QTLs were the same for both traits. For each trait, results are averaged over 10 simulation replicates x 5 cross-validation folds. The error bar corresponds to the 95% confidence interval around the mean.

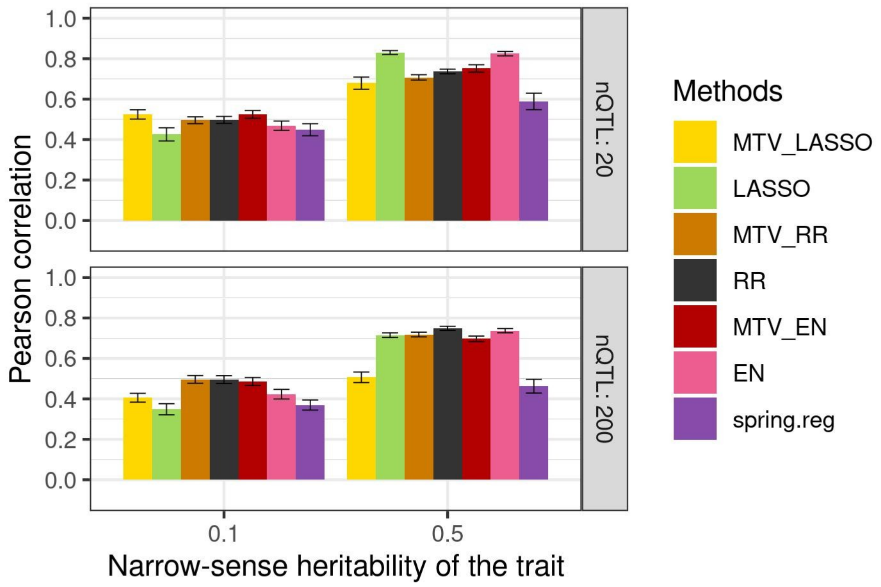

Figure S12: Prediction accuracy with simulated data from Jia & Jannink (2012)

Genomic prediction accuracy (Pearson's correlation between predicted and simulated genotypic values) of seven methods applied to simulated data from Jia and Jannink (2012). The simulated data comprised two traits, with 500 observations for 2000 bi-allelic markers, 20 or 200 QTLs and 24 simulation replicates for each simulation parameter set. The two simulated traits had different heritability values (0.1 and 0.5), a genetic correlation of 0.5, and no error correlation.

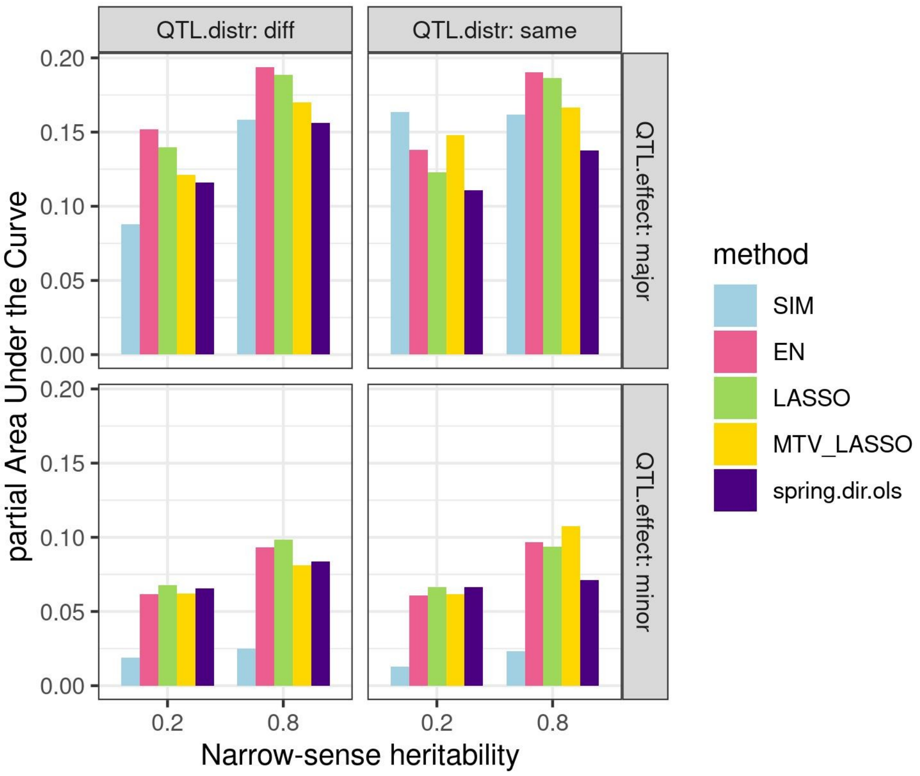

Figure S13: partial Area Under the ROC Curve for FPR up to 0.1

Partial area under the curve (pAUC), calculated from the ROC curve (Figure 2) for FPR ranging from 0 to 0.1, for five methods with FPR reaching 0.1 applied to 3,961 markers and two simulated traits in a bi-parental population, with two heritability values and four QTL configurations (effect x distribution among traits).
major: 2 QTLs; **minor**: 50 QTLs; **same**: QTLs at the same positions for both traits; **diff**: QTLs at different positions between traits.

TableS14_all_results_mean_real-data-pharch.tsv

Table S14: Genomic prediction performance on experimental grapevine data for all traits, and methods

Genomic prediction performance, as measured by different metrics, of 11 methods for 14 traits in a bi-parental grapevine population. Mean results are averaged over 10 cross-validation replicates x five cross-validation folds. As in **Table S9**, the standard error is also given (last columns, suffix "SE") for each metric measured.

TableS15_selected_markers_pharch_all-resp_PVE.tsv

Table S15: Results of marker selection for all traits and methods

genetic.position : genetic position on the SNP map (in cM)
PVE: percentage of variance explained. Marker selection is not extended to LD.

FigureS16_selection_all-resp.pdf

Figure S16: Genetic position of selected markers per trait and for all methods

Each marker selected by a given method is represented by a colored point, the color indicating the number of methods that have selected that specific marker. The boxes correspond to chromosomes and the *x*-axis to the position along the genetic map (in cM). Marker selection is not extended to LD.

FigureS17_MapGBS_most-reliable.pdf

Figure S17: Most reliable QTL intervals displayed along the SNP genetic map

SNP genetic map of the Syrah x Grenache progeny with QTL intervals represented with colored rectangles, hatched for traits under water deficit condition. Trait name and percentage of variance explained are indicated next to intervals. New QTLs are indicated by an asterisk before trait name.

TableS18_reliable_QTLs_information.tsv

Table S18: Complementary information about most reliable QTLs

For each reliable QTL, the table indicates the genetic and physical position from which the QTL interval has been defined, as well as physical and genetic QTL intervals. Methods which have selected the QTL and percentage of variance explained are indicated and we added a logical column (CIM SSR found) which report if the QTL has been published in Coupel-Ledru *et al.* (2014, 2016). If several markers were considered as highly reliable for the same QTL, the one with the largest PVE (from **Table S15**) was kept, then if several markers were at the same PVE, the first one was kept. An interval of physical position may be indicated corresponding to minimum and maximum of markers at the same genetic position.

FigureS19_boxplots_most_reliable_QTLs.pdf

Figure S19: Distribution of genotypic BLUPs according to genotypic class for each highly reliable QTL

Boxplot of genotypic BLUPs according to genotypic class in Syrah x Grenache progeny for the 25 most reliable QTLs. If several markers were considered as highly reliable for the same QTL, the one with the largest PVE (from **Table S15**) was kept, then if several markers were at the same PVE, the first one was kept. QTL position in centiMorgan and percentage of variance explained are indicated. The red line represent the mean of the genotypic BLUP.

TableS20_genes_highly_reliable_QTLs.csv

Table S20: List of genes underlying the most highly reliable QTLs with their annotations

qtl.id: unique identifier of the QTL defined its location chromosome, start and end position in bp.
traits.id: list of traits overlapping in this QTL.
trait.of.interest: trait for which the QTL was determined as highly reliable within the overlapping traits.
gene.loc.in.QTL.peak: yes if the gene is located into the peak of the QTL, no if not.
vitvi.geneid: gene name retrieved from the reference Vitis genome 12X.v2
refseq.geneid: **ncbi.gene.id**, **other.alias**: other identifiers for this gene, retrieved from the VCost.v3 annotation (Canaguier *et al.* 2017).
chr, **start.IGGP12Xv2** and **end.IGGP12Xv2**: chromosome, start position (in bp) and end position (in bp) of the gene on the reference *Vitis* genome 12X.v2.
refseq.genedesc: putative function of the gene retrieved from NCBI, when available.
gene.name: gene name retrieved from TAIR and UniProt, when available.
TAIR.locus: locus in Arabidopsis for the gene's homolog retrieved from TAIR and UniProt, when available.
full.description: complete description of the gene function, retrieved from TAIR and UniProt, when available.
mean.expression.leaves, **min.expression.leaves**, **max.expression.leaves**: mean, min and max expression of the gene in the leaves retrieved from GREAT. Mean, max and min were calculated from all RNA-seq data available on the platform for leaves samples. They are expressed in log(RPKM+1) expression of the gene in the shoots retrieved from GREAT. Mean, max and min were calculated from all RNA-seq data available on the platform for shoots samples.

TableS21_genes_QTL_chr4.csv

Table S21: List and functional classification of annotated genes underlying the highly reliable QTL detected on chromosome 4 for TrS_night.WD_LG4, TE.WW_LG4, TE.WD_LG4, DeltaBiomass.WW_LG4, DeltaBiomass.WD_LG4

qtl.id: unique identifier of the QTL defined its location chromosome, start and end position in bp.
traits.id: list of traits overlapping in this QTL.
vitvi.geneid: gene name retrieved from the reference Vitis genome 12X.v2
refseq.geneid: ncbi.gene.id, other.alias: other identifiers for this gene, retrieved from the VCost.v3 annotation (Canaguier *et al* .2017).
chr, start.IGGP12Xv2 and end.IGGP12Xv2: chromosome, start position (in bp) and end position (in bp) of the gene on the reference Vitis genome 12X.v2.
peak.info: information about the location of the peak of the QTL; "in peak" means that the gene falls into the peak of the QTL; "closest to peak" means that the gene is outside the peak of the QTL but is the closest gene to the peak.
refseq.genedesc: putative function of the gene retrieved from NCBI, when available.
gene.name: gene name retrieved from TAIR and UniProt, when available.
TAIR.locus: locus in Arabidopsis for the gene's homolog retrieved from TAIR and UniProt, when available.
full.description: complete description of the gene function, retrieved from TAIR and UniProt, when available.
mean.expression.leaves, min.expression.leaves, max.expression.leaves: mean, min and max expression of the gene in the leaves retrieved from GREAT. Mean, max and min were calculated from all RNA-seq data available on the platform for leaves samples.
expression of the gene in the shoots retrieved from GREAT. Mean, max and min were calculated from all RNA-seq data available on the platform for shoots samples.
integrated.function: integrated function at the organ or plant level, if it is explicitly quoted in the column "full.description".
cell.or.mol.function: general cellular or molecular function of the gene, when relevant.
constituant.or.specific.molecule: when relevant, name of the constituent or specific molecule related to the cellular or molecular function.
activation.condition: condition triggering the gene activation, when explicitly quoted in the column "full.description".

relation.to.traits.of.interest: « related » if related to the traits in this QTL (*TrS_night, TE, DeltaBiomass*), "unrelated" if not.

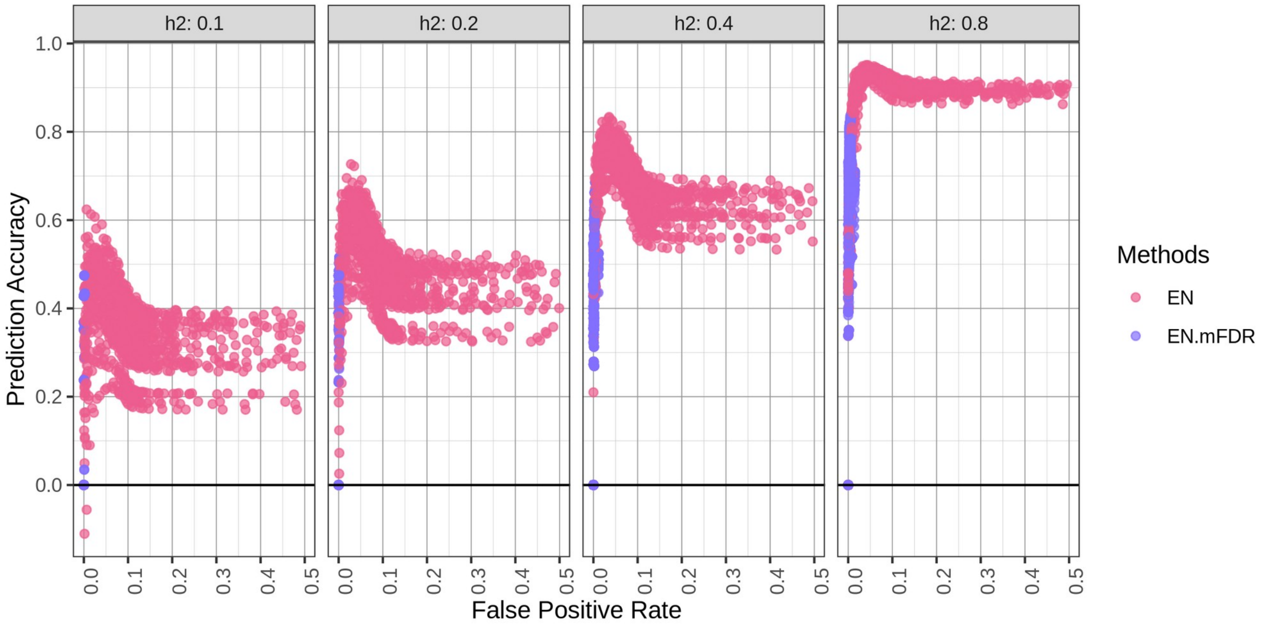

Figure S22: Prediction accuracy according to False Positive Rate for EN and EN.mFDR

For each varying parameter for both methods (i.e. lambda for EN and mFDR for EN.mFDR), we reported the False Positive Rate according to the prediction accuracy, calculated on simulated data, for the two **minor** configurations and the four heritability values.
